# Global prevalence of *Eimeria* species in goats: a systematic review and meta-analysis

**DOI:** 10.3389/fvets.2024.1537171

**Published:** 2025-01-23

**Authors:** Endris A. Ali, Abdul Ghafar, Juan C. Angeles-Hernandez, Muhammad Yaseen, Charles G. Gauci, Ian Beveridge, Sandra Baxendell, Abdul Jabbar

**Affiliations:** ^1^Department of Veterinary Biosciences, Melbourne Veterinary School, Faculty of Science, The University of Melbourne, Werribee, VIC, Australia; ^2^Departamento de Medicina y Zootecnia de Rumiantes, Facultad de Medicina Veterinaria y Zootecnia, Universidad Nacional Autónoma de México, Mexico City, Mexico; ^3^School of Mathematical and Statistical Sciences, Clemson University, Clemson, SC, United States; ^4^Department of Mathematics and Statistics, University of Agriculture, Faisalabad, Pakistan; ^5^Goat Veterinary Consultancies- goatvetoz, Keperra, QLD, Australia

**Keywords:** *Eimeria*, goat, global prevalence, meta-analysis, systematic review

## Abstract

**Background:**

Coccidiosis is a protozoal disease caused by *Eimeria* species, the main symptom of which is diarrhea. *Eimeria* spp. infection can cause weight loss and ill-thrift in goats, and in severe cases, it can lead to mortality in kids, resulting in economic losses for the goat industry. This study aimed to determine the global prevalence of *Eimeria* spp. in goats and to identify the possible predictors of heterogeneity among selected studies.

**Methods:**

Data were retrieved from five databases of major global importance (PubMed, Web of Science, CAB Direct, Scopus, and Google Scholar), with 255 studies published between 1963 and 2022 being included. A random-effects model was used to calculate pooled prevalence estimates with 95% confidence intervals (CI), followed by subgroup meta-analysis and meta-regression analysis to identify factors contributing to high prevalence and explore sources of heterogeneity among studies.

**Results:**

The estimated global prevalence of *Eimeria* spp. in goats was 62.9% (95% CI: 58.6–67.2). Our results indicated high inter-study variability (inconsistency index (I^2^) = 99.7%, *p* < 0.01). Among the variables analyzed, regions and quality of studies were the most significant predictors of heterogeneity. According to the region-based subgroup meta-analysis, North America had the highest estimated prevalence of *Eimeria* spp. (92.2, 95% CI: 82.7–98.2), followed by Europe (86.6, 95% CI: 79.8–92.3), while Asia had the lowest prevalence (52.0, 95% CI: 45.9–58.1). Most countries (*n* = 42/56) had an estimated prevalence above the overall pooled estimate (>62.9%). The subgroup of studies conducted in 2000 or later presented a lower prevalence of 59.6% (95% CI: 54.7–64.3). Studies with a score of 5–7 had a significantly higher prevalence (72.4, 95% CI: 66.2–78.2) than studies with low or medium scores (*p* < 0.01). The prevalence of *Eimeria* spp. in goats detected with conventional and molecular methods was 67.3% (95% CI: 47.0–84.7). Only 47% (119/255) of the studies provided details on identifying *Eimeria* at the species level. Overall, more than 26 *Eimeria* spp. have been identified in goats globally. Among these, the most frequently reported and pathogenic species were *E*. *arloingi* (115/119), *E*. *ninakohlyakimovae* (108/119), *E. christenseni* (94/119), and *E*. *caprina* (71/119). Other valid species that were reported less frequently include *E*. *alijevi*, *E*. *hirci*, *E*. *caprovina*, *E*. *aspheronica* and *E*. *jolchijevi*.

**Conclusion:**

These findings suggest that the pathogenic *Eimeria* spp. are widespread in goats globally. Given the high prevalence and the extensive distribution of pathogenic *Eimeria* spp. in goats, it is recommended that integrated parasite management approaches be implemented for the effective control of coccidiosis in goats.

## 1 Introduction

There are estimated to be more than one billion domestic goats worldwide (1), and the global goat population has more than doubled in the last four decades (2). Despite significant growth in the global goat population, the productivity of the goat industry is challenged by health, management and production constraints (3). Among various health concerns, gastrointestinal diseases (e.g., parasitic gastroenteritis) can lead to significant economic losses for the global goat industry (4).

For instance, coccidiosis, a parasitic disease caused by the intracellular protozoan parasites of the genus *Eimeria* (Apicomplexa: Eimeriidae) (5), is a significant concern for goat farmers due to its economic impact. Globally, several species of *Eimeria* (also known as coccidia) infect goats (6–16), leading to significant economic losses due to poor growth and lower productivity. Although the economic impact of coccidiosis is believed to be substantial (17), there is a lack of data to substantiate such a statement regarding small ruminant coccidiosis.

To date, the number of *Eimeria* spp. that are considered parasites of goats remains variable and controversial (14, 18, 19) and depends on the acceptance of the validity of certain *Eimeria* spp. (12). For instance, Levine (20) reported 13 species of *Eimeria* as the true parasites of goats, including *Eimeria arloingi*, *E. ninakohlyakimovae*, *E. christenseni*, *E. caprina*, *E. caprovina, E. alijevi, E. hirci*, *E. jolchijevi* and *E. aspheronica*, which are distributed globally (6–16). Among these species, *E. arloingi*, *E. ninakohlyakimovae*, *E. christenseni* and *E. caprina* are considered the most pathogenic and prevalent (21), while others are non-pathogenic.

Goats between 1 and 6 months of age are most susceptible to *Eimeria* spp. which inhabit the small and large intestines (22, 23). The oocysts are passed in feces, infecting other animals after further development (i.e., sporulation – asexual reproduction) in the environment. When a susceptible host ingests a sporulated oocyst, the sporozoites are released in the gastrointestinal tract and invade intestinal epithelial cells. Following a number of predetermined generations of development (schizogony – asexual reproduction) in intestinal cells, the female and male gametes form a zygote, which develops into an oocyst (24). The damage to the host occurs due to cell disruption during schizogony and later during gametogony (sexual reproduction). The more prevalent form of coccidiosis is a subclinical disease resulting in poor growth while the clinical form of the disease is most commonly characterized by diarrhea (25). The economic losses due to coccidiosis (26) are attributed to reduced productivity, reduced weight gain, mortality (22, 23) and treatment costs (27). The damage done to the kid’s intestines may be permanent in severe cases, resulting in kids that exhibit ill-thrift for life, i.e., they remain “poor doers” as they fail to recover fully after treatment (25). These kids are more susceptible to other diseases, such as respiratory infections, due to their lower immunity (28).

Given that the global prevalence of *Eimeria* spp. in goats is variable (29–35), with more than 90% prevalence reported in some regions (11, 14, 35–39), effective control measures are essential to minimize losses associated with subclinical and clinical coccidiosis (40). Currently, control of coccidiosis is based on sound management, using preventive medications and treating clinical cases using anticoccidial drugs (23). Although coccidiosis is well-studied in poultry, sheep and cattle, our understanding of goat coccidiosis is limited despite the remarkable growth in the global goat industry in recent decades (14). As a first step to ascertain the current state of play, an exploration of the prevalence and geographical distribution of *Eimeria* spp. in goats could pave the way for global efforts to control goat coccidiosis. Therefore, we conducted a systematic review and meta-analysis to estimate the global prevalence of *Eimeria* spp. infecting goats, with an emphasis on temporal and spatial trends, frequency and spatial distribution of species, diagnostic methods, sample size and quality of selected studies. The findings of this study could be used by veterinarians, researchers, goat farmers and policymakers to make informed decisions about the effective control of coccidiosis in goats worldwide.

## 2 Materials and methods

### 2.1 Search strategy

This study was designed and analyzed according to the Preferred Reporting Items for Systematic Review and Meta-analysis (PRISMA) protocol (41) (Supplementary Table S1). Goats of any age and sex constituted the study population in this study. Two searches across four electronic databases, including PubMed, Scopus, CAB Direct, and Web of Science were performed to retrieve the maximum number of publications. A manual search was also carried out on Google Scholar and reference lists of reviews and included studies. The search queries were designed based on the medical subject headlines (MeSH) and Boolean logic. The MeSH terms and keywords were used to retrieve all relevant articles from the above databases (Supplementary Table S2). The same keywords were used in all electronic databases, and the final database search was completed on March 09, 2023.

### 2.2 Inclusion and exclusion criteria

**Figure 1:**
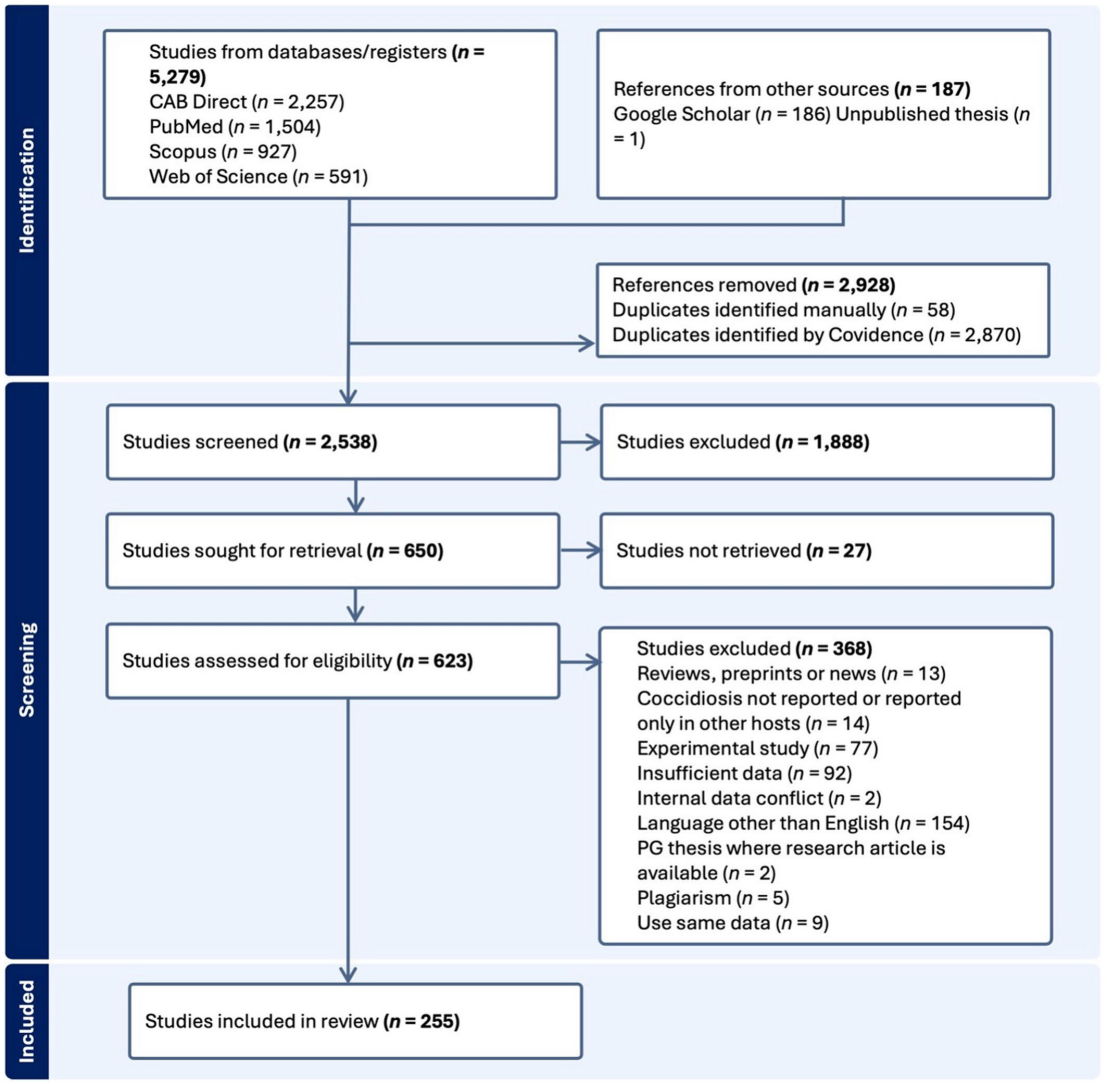
Flow diagram indicating the process of identification, screening and inclusion of studies for the systematic review and meta-analysis according to Preferred Reporting Items for Systematic Reviews and Meta-Analyses (PRISMA) guidelines. PG, postgraduate.

The search results were imported into an online systematic review platform, Covidence.^1^ Duplicate references were then removed, followed by assessment and screening in two steps (Figure 1). The first screening step involved removing irrelevant studies based on the information available from titles and abstracts. In the next step, full texts of selected studies were retrieved. If full texts were unavailable online, they were obtained through interlibrary loans from the University of Melbourne library and were also subjected to the set assessment criteria. To ensure the quality of included studies, the following inclusion criteria were used where (i) the study defined the number of goats examined and the number testing positive, (ii) the study was published in the English language, (iii) the studies contained a full text, (iv) samples indicated the prevalence of *Eimeria* in goats and (v) individual samples were taken from each goat (i.e., samples were not pooled). In addition, we excluded those studies that were not original research articles, involved plagiarism or used the same data for multiple publications, exhibited internal data conflict or insufficient data, only reported other parasites or *Eimeria* reported in host species other than goats or studies with experimental findings only (Figure 1).

### 2.3 Data extraction

The first author, EA, performed the data extraction. Any discrepancies were discussed with co-authors, who acted as secondary reviewers whenever required. The selected studies were coded, and data were collected using a format prepared in a Microsoft Excel^®^ spreadsheet. The format included the first author’s name, research type, title, year of publication, aims of the study, type of diagnostic sample, country of publication and origin of the sample, continent, diagnostic method, study design, sampling period, sampling method, sample size and methods of sample size calculation, the number of goats tested positive, prevalence, age, sex, method of *Eimeria* spp. identification, the identification keys used, and the number of *Eimeria* spp. isolated and/or reported. Data related to molecular methods and markers used for *Eimeria* spp. identification, and sequencing information were also extracted. During the study period, we contacted the authors of some articles to obtain more information and included unpublished data. The extracted data were checked at least twice for accuracy.

### 2.4 Quality assessment

We evaluated the quality of the eligible studies using a scoring method described previously (42–45). The method assessed specific points, including (i) random sampling, (ii) clarity of the detection method described, (iii) detailed description of the sampling method, (iv) inclusion of the sampling period, (v) calculation of sample size, (vi) aim of the study and (vii) species-level identification of *Eimeria*. Each point was scored as one, and articles were assigned to low (0–2 points), medium (3–4), or high (5–7) levels based on their scores (Supplementary Table S3). Data extracted from the studies included were summarized and edited using Microsoft Excel^®^ spreadsheet Version 16.

### 2.5 Statistical analyses

All the analyses and visualization were performed in RStudio 4.3.1 (46) using the “meta” package (47). The bar plots were built using GraphPad Prism 10.2.0.^2^ The mean species richness value was calculated at the country level considering the common nine *Eimeria* spp. of goats reported globally. A random effects model was used to estimate the global pooled prevalence of *Eimeria* spp. of goats, along with a 95% confidence interval (CI) (48). The estimated pooled prevalence was presented as a percentage [(number of positive samples/total samples tested) *100] and displayed using forest plots. To stabilize the variance, we used the Freeman-Tukey double arcsine transformation (referred to as “PFT” in the “meta” package) (49).

Cochran’s Q values and the inconsistency index (I^2^) test statistics were used to assess study heterogeneity. The I^2^ estimates the percentage of variability in effect estimates due to heterogeneity rather than sampling error or chance differences. Therefore, the I^2^ test measures the level of statistical heterogeneity among studies. I^2^ scores of 25, 50 and 75% indicate low, moderate and high degrees of heterogeneity, respectively (50, 51). To evaluate the possibility of publication bias, we utilized funnel plots, Egger’s asymmetry test (52) and Begg’s rank correlation test (53). In the funnel plot, we examined the symmetry of the figure, and if the dots (representing included studies) in the funnel plot were symmetrically distributed on both sides of the mid-line, it indicated no publication bias. If they were asymmetric, it suggested publication bias among the included studies. Begg’s and Egger’s significance tests were also employed to determine the presence of bias. The stability of this study was evaluated by the Duval and Tweedie’s trim and fill analysis (54). Sensitivity analysis was conducted to verify the reliability and robustness of the meta-analysis.

The Baujat plot was used to identify sources of heterogeneity. In the Baujat plot, the horizontal (x) axis represents the contribution of each study to the general statistics of the Cochran’s Q test for heterogeneity, while the vertical (y) axis represents the influence of each study on the overall estimate. The most heterogeneous studies are represented in the upper right area of the graph (55). Subgroup and meta-regression analyses were conducted to investigate the possible sources of heterogeneity. The variables included in the subgroup and meta-regression analyses were year of publication (before 2000 and 2000 or later), region (Asia, Africa, Europe, North America, Oceania and South America), diagnostic methods (conventional, and conventional and molecular methods), the score level of studies (1–2, 3–4 and 5–7), sample size (*n* = <400; *n* = 400–1,000; *n* = *>*1,000) and country (*n* = 56).

Following the subgroup and univariate meta-regression analyses, a multivariate meta-regression analysis was performed on all response variables to identify the best model that explains the between-study variability in effect size estimates. In the meta-regression, the variable x represents study characteristics (such as region, country, year, sample size, score level and detection method), which are used to predict the study effect size ( ${\hat{\theta }}_{\hat{k}}$ ) as shown in the following model (Equation 1):

(1) $${\hat{\theta }}_{\hat{k}}=\theta +\beta x_{k}+\in_{k}+\zeta_{k}$$

Where: ${\hat{\theta }}_{\hat{k}}$ is the observed effect size, θ is the intercept, $\beta x_{k}$ is a predictor (or covariate) $x_{k}$ with a regression coefficient β (fixed effect), $\in_{k}$ is the sampling error and $\zeta_{k}$ is the between-study error (random effect).

The first step in multivariate meta-regression analysis involved all explanatory variables in a full model. Subsequently, multi-predictor models were manually reduced using backward selection of variables until all predictors were statistically significant (*p* < 0.05). We constructed mixed-effects regression models (for the meta-regression analysis). We applied Akaike’s information and Bayesian information criteria to compare and select the models. We assessed the goodness of fit for the meta-regression by calculating the correlation analog coefficient (R^2^) using the following formula (Equation 2):

(2) $$R^{2}=\frac{{\tau^{2}}_{REM-}{\tau^{2}}_{MEM}}{{\tau^{2}}_{REM}}$$

Where: ${\tau^{2}}_{REM}$ represents the estimated total heterogeneity based on the random effects model and ${\tau^{2}}_{MEM}$ represents the total heterogeneity of the mixed effects regression model.

In all analyses, *p*-value <0.05 were used to determine a statistically significant association.

## 3 Results

### 3.1 Characteristics of eligible studies

**Figure 2:**
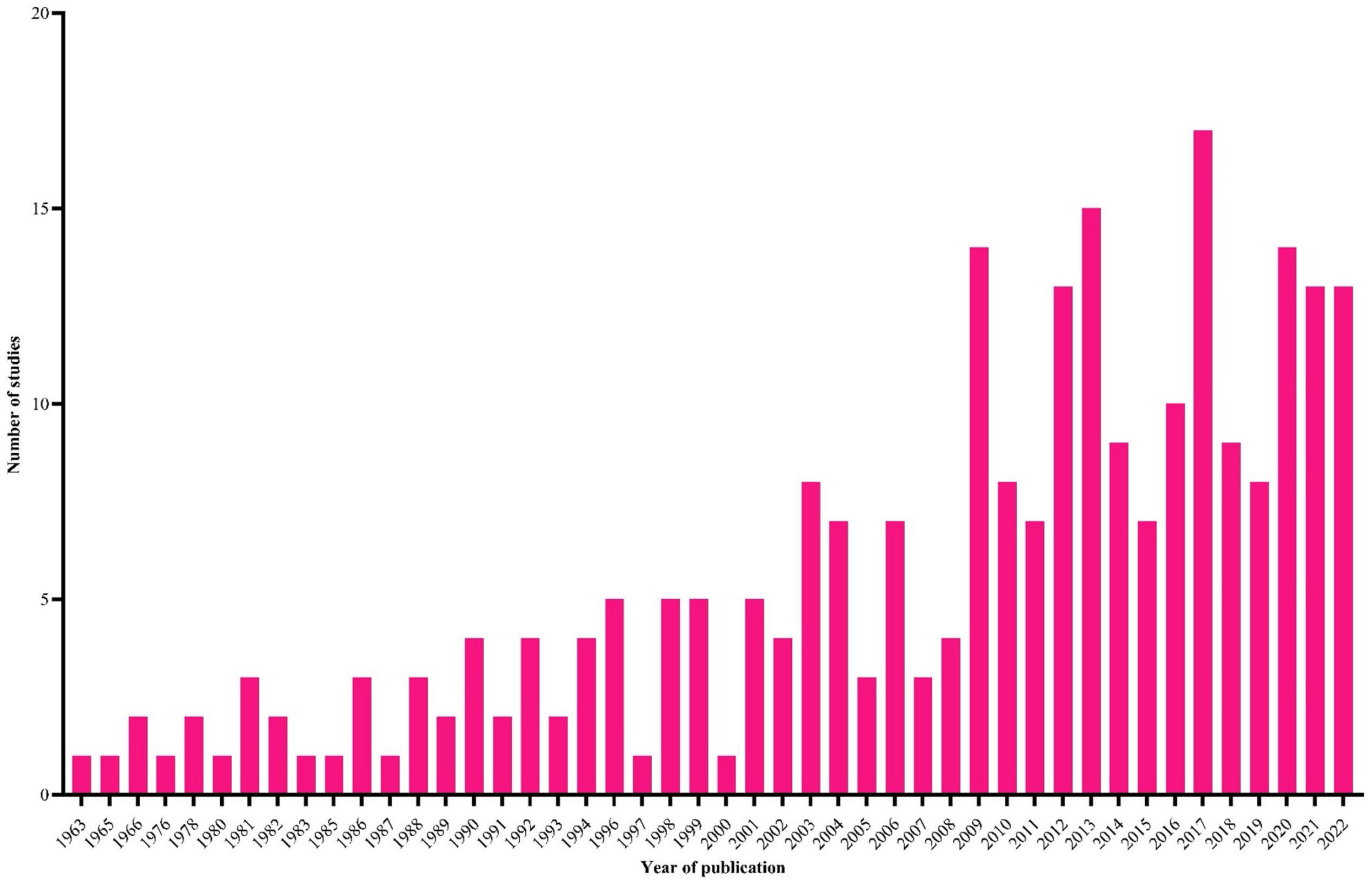
The temporal distribution of studies (*n* = 255) included in this systematic review and meta-analysis.

During the literature search, 5,466 studies were retrieved from five databases (CAB Direct = 2,257, PubMed = 1,504, Scopus = 927, Web of Science = 591 and Google Scholar = 186) and one unpublished thesis, and 255 of them met the inclusion criteria (Figure 1). Most of these studies were original research papers (*n* = 218), followed by short communications (*n* = 27) and postgraduate theses (*n* = 4) published from 1963 to 2022 (Figure 2). An evaluation of the quality of 255 studies showed that 79 scored high (5–7), 149 medium (3–4) and 27 low (1–2) points (Supplementary Table S3).

**Figure 3:**
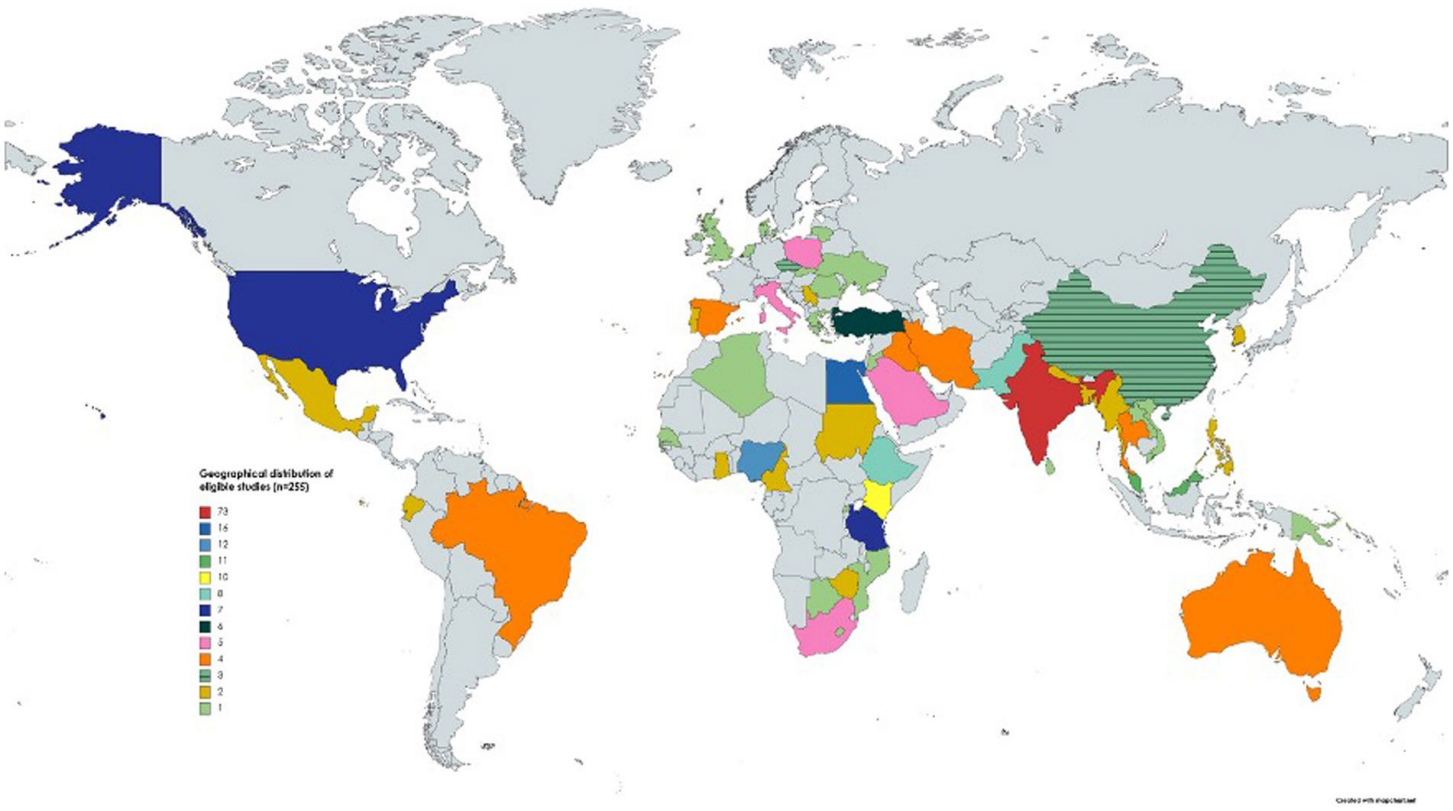
The geographical distribution of studies (*n* = 255) included in this systematic review and meta-analysis. One study was conducted in two countries (see Supplementary Table S4); hence, 256 studies were included in the map.

Two hundred and fifty-five eligible studies originated from 56 countries across six continents (Figure 3) and most of them were from Asia (*n* = 128) followed by Africa (*n* = 72), Europe (*n* = 34) and others (*n* = 21). The eligible studies tested 131,407 goat fecal samples and 75,669 were positive for *Eimeria*. The apparent prevalence in studies ranged from 1.6 to 100% (Supplementary Table S4). Various diagnostic methods were used, including flotation (using saturated sodium chloride, sucrose, sodium nitrate, zinc sulfate and magnesium sulfate), the McMaster method (*n* = 140) in combination with direct smear (*n* = 28), histopathology and/or post-mortem examination (*n* = 11), and molecular tests (*n* = 10) (real-time polymerase chain reaction (qPCR), nested PCR and conventional PCR) (Supplementary Table S4).

**Figure 4:**
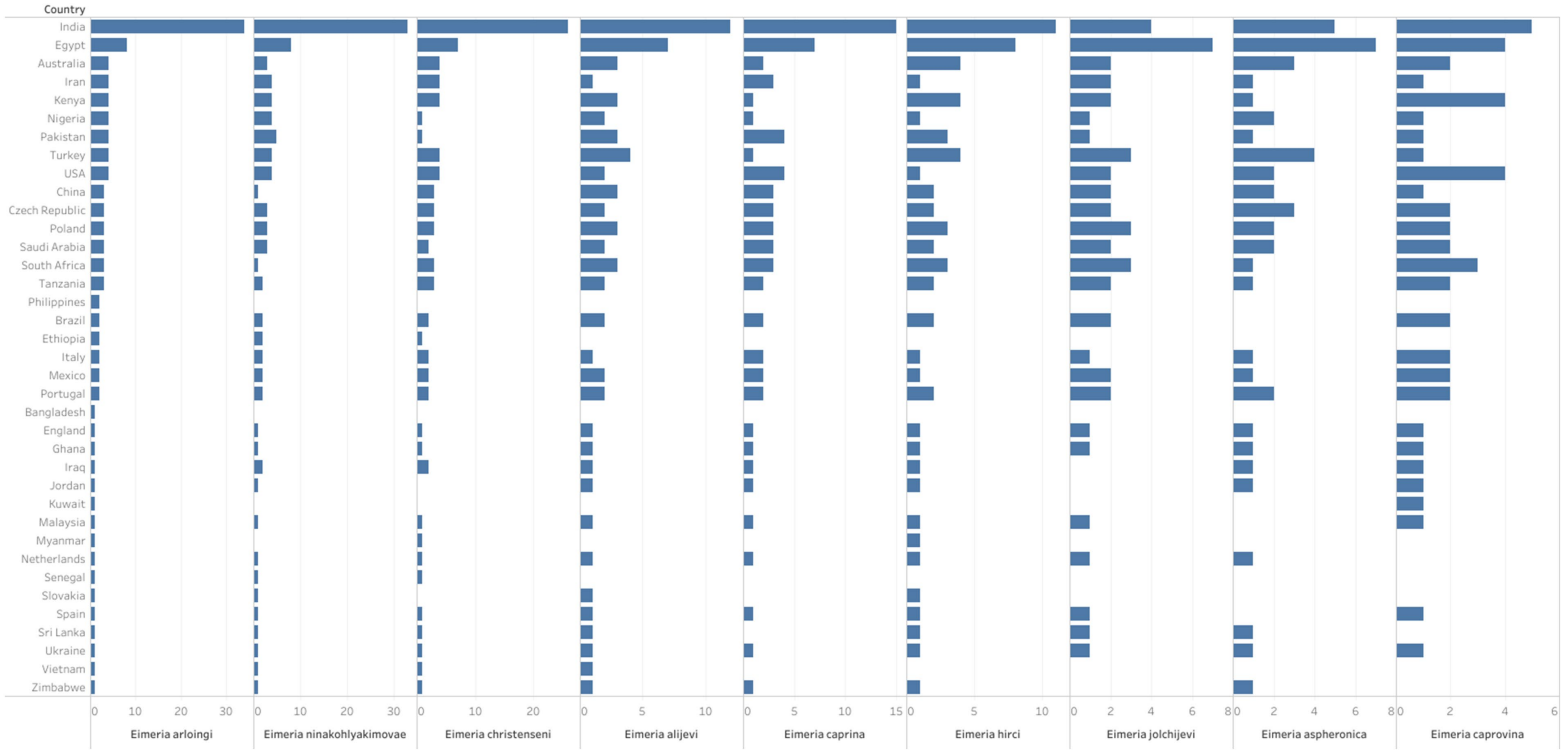
The spatial distribution and diversity of the valid *Eimeria* species commonly reported in goats globally. The length of each bar indicates the number of studies which reported the respective *Eimeria* spp. from each country.

**Figure 5:**
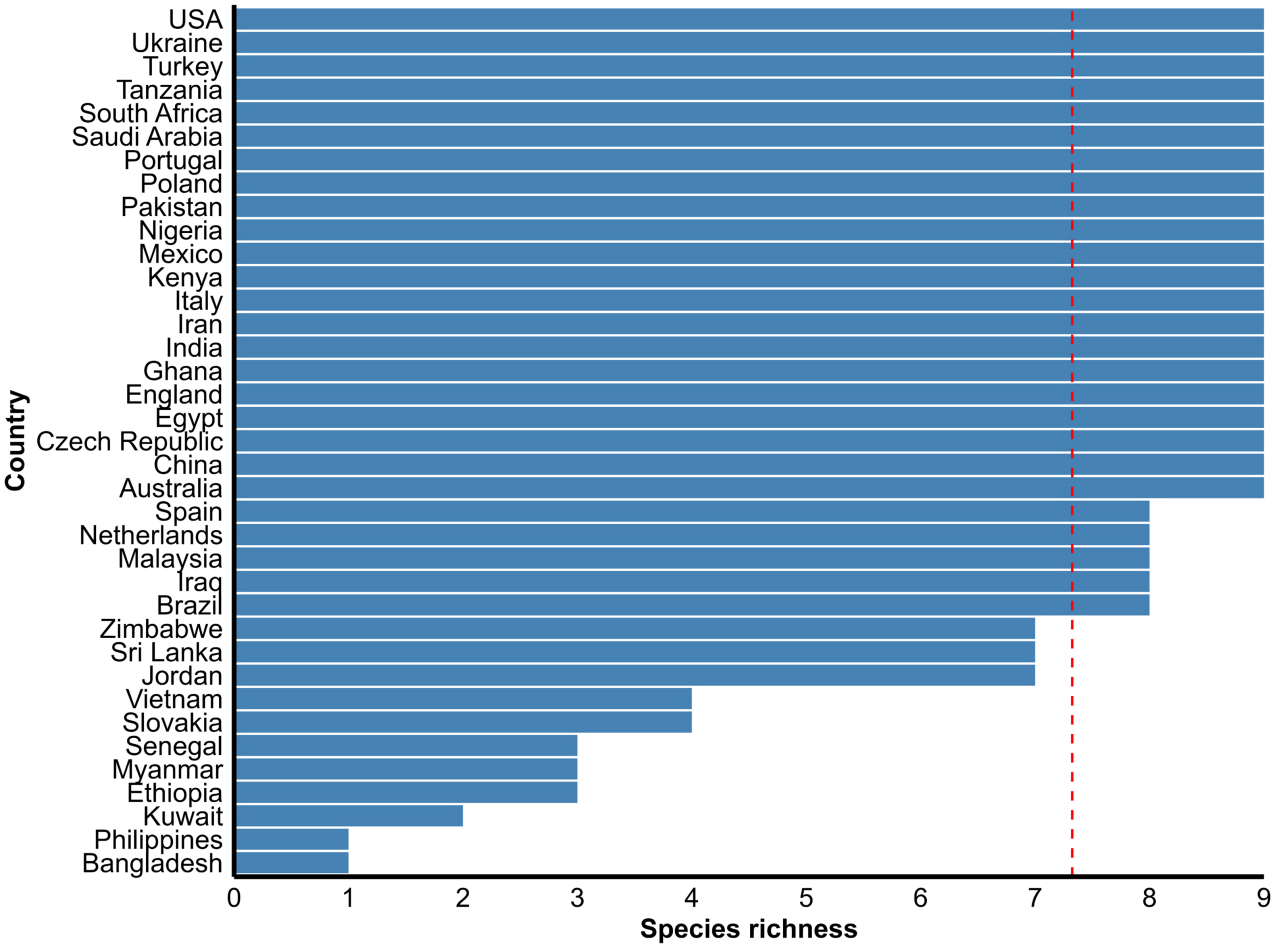
Mean species richness of nine common *Eimeria* species of goats reported globally. The dashed vertical line is the overall mean species richness value. See Supplementary Table S4 for more detail in each country.

**Figure 6:**
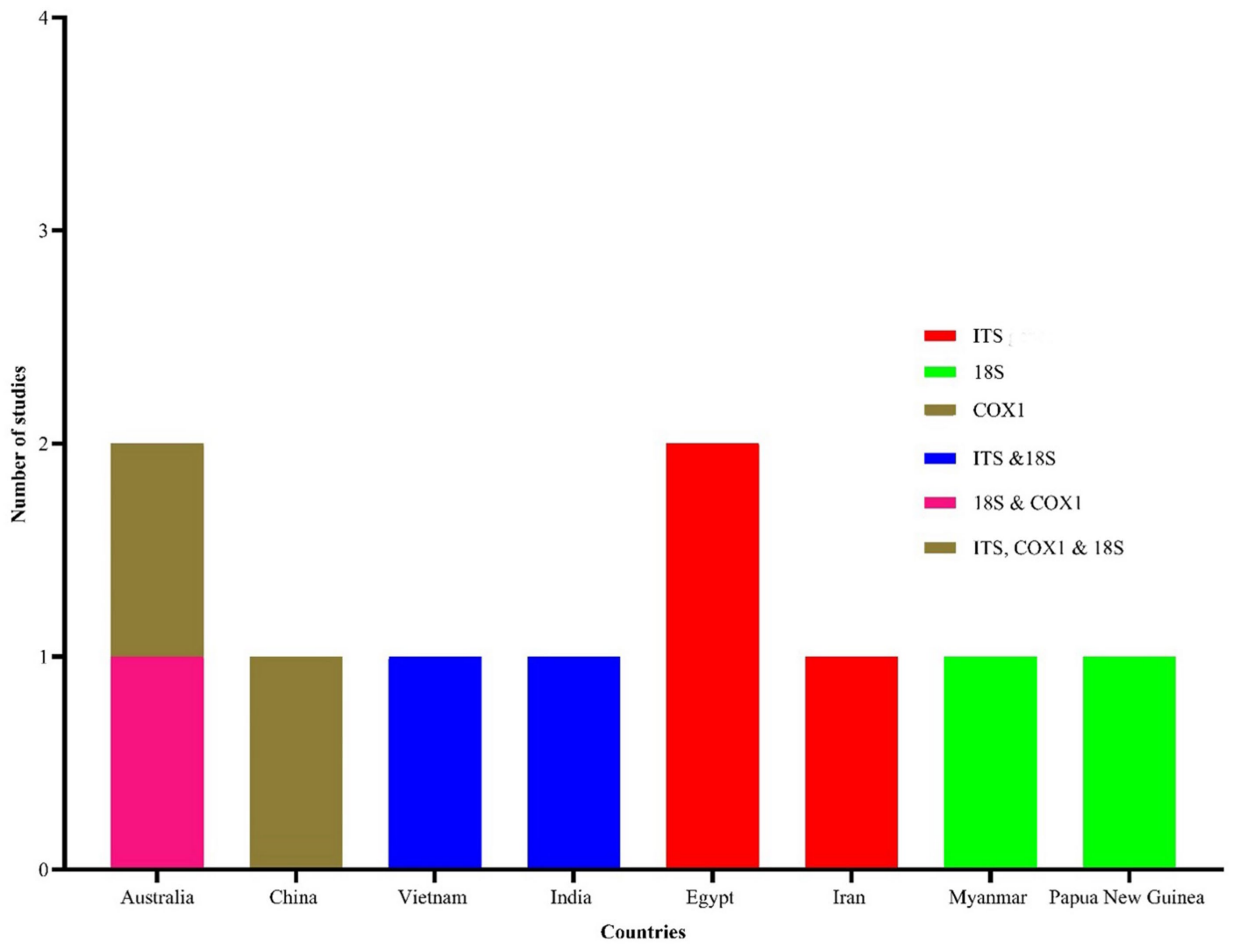
Geographical distribution of studies reporting molecular characterization of *Eimeria* spp. in goats. Three molecular markers, including 18S (small subunit of the nuclear ribosomal RNA), *coxI* (mitochondrial cytochrome *c* oxidase 1) and ITS (the internal transcribed spacer of the nuclear ribosomal DNA) were used in 10 studies.

Morphological identification of *Eimeria* spp. was conducted based on the morphology of sporulated oocysts. For this purpose, fecal samples that tested positive for *Eimeria* were artificially incubated in 2% or 2.5% potassium dichromate for several days (usually 7–10 days) at room temperature. The species of *Eimeria* were then identified using various parameters, including the size and shape index (SI) of oocysts, the presence/absence of a polar cap and micropyle, and the size and SI of sporocysts along with sporulation time. However, only 119 (47%) studies conducted morphological identification of *Eimeria* at the species level using sporulated oocysts and histopathological findings. Globally, more than 26 *Eimeria* spp. (Table 1; Supplementary Table S4) were recorded in goats, with recognized species, including *E*. *arloingi*, *E*. *ninakohlyakimovae*, *E. christenseni*, *E*. *alijevi*, *E*. *caprina*, *E*. *hirci*, *E*. *caprovina*, *E*. *aspheronica* and *E*. *jolchijevi* being the most frequently reported (Figure 4), while other species were reported less frequently (Table 1). Mixed species infections were observed in most studies, with an average or mean species richness value of 7.3 and a median of 8 *Eimeria* spp. reported per study (Figure 5). Only 10 studies from 8 countries corroborated their identification using molecular methods. Molecular-based studies primarily targeted the small subunit of the nuclear ribosomal RNA gene (18S rRNA), followed by mitochondrial cytochrome *c* oxidase 1 (COX1), and/or the first and second internal transcribed spacers of nuclear ribosomal DNA (ITS-1, ITS-2) to genetically characterize four *Eimeria* spp. (*E*. *arloingi*, *E. ninakohlyakimovae*, *E. christenseni*, and *E*. *hirci*) (Figure 6). As of June 25th 2024, more than 70 nucleotide sequences belonging to *E*. *arloingi* (37 sequences), *E. christenseni* (30 sequences), *E*. *hirci* (8 sequences), *E*. *ninakohlyakimovae* (2 sequences), and unidentified *Eimeria* spp. (4 sequences) were available via GenBank^3^. However, only a few of these sequences have been published in peer-reviewed journals.

**Table 1:** The frequency of *Eimeria* spp. of goats reported in the eligible studies.

*Eimeria* species	Frequency
*Eimeria arloingi* ^1^	115
*E. ninakohlyakimovae* ^2^	108
*E. christenseni* ^3^	94
*E. alijevi* ^4^	72
*E. caprina* ^5^	71
*E. hirci* ^6^	70
*E. jolchijevi* ^7^	53
*E. aspheronica* ^8^	51
*E. caprovina* ^9^	50
*E. parva*	29
*E. pallida*	25
*E. faurei*	23
*E. crandallis*	18
*E. granulosa*	17
*E. ahsata*	14
*E. intricata*	13
*E. kocharli*	7
*E. punctuia*	4
*E. parbhaniensis*	3
*E. capralis*	2
*E. hawkin*	2
*E. charlestoni*	2
*E. megaembryonica*	1
*E. tunisiensis*	1
*E. masseyensi*	1
*E. zuernii*	1
Unidentified *Eimeria* spp.^a^	4
Total studies*	119

* Total studies refer to studies in which *Eimeria* was identified and/or reported at species level in goats (see Supplementary Table S4 for more detail).

^1–9 These species are epidemiologically well-established and of major veterinary importance in the goat industry. a Studies from India and Slovakia reported unidentified Eimeria spp.^

### 3.2 Meta-analysis

**Figure 7:**
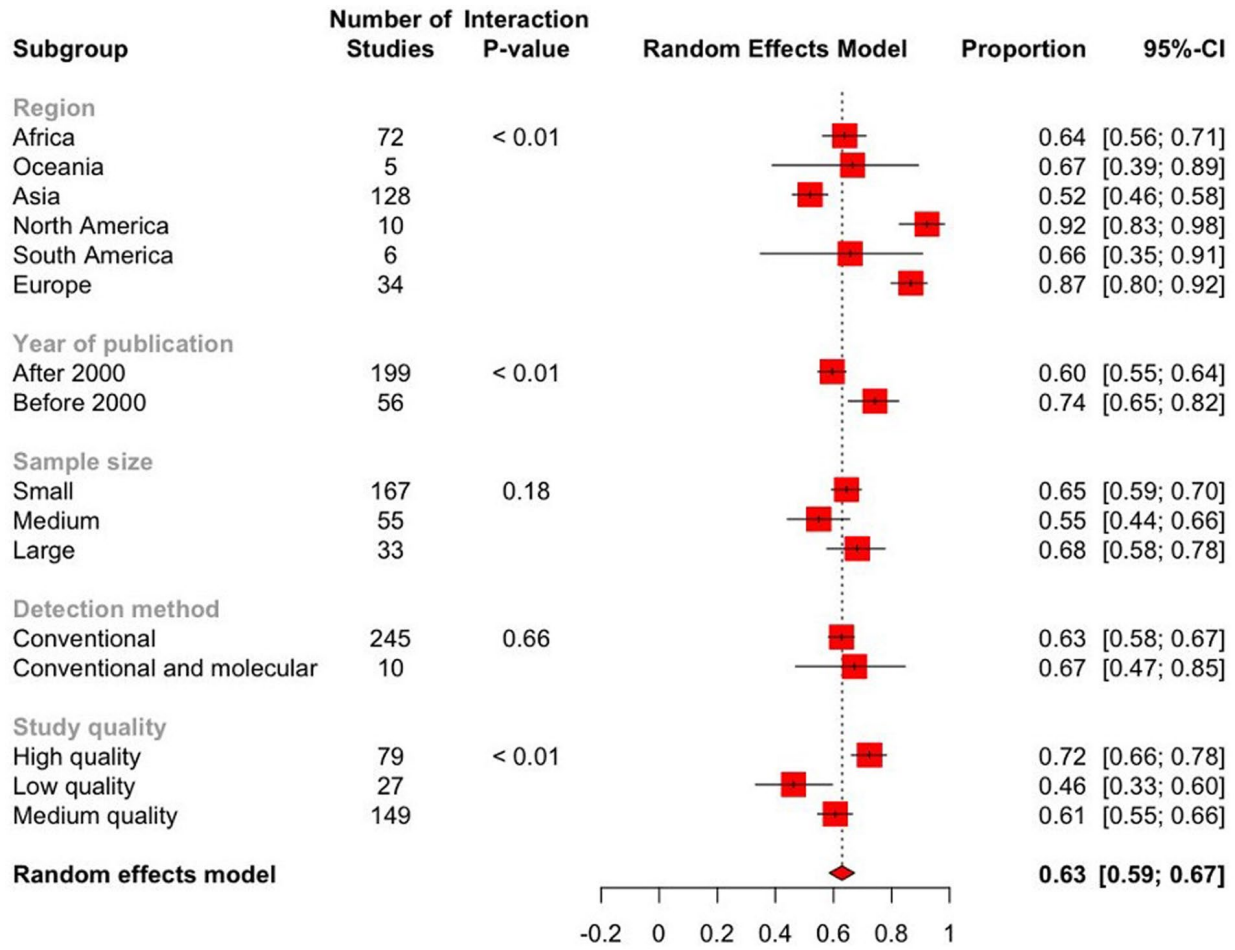
Forest plot displaying the pooled prevalence estimates of *Eimeria* spp. in goats from subgroup meta-analysis. The “Proportion” column shows the prevalence of *Eimeria* spp. in each subgroup, while the 95% CI represents the corresponding confidence interval (CI). The dashed line represents the global pooled prevalence estimate based on the random effects model. The length of the horizontal lines represents the 95% CIs. The estimated global prevalence is the red diamond at the bottom of the plot.

**Figure 8:**
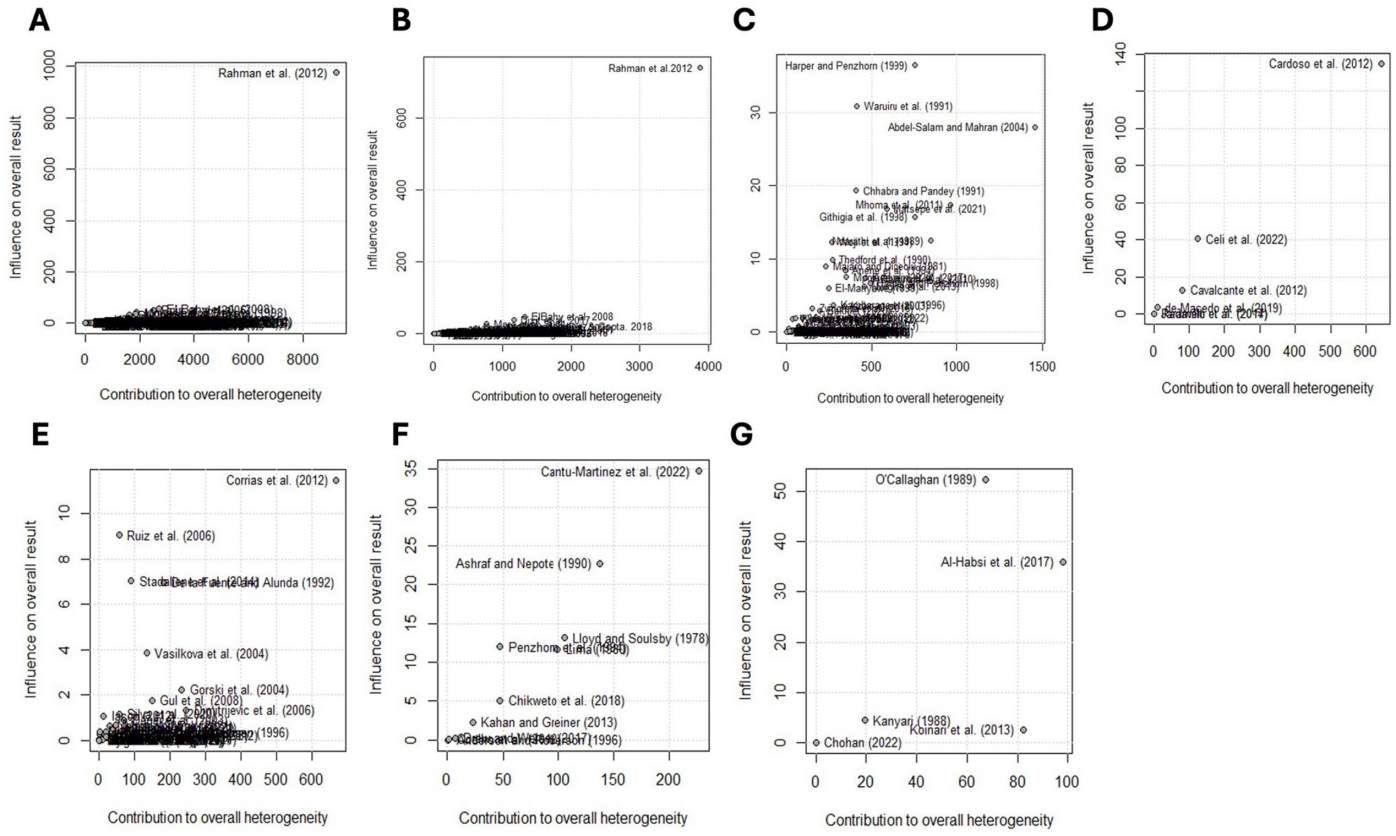
Baujat plots illustrate the contribution of each study to the general statistics of the Q test for heterogeneity. The first graph shows the global pooled prevalence of *Eimeria* spp. **(A)** followed by Asia **(B)**, Africa **(C)**, South America **(D)**, Europe **(E)**, North America **(F)**, and Oceania **(G)**. The studies in the upper right corner have the greatest influence on the results and contribute the most to heterogeneity. Heterogeneity was high (*t*^2^ = 0.1293; *I*^2^ = 99.7%; Cochran’s Q = 72,791.38, *p* = 0.00) for the global prevalence estimate. See Table 2 for general statistics in each region.

A random-effects meta-analysis model was computed using the Freeman-Tukey double-arcsine transformed proportion. This model was chosen due to the expected variation among studies. Based on this model, the estimated global prevalence of *Eimeria* spp. in goats was 62.9% (95% CI: 58.6–67.2) (Figure 7; Table 2). The forest plot revealed high heterogeneity among the studies reporting the prevalence of *Eimeria* spp. ( $\tau^{2}$ = 0.1293, I^2^ = 99.7%, Q = 72,791.38, degrees of freedom = 254, *p* < 0.01). The random effects of studies were weighted similarly to the common (fixed) effects of studies, ranging from 0.0 to 9.6% (see Supplementary Figure S1). The Baujat plot shows that the study conducted by Rahman et al. (56) from India significantly impacted the pooled estimate and contributed the most to the overall heterogeneity (Figure 8A).

**Table 2:** Global pooled prevalence estimates of *Eimeria* spp. infection in goats.

Predictors		Pooled prevalence				Heterogeneity			Test for subgroup differences (REM)	
	Categories	No. of studies	Proportion	% Prevalence (95% CI)	Q	I^2^%	$\tau^{2}$	*p*-value	X^2^	*p*-value
Region	Asia	128	35,570/78,625	52.0 (45.9–58.1)	33,027.96	99.6	0.1216	0.00		
	Africa	72	17,895/27,372	63.9 (56.2–71.2)	12,666.25	99.4	0.1111	0.00		
	Europe	34	17,169/18,929	86.6 (79.8–92.3)	2,396.90	98.6	0.0704	0.00	70.7	< 0.01
	North America	10	2,626/3,032	92.2 (82.7–98.2)	695.10	98.7	0.0526	<0.01		
	South America	6	1,062/1,590	65.7 (34.8–90.7)	857.70	99.4	0.1514	<0.0001		
	Oceania	5	1,347/1,859	66.6 (38.9–89.2)	266.85	98.5	0.1000	<0.01		
Year	Before 2000	56	23,337/32,917	74.3 (65.2–82.4)	15932.30	99.7	0.1411	0.00	7.8	< 0.01
	2000 or later	199	52,332/98,490	59.6 (54.7–64.3)	52271.89	99.6	0.1211	0.00		
Sample size	Large	33	37,125/64,850	68.2 (57.7–77.8)	32625.82	99.9	0.1004	0.00		
	Medium	57	18,905/34,649	55.1 (44.6–65.3)	22092.78	99.7	0.1628	0.00	3.5	0.18
	Small	165	19,639/31,908	64.6 (59.3–69.7)	17569.80	99.1	0.1218	0.00		
Detection methods	Conventional	245	73,337/127,972	62.8 (58.3–67.1)	71497.69	99.7	0.1305	0.00	0.2	0.66
	Conventional & molecular	10	2,332/3,435	67.3 (47.0–84.7)	1152.20	99.2	0.1079	<0.01		
Score levels	1–2	27	4,090/7,455	46.3 (33.2–59.6)	4,123.55	99.4	0.1200	0.00		
	3–4	149	44,077/84,945	60.6 (54.6–66.5)	46,330.97	99.7	0.1414	0.00	15.1	< 0.01
	5–7	79	27,502/39,007	72.4 (66.2–78.2)	16,942.56	99.5	0.0917	0.00		
Overall	-	255	75,669/131,407	62.9 (58.6–67.2)	72,791.38	99.7	0.1293	0.00	-	-

CI, Confidence interval, REM: random effects model, sample size was categorized as small when *n* < 400, medium, *n* = 400–1,000 and large, *n* > 1,000, conventional methods refer to diagnostic tests like floatation, MacMaster, stoll egg counting, direct smear, histopathology, post-mortem, whereas molecular tests include real time polymerase chain reaction (qPCR), nested PCR and conventional PCR. Score level was categorized as low (1–2) medium (3–4) and high (5–7) quality. Q is Cochran’s measure of heterogeneity, $\tau^{2}$ is the variance of the effect size parameters across studies.

### 3.3 Subgroup meta-analysis

**Figure 9:**
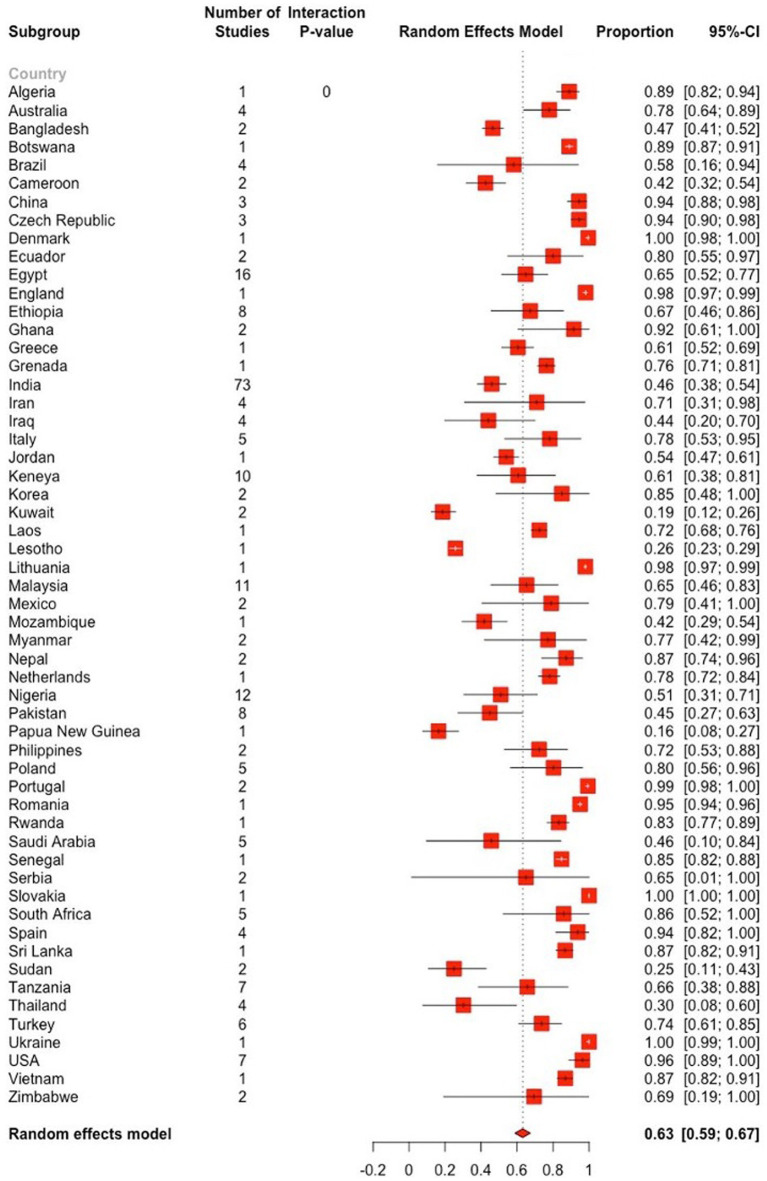
Forest plot displaying the pooled prevalence estimates of *Eimeria* spp. in goats stratified by country. The “Proportion” column shows the prevalence of *Eimeria* spp. in respective country, while the 95% CI represents the corresponding confidence interval (CI). The dashed vertical line denotes the global pooled prevalence estimate, derived from the random effects model. The length of the horizontal line represents the 95% CI. The white and black bars, respectively, denotes a very narrow and wide CIs. The estimated global prevalence is the red diamond at the bottom of the plot.

The subgroup analysis revealed significant heterogeneity between studies in all subgroups. The Baujat plots illustrating the studies that contributed the most to the heterogeneity in each continent are also presented (see Figure 8). The highest prevalence was estimated in North America (92.2, 95% CI: 82.7–98.2) followed by Europe (86.6, 95% CI: 79.8–92.3) while it was the lowest in Asia (52.0, 95% CI: 45.9–58.1) (Figure 7; Table 2). Among the 56 countries included in the eligible studies, 75% (*n* = 42) of them had an estimated prevalence (63–100%) above the overall pooled estimate (>62.9%). On the other hand, Papua New Guinea, Saudi Arabia, and Kuwait reported the lowest estimates with pooled prevalences of 16.4% (95% CI: 7.8–28.8), 19.8% (95% CI: 18.5–21.0) and 19.8% (95% CI: 15.0–25.4), respectively (Figure 9; Supplementary Figure S2).

The prevalence of *Eimeria* spp. in goats sampled before 2000 was 74.3% (95% CI: 65.2–82.4) and 59.6% (95% CI: 54.7–64.3) in 2000 or later, showing a statistically significant downward trend (*p* < 0.05). Based on sample size, the prevalence of *Eimeria* spp. was estimated to be 55.1% (95% CI: 44.6–65.3) in studies with a sample size ranging from 400 to 1,000. In studies with a sample size greater than 1,000, the estimated prevalence was 68.2% (95% CI: 57.7–77.8). However, these differences were not statistically significant (*p* > 0.05). Based on the score level subgrouping, studies with score levels of 5–7 reported the highest prevalences (72.4, 95% CI: 66.2–78.2) compared to low or medium score levels, with statistically significant differences (*p* < 0.01). The prevalence of *Eimeria* spp. in goats detected using conventional and molecular methods was 67.3% (95% CI: 47.0–84.7), which was higher than that detected by conventional methods alone, but these differences were statistically non- significant (Table 2).

### 3.4 Meta-regression models

Univariate and multivariate meta-regression analyses were used to further explore the heterogeneity of data. Among the moderators considered in the univariate analysis, the region, year of publication, sample size and quality level of studies had statistically significant effects on the observed variability between the reports (Table 3). The results of univariate meta-regression indicate that the region was the covariate that contributed the most to explaining the heterogeneity of the total prevalence (*R*^2^ = 15.71). In the multivariate meta-regression, the first approach was to build a full model including all moderators, where almost all covariates were significant (*p* < 0.05) except the detection method. The R^2^ of the full model was 27.3. The best model included the covariates of region, year, sample size, country and score level with an R^2^ of 31.7 (Table 3).

**Table 3:** Univariate and multivariate approach of meta-regression of estimated global pooled prevalence of *Eimeria* spp. in goats.

Model^a^	Covariates^b^	R^2^	Significant levels of covariates
Univariate regression analysis			
Region	Asia	15.71	−0.02*
	North America		+0.58**
	South America		+0.29
	Africa		Reference
	Europe		+0.27***
	Oceania		+0.03
Year	Before 2000		+0.15**
	2000 or later	2.83	Reference
Sample size	Small	0.8	−0.03
	Medium		−0.13*
	Large		Reference
Detection methods	Conventional	0.0	Reference
	Conventional & molecular		+0.04
Score level	Low quality	4.13	−0.26***
	Medium quality		−0.12*
	High quality		Reference
Multivariate regression analysis			
Full model	Region+Year+Sample size+Detection method+Score level + country	27.27	
Best model	Region	31.71	Oceania −0.86***
	Country		Australia +0.65* Kuwait −0.81* Sudan −0.65* Thailand −0.60*
	Year		Before 2000 + 0.18***
	Sample size		Medium sample size −0.21*
	Score level		Low quality −0.19*

a Covariates used in the univariate regression model.

b Levels of the covariates used in the univariate regression models.

R^2^, Amount of heterogeneity accounted for the observed variation.

*p < 0.05, **p < 0.01, ***p < 0.001.

In the univariate regression, a strong positive relationship was observed between the prevalence of *Eimeria* in goats and the European (+0.27***) and North American (+0.58**) regions. Conversely, in the multivariate approach, a negative relationship was found between *Eimeria* prevalence and the Oceania region (−0.86***). In relation to the year of publication of the studies analyzed, both meta-regression approaches yielded similar results, indicating that studies published before 2000 were associated with a higher prevalence of *Eimeria* in goats. Interestingly, the sample size of the studies was identified as a significant factor contributing to the heterogeneity observed in both meta-regression approaches. This was evidenced by a negative relationship between the prevalence level and a medium-sized sample. Finally, the univariate (−0.26***) and multivariate (−0.19*) regression analyses indicated that studies with a lower quality tended to report a lower prevalence of *Eimeria*. However, the univariate regression also identified that medium-quality studies were associated with lower *Eimeria* prevalence (Table 3).

### 3.5 Publication bias assessment

**Figure 10:**
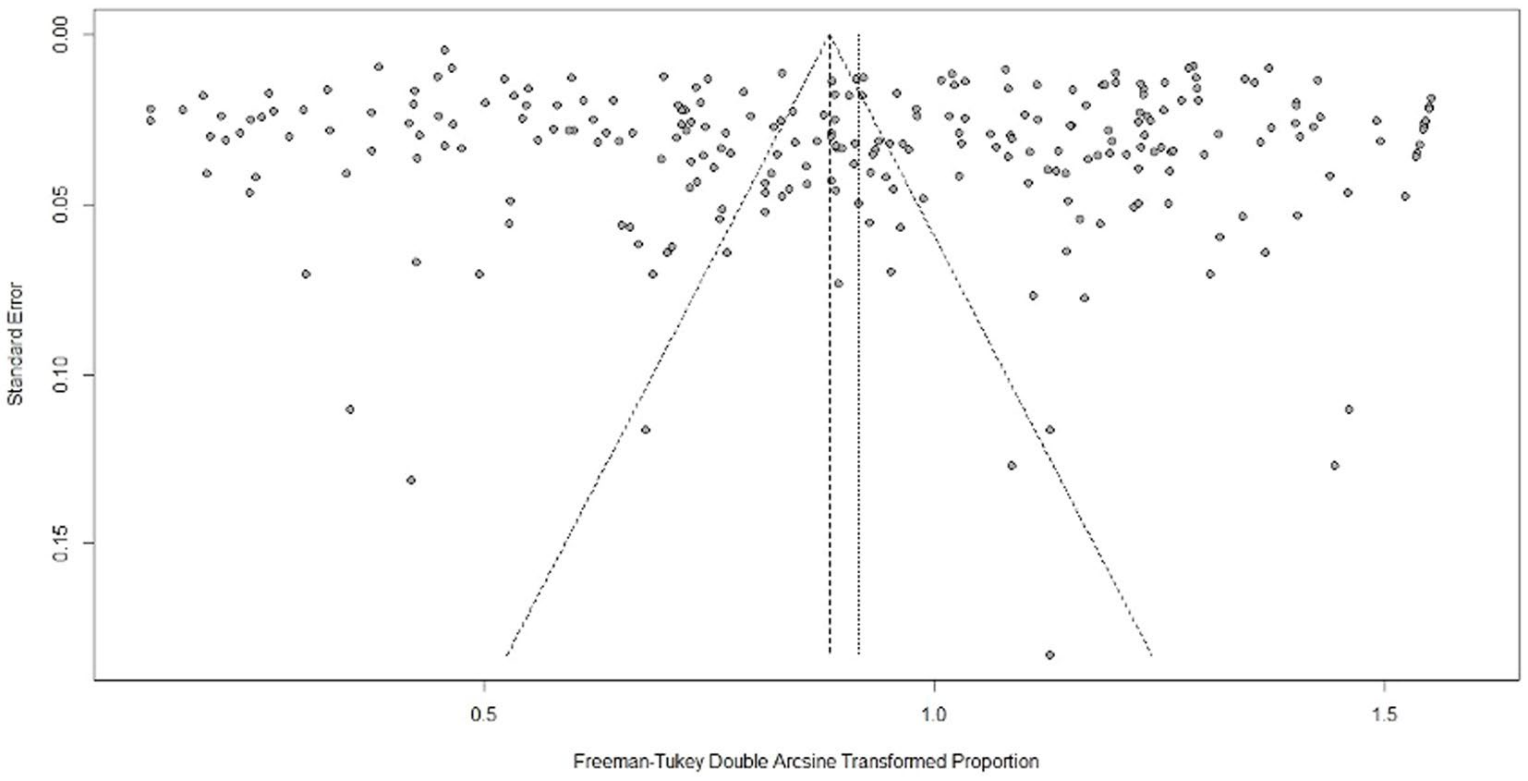
Funnel plot for evaluating publication bias in all included studies. The plot shows each study’s estimated effect plotted against its standard error and evaluates the relationship between study results and their precision.

In the funnel plot, all studies were symmetrically distributed (Figure 10). Additionally, the results of Egger’s regression (*b* = 3.87; *p* = 0.06) and rank correlation tests (*z* = −1.03; *p* = 0.30) for funnel plot asymmetry were not statistically significant for the global pooled estimate (Supplementary Tables S5, S6). Egger’s test did not show significance, nor did the funnel plot exhibit asymmetry for the African (*b* = −4.88; *p* = 0.166) and North American continents (*b* = 3.7479; *p* = 0.736). However, publication bias was significant for the pooled prevalence estimate of Europe (*b* = −5.59; *p* = 0.02) and Asia (*b* = 7.42; *p* = 0.005) (Supplementary Table S7). Funnel plots for each continent are presented in Supplementary Figure S3. Furthermore, there was evidence of missing studies that could be included using Duval and Tweedie’s trim and fill analysis, which would address the asymmetry seen in the plot (Supplementary Figure S4). Eighty-one studies were found in the trim and fill analysis, resulting in a final change to the pooled estimate (Supplementary Figure S5). A sensitivity test showed that the reconstructed data were not affected by the removal of any study, suggesting the rationality and reliability of our analysis (Supplementary Figure S6).

## 4 Discussion

This is the first study on the global prevalence of *Eimeria* spp. infection in goats using a substantial number of studies (*n* = 255) retrieved from five databases. The overall global prevalence of *Eimeria* spp. in goats is 62.9%. The 95% prediction interval (PI) is estimated at 4–100%. The highest prevalence was found in North America (92.2%), followed by Europe (86.6%), while it was the lowest in Asia (52.0%). Surprisingly, in 42 out of 56 countries, the estimated prevalence (63–100%) was higher than the overall pooled estimate (62.9%).

The subgroup analysis by region and country showed that the highest pooled prevalence of *Eimeria* spp. was found in North America (92.2%), and the meta-regression showed a positive beta coefficient (*b* = 0.58). Within this region, the USA had a higher prevalence of *Eimeria* spp. in goats (92.4%), whereas it was lowest in Mexico (71.2%). In the USA, the higher prevalence of *Eimeria* spp. in goats was attributed to risk factors such as season, age, farm management, and the use of deep litter straw bedding materials (57). Additionally, the intensive production system and climate conditions contributed to the higher prevalence of *Eimeria* spp. in the USA (9). However, no studies reported the prevalence of *Eimeria* spp. in goats in other North American countries with significant goat populations such as Haiti and Cuba (1). The study conducted by Cantú-Martínez et al. (58) from Mexico contributed most to the observed heterogeneity on the continent (Figure 8F). Interestingly, no publication bias was detected in this region (Supplementary Table S7).

The estimated prevalence of *Eimeria* spp. in goats in Europe was 86.6%. A meta-regression analysis showed a positive beta coefficient (*b* = 0.27, *p* < 0.01) (Table 2). In Europe, 80% (12/15) of countries had a prevalence of *Eimeria* spp. higher than 70%. The lowest estimated prevalence was 57.1% in Serbia, which is still higher than the estimated prevalence of the Asian continent (52.0%). The goat sector in Europe is specialized in milk production and is highly commercially oriented, dominated by an intensive production system, as reviewed by Miller and Lu (59). This may have contributed to the higher prevalence of *Eimeria* spp. in goats reported in this region, because the intensification of dairy goat production presents challenges in limiting the spread of infectious and parasitic diseases (e.g., coccidiosis), which are facilitated by environmental stressors such as high stocking density (60, 61). A major challenge in commercially oriented dairy goat production systems arises when multiple kidding events occur throughout the year to maintain a consistent milk supply. If the same pens are used repeatedly for successive batches or if newly born kids are introduced to a pen already housing older animals, the later-born kids are immediately exposed to a heavy challenge. They can develop severe coccidiosis in the first few weeks of life (62). Moreover, herd size, age and climatic conditions were associated with varying levels of *Eimeria* spp. prevalence in Europe (40). The studies conducted by Ruiz et al. (40) in Spain and Corrias et al. (63) in Italy influenced the estimated prevalence and contributed to the observed heterogeneity (Figure 8E). We also detected the probability of publication bias in Europe (*b* = −5.59; *p* = 0.02) (Supplementary Table S7; Supplementary Figure S3), which may have further contributed to the heterogeneity.

In Oceania, the estimated prevalence of *Eimeria* spp. in goats was 66.6%. However, it is worth mentioning that almost all studies were conducted in Australia, with limited prevalence data available from countries such as Fiji, New Zealand, Vanuatu, and French Polynesia, which have a significant goat population (64). The studies conducted by O’Callaghan (13) and Al-Habsi et al. (65), had an impact on the pooled estimate and contributed to the heterogeneity (Figure 8G). The number of studies accessible in Oceania was insufficient (< 10) to perform Egger’s test.

In South America, the estimated prevalence of *Eimeria* spp. in goats was 65.7%. The apparent prevalence of *Eimeria* spp. in goats ranged from 4.0 to 91.2%, and almost all studies were conducted in Brazil. This meta-analysis identified the highest prevalence in southern Ecuador and the lowest in Brazil (Supplementary Table S4). The high prevalence of *Eimeria* spp. in goats from Ecuador is often related to the animals’ exposure to risk factors such as age, presence of cattle, type of pasture and body condition (66). In addition, this may be due to the typical situation in goat pens in Ecuador with moist and dark environments, ideal conditions for oocyst sporulation to occur (66). However, countries with a goat population greater than 1 million, such as Bolivia, Peru and Venezuela (1), do not have data on the prevalence of *Eimeria* spp. in goats. A study conducted by Cardoso et al. (67) from Brazil influenced the pooled estimate in this region and contributed to heterogeneity (Figure 8D).

The estimated prevalence of *Eimeria* spp. in goats in Africa was 63.9%. Despite Africa being home to over 40% of the world’s goat population (1), only 72 studies from 17 countries reported the prevalence of *Eimeria* spp. in goats in Africa. The majority of these studies were conducted in five countries, including Egypt (*n* = 16), Nigeria (*n* = 12), Kenya (*n* = 10), Ethiopia (*n* = 8) and Tanzania (*n* = 7) (Figure 5). The highest estimated prevalence of *Eimeria* were found in Algeria (89.0%) and Botswana (89.0%), while the lowest estimate was recorded in Lesotho (26.0%). However, it is important to note that the estimates for these countries were based solely on one study, which may not accurately reflect the true prevalence of *Eimeria* spp. The studies conducted in Africa were more heterogeneous than those of other continents. The heterogeneity was influenced by studies from Egypt (68) and Tanzania (69) (Figure 8C). Egger’s test did not identify publication bias in the prevalence estimates in Africa.

Asia has recognized the importance of dairy goat husbandry in the face of climate change, leading to significant investments in dairy goat projects over the past few decades [see the review by (59)]. This explains the large number of studies (*n* = 128) that report the prevalence of *Eimeria* spp. of goat in Asia. Interestingly, the estimated prevalence of *Eimeria* spp. in Asia, which is home to the world’s largest goat population (1), is 52.0%. This result is significantly lower than the global pooled estimate (Table 2). Moreover, the meta-regression analysis showed a negative beta coefficient (*b* = − 0.02, *p* < 0.05). The highest estimated prevalence in Asia was found in China (95.1%), a global leader in goat populations (1). This result differs from the previous study reported by Diao et al. (42), who estimated the pooled prevalence of *Eimeria* spp. in goats in China to be 78.7% (95% CI: 68.2–87.7%). This discrepancy could be due to the differences in the number of studies (70 studies) and (3 studies in present estimate) included in the meta-analysis and the study periods. Conversely, studies from Saudi Arabia, and Kuwait, Asian countries that reported the lowest prevalence of *Eimeria* spp., were conducted in extensively managed goat flocks and in regions with higher annual average temperatures and lower relative humidity (arid areas) (70, 71). These environmental conditions are known to negatively affect the sporulation and survival of *Eimeria* oocysts in the environment which could be the reason for the lower prevalence. The lower prevalence of *Eimeria* spp. in Saudi Arabia was also attributed to sanitation efforts in management programs introduced by goat producers or ecological differences (8). Most studies used to estimate the prevalence of *Eimeria* spp. in goats in Asia were conducted in India (*n* = 73), Malaysia (*n* = 11) and Pakistan (*n* = 8). However, countries with large goat populations, such as Bangladesh, Indonesia, Mongolia, Nepal and Myanmar (1), had limited or no available data on the prevalence of *Eimeria* spp. In this region, Egger’s test identified statistically significant publication bias (*b* = 7.42; *p* = 0.005) and the greatest contribution to heterogeneity was shared by Rahman et al. (56) (Figure 8B).

It was believed that *Eimeria* spp. in goats and sheep were the same for a long time because their oocysts have strikingly similar morphologies (12, 72). However, cross-infection studies disproved this assumption in the late 20th century (73, 74). In this meta-analysis, we conducted a subgroup analysis based on the year of publication to investigate any discrepancies between studies conducted before and after the assumption was made. We also aimed to identify any changes in the temporal trend of reported cases over time. The prevalence of *Eimeria* spp. in goats sampled before 2000 was 74.3%, whereas in the samples collected in 2000 or later, it was 59.6%, which clearly showed a statistically significant decline (*p* < 0.05). The rationale behind this trend remains uncertain, but it is possible that the previous misconception concerning *Eimeria* spp. in goats and sheep contributed to these differences. Further comprehensive research and scientific justifications are needed to determine whether the decrease in *Eimeria* spp. infection in goats is genuine or not.

The antemortem diagnosis of *Eimeria* spp. infection traditionally relies on the concentration and/or quantification of *Eimeria* oocysts per gram (OPG) in the feces through microscopic examination using flotation techniques and/or the morphometry of sporulated oocysts (24, 75). Most studies (*n* = 202) in this review used fecal flotation techniques to detect and/or enumerate *Eimeria* fecal oocyst count. However, determining a threshold OPG value that indicates clinical coccidiosis is challenging (76). For instance, while some studies suggest that an OPG of 50,000–100,000 could indicate clinical coccidiosis, non-pathogenic *Eimeria* spp. can also be excreted in large numbers without clinical signs (76). Therefore, it is crucial to differentiate between pathogenic and non-pathogenic species to confirm clinical coccidiosis (24).

Our systematic literature review shows that morphological identification is the primary method used to differentiate *Eimeria* spp. in goats. Interestingly, only 47% (119/255) of the studies included in the review documented the identifications of *Eimeria* spp. worldwide. More than 26 *Eimeria* spp. have been reported in goats, including recognized/valid species such as *E. arloingi*, *E. ninakohlyakimovae*, *E. christenseni*, *E. caprina*, *E. caprovina*, *E. alijevi*, *E. hirci*, *E*. *jolchijevi* and *E. aspheronica* as well as other less characterized species. The spatial distribution of the recognized/valid *Eimeria* spp. shows no distinct regional pattern, suggesting their widespread presence (Figure 4). However, there is considerable variation and controversy regarding the number of *Eimeria* spp. parasitising goats (14, 18, 19), and this variation depends on the acceptance of certain *Eimeria* spp. as valid (12). Mixed species infections were commonly observed in most studies, with an average of 7.3 and a median of 8 *Eimeria* spp. reported per study. This review also supports the ongoing controversy that some studies have reported *Eimeria* spp. typically found in sheep (*E. faurei*, *E. crandallis*, and *E. intricata*) and cattle (*E. zuernii*), raising concerns about misinterpretation despite evidence of the absence of cross-host species transmission of *Eimeria* (73, 74). Recent studies in South Korea (77), India (78), and Slovakia (34) described unspecified *Eimeria* spp. Moreover, species such as *E*. *masseyensis* and *E*. *charlestoni* (19) from New Zealand, *E*. *hawkin* and *E*. *charlestoni* (33, 79) from India, *E*. *minasensis* (80) from Brazil, *E*. *sundarbanensis* (81) from India, *E. megaembryonica* (82) from Iraq, and *E. tunisiensis* and *E. masseyensis* (83) from Nepal have been reported despite these species never been previously recorded or described globally. *Eimeria capralis* was first reported in New Zealand (19), with subsequent reports in Iraq (84) and Nepal (83). Furthermore, at least 14 studies reused the same data and/or published their findings twice in different journals, raising significant concerns about the validity of the description of new *Eimeria* spp. or morphological identification of previously described species.

The accurate identification of *Eimeria* spp. is paramount for understanding their epidemiology and assessing the effectiveness of anticoccidial drugs, as only a few *Eimeria* spp. are pathogenic to goats (36, 76, 85). This systematic review showed that the pathogenic species of *Eimeria* (*E. arloingi*, *E. ninakohlyakimovae*, *E. christenseni* and *E. caprina*) are widely distributed. For goat farmers, the widespread presence of these pathogenic *Eimeria* spp., along with a 62.9% estimated prevalence of *Eimeria* spp. poses significant economic losses (42).

Despite the morphological characterization of sporulated oocysts being the primary method for identifying *Eimeria* spp. in goats, this approach has notable limitations, including low sensitivity, the extended time required (1–2 weeks) for oocysts to sporulate under varying conditions (86), labor intensive requirements, requires experienced microscopists (87) and difficulty in differentiating morphologically similar oocysts in certain species of *Eimeria* (12). Although more than 26 *Eimeria* spp. have been documented globally, molecular characterization has only been partially achieved for a few *Eimeria* spp., including *E. arloingi*, *E. christenseni*, *E*. *hirci*, *E*. *ninakohlyakimovae*, and one unidentified *Eimeria* spp. Only a limited number of studies (*n* = 10) across eight countries have used molecular techniques, primarily using PCR amplification of 18S, and/or COX1, and ITS-1 or ITS-2. The lack of combined morphological and molecular methods could lead to the erroneous identification of certain *Eimeria* spp. in goats. As of June 25th, 2024, at least 78 nucleotide sequences have been deposited in GenBank^4^ along with their accession numbers. Most of these sequences were based on partial amplification of 18S gene. Surprisingly, most of these nucleotide sequences (>50%) are not accompanied by peer-reviewed publications, casting doubts on their reliability and validation. Given these challenges, adopting a combined approach using morphological and molecular methods is imperative to accurately identify *Eimeria* spp. More nucleotide sequencing is needed, particularly for *Eimeria* spp. that have yet to be characterized. Furthermore, advanced molecular-based studies that include the genetic characterization of new *Eimeria* spp. and utilizing next-generation sequencing tools could help address the challenges associated with *Eimeria*.

In this systematic review and meta-analysis, there were only 27 low-quality studies with a score of 1–2, but 149 with a score of 3–4. The reasons for fewer points in some studies were: (1) they needed to clarify whether the sampling was random or not, and the sampling method needed to be detailed. (2) Furthermore, neither was the sample size calculated in most studies nor *Eimeria* reported at the species level. When investigating the prevalence of *Eimeria* spp. in goats, we recommend researchers report and/or identify *Eimeria* spp., calculate the sample size, apply representative sampling techniques, and collect and present as much information as possible. Detailed data on potential risk factors, such as age, production systems, study period, and climatic conditions is also quite important. Such data would enhance the understanding of the factors driving *Eimeria* spp. prevalence and allow for more robust risk factor analyses in future meta-analyses.

This meta-analysis has shown high I*^2^* and Cochran’s Q statistics, suggesting significant heterogeneity among the studies reporting the prevalence of *Eimeria* spp. in goats worldwide. The wide range of the 95% PI (4 to 100%) further supports this finding. This variation could be due to several reasons, including geographical factors, differences in production systems, the immune status of the host, differences in the age of goats included in the studies, sample size, diversity of the study populations, breed differences, sampling methods, sex and study periods (88–93). Our meta-regression analyses further showed that region, year of publication, sample size, and the quality level of studies were significant sources of heterogeneity, while the diagnostic methods did not have an impact. Although the improvement in R^2^ was minimal (4.4%) compared to the full and best models, the full model allows for improved balance, fit and parsimony, making it the preferred model that achieves a good fit with fewer predictors. Additionally, the study by Rahman et al. (56) notably influenced the pooled estimate and contributed the most to the overall heterogeneity.

The estimated pooled prevalence reported in this systematic review and meta-analysis should be interpreted with caution due to the following limitations. Firstly, we found high heterogeneity and publication biases (for the Asian and European continents). Although we applied relevant statistical methods, these may not completely eliminate the impact of heterogeneity and publication bias on the interpretation of the pooled results. Secondly, our database search was conducted only in five databases, and the search strategy might have overlooked some research, particularly that published in languages other than English. Thirdly, we excluded a substantial number of studies published in languages other than English, which could introduce potential bias. However, considering the narrow range of the 95% CI, the missed studies are unlikely to significantly affect the present estimate. Furthermore, the wider range of the 95% PI (4–100%) is likely to encompass future primary studies reporting the prevalence of *Eimeria* spp. in goats. Despite these limitations, the authors strongly believe that this systematic review and meta-analysis provide a reliable reflection of the true global prevalence of *Eimeria* spp. in goats.

## 5 Conclusion and future work

This study presents the global prevalence of *Eimeria* spp. in goats based on data collected from approximately 30% (*n* = 56) of countries worldwide. The results of the meta-analysis indicate variations in the prevalence of *Eimeria* spp. in goats globally, with significant heterogeneity observed between studies. Nevertheless, the narrow range of the 95% CI (58.6–67.2%) suggests a precise and reliable estimate of the pooled prevalence of *Eimeria* spp. in goats (62.9%). This finding indicates that the included studies reported similar prevalences, instilling high confidence in the accuracy of the current estimate. However, the wide 95% PI (4–100%) reflects substantial heterogeneity and underscores the need for further studies utilizing advanced molecular tools to resolve the ongoing controversy regarding the number of *Eimeria* spp. parasitising goats. Although a limited number of studies have reported the prevalence of and/or characterized *Eimeria* spp. using molecular data, this technique is known for its sensitivity and accuracy. More sensitive molecular-based approaches, such as next-generation sequencing and genomic analyses based on single oocyst isolation for mixed infections, could offer more precise insights. Our study provides the first meta-analysis of prevalence data on *Eimeria* spp. globally, thereby serving as a valuable reference for the prevention and control of *Eimeria* spp. in goats. Considering the high prevalence and the widespread presence of pathogenic *Eimeria* spp. in goats globally, it is recommended that integrated parasite management approaches be implemented for the effective control of coccidiosis in goats.

## Footnotes

^1^ https://app.covidence.org

^2^ www.graphpad.com

^3^ www.ncbi.nlm.nih.gov

^4^ www.ncbi.com
