# Supplementary material for: Global prevalence of Eimeria species in goats: a systematic review and meta-analysis
Source: Front Vet Sci. 2025 Jan 23;11:1537171. doi: 10.3389/fvets.2024.1537171 (PMC11800301; doi:10.3389/fvets.2024.1537171)
Supplement: Supplementary file 1 [file Table_1.DOCX]

**TABLE S1.** PRISMA 2020 checklist

| **Section and Topic** | **Item #** | **Checklist item** | **Location where item is reported** |
| --- | --- | --- | --- |
| **TITLE** | | |  |
| Title | 1 | **Global Prevalence of *Eimeria* species in goats: a systematic review and meta-analysis** | Page 1 |
| **ABSTRACT** | | |  |
| Structured summary | 2 | Provide a structured summary, as applicable: background | Page 2, 3 |
| **INTRODUCTION** | | |  |
| Rationale | 3 | Describe the rationale for the review in the context of existing knowledge. | Page 3-5 |
| Objectives | 4 | Provide an explicit statement of the objective(s) or question(s) the review addresses. | Page 5 |
| **METHODS** | | |  |
| Eligibility criteria | 5 | Specify the inclusion and exclusion criteria for the review and how studies were grouped for the syntheses. | Page 6 |
| Information sources | 6 | Specify all databases, registers, websites, organisations, reference lists and other sources searched or consulted to identify studies. Specify the date when each source was last searched or consulted. | Page 6, Table S2 |
| Search strategy | 7 | Present the full search strategies for all databases, registers and websites, including any filters and limits used. | Page 6, Table S2 |
| Selection process | 8 | Specify the methods used to decide whether a study met the inclusion criteria of the review, including how many reviewers screened each record and each report retrieved, whether they worked independently, and if applicable, details of automation tools used in the process. | Page 6-8, Figure 1 |
| Data collection process | 9 | Specify the methods used to collect data from reports, including how many reviewers collected data from each report, whether they worked independently, any processes for obtaining or confirming data from study investigators, and if applicable, details of automation tools used in the process. | Page 6, 7 |
| Data items | 10a | List and define all outcomes for which data were sought. Specify whether all results that were compatible with each outcome domain in each study were sought (e.g. for all measures, time points, analyses), and if not, the methods used to decide which results to collect. | Page 6-10 |
|  | 10b | List and define all other variables for which data were sought (e.g. participant and intervention characteristics, funding sources). Describe any assumptions made about any missing or unclear information. | Page 6-10 |
| Study risk of bias assessment | 11 | Specify the methods used to assess risk of bias in the included studies, including details of the tool(s) used, how many reviewers assessed each study and whether they worked independently, and if applicable, details of automation tools used in the process. | Page 7, 8 Table S3 |
| Effect measures | 12 | Specify for each outcome the effect measure(s) (e.g. risk ratio, mean difference) used in the synthesis or presentation of results. | Page 9 |
| Synthesis methods | 13a | Describe the processes used to decide which studies were eligible for each synthesis (e.g. tabulating the study intervention characteristics and comparing against the planned groups for each synthesis (item #5)). | Page 6-10, Figure 1 |
|  | 13b | Describe any methods required to prepare the data for presentation or synthesis, such as handling of missing summary statistics, or data conversions. | Page 8-10 |
|  | 13c | Describe any methods used to tabulate or visually display results of individual studies and syntheses. | Page 8-10 |
|  | 13d | Describe any methods used to synthesize results and provide a rationale for the choice(s). If meta-analysis was performed, describe the model(s), method(s) to identify the presence and extent of statistical heterogeneity, and software package(s) used. | Page 8-10 |
|  | 13e | Describe any methods used to explore possible causes of heterogeneity among study results (e.g. subgroup analysis, meta-regression). | Page 8-10 |
|  | 13f | Describe any sensitivity analyses conducted to assess robustness of the synthesized results. | Page 8-10 |
| Reporting bias assessment | 14 | Describe any methods used to assess risk of bias due to missing results in a synthesis (arising from reporting biases). | Page 8-10 |
| Certainty assessment | 15 | Describe any methods used to assess certainty (or confidence) in the body of evidence for an outcome. | Page 8-10 |
| **RESULTS** | | |  |
| Study selection | 16a | Describe the results of the search and selection process, from the number of records identified in the search to the number of studies included in the review, ideally using a flow diagram. | Page 10, Figures 1 & S3 |
|  | 16b | Cite studies that might appear to meet the inclusion criteria, but which were excluded, and explain why they were excluded. | Figure 1 |
| Study characteristics | 17 | Cite each included study and present its characteristics. | Page 10-11, Table S4 |
| Risk of bias in studies | 18 | Present assessments of risk of bias for each included study. | Page 15, Figures 7,8 & S3 |
| Results of individual studies | 19 | For all outcomes, present, for each study: (a) summary statistics for each group (where appropriate) and (b) an effect estimates and its precision (e.g. confidence/credible interval), ideally using structured tables or plots. | Table 1, Figures 7, 9 |
| Results of syntheses | 20a | For each synthesis, briefly summarise the characteristics and risk of bias among contributing studies. | Tables S3 & S1 |
|  | 20b | Present results of all statistical syntheses conducted. If meta-analysis was done, present for each the summary estimate and its precision (e.g. confidence/credible interval) and measures of statistical heterogeneity. If comparing groups, describe the direction of the effect. | Page 12-14, Tables 1 & 2, Figures 7, 9 & S1 |
|  | 20c | Present results of all investigations of possible causes of heterogeneity among study results. | Page 12, 13 Tables 1 & 2 Figure 8 |
|  | 20d | Present results of all sensitivity analyses conducted to assess the robustness of the synthesized results. | Figure S5 |
| Reporting biases | 21 | Present assessments of risk of bias due to missing results (arising from reporting biases) for each synthesis assessed. | Page 14, 15, Figures 10 & S4, Tables S5-S7 |
| Certainty of evidence | 22 | Present assessments of certainty (or confidence) in the body of evidence for each outcome assessed. | Page 12-15, Figures S5 |
| **DISCUSSION** | | |  |
| Discussion | 23a | Provide a general interpretation of the results in the context of other evidence. | Page 15-23 |
|  | 23b | Discuss any limitations of the evidence included in the review. | Page 22-24 |
|  | 23c | Discuss any limitations of the review processes used. | Page 23, 24 |
|  | 23d | Discuss implications of the results for practice, policy, and future research. | Page 24, 25 |
| **OTHER INFORMATION** | | |  |
| Registration and protocol | 24a | Provide registration information for the review, including register name and registration number, or state that the review was not registered. | Not applicable |
|  | 24b | Indicate where the review protocol can be accessed, or state that a protocol was not prepared. | Not applicable |
|  | 24c | Describe and explain any amendments to information provided at registration or in the protocol. | Not applicable |
| Support | 25 | Describe sources of financial or non-financial support for the review, and the role of the funders or sponsors in the review. | Not applicable |
| Competing interests | 26 | Declare any competing interests of review authors. | Page 25 |
| Availability of data, code and other materials | 27 | Report which of the following are publicly available and where they can be found: template data collection forms; data extracted from included studies; data used for all analyses; analytic code; any other materials used in the review. | Page 25 |

**TABLE S2**. Description of search terms used for database searching.

| **MesH terms** | **Keywords** |
| --- | --- |
| Goats | ((*Eimeria* OR *Eimeria*’s OR Coccidia OR Coccidias OR Coccidiosis) AND |
| *Eimeria* | (Goat OR Goats OR *Capra* OR Capras)), (((Prevalence OR Incidence OR |
| Coccidia | Epidemiology OR Occurrence) AND (Coccidia OR Coccidiosis OR *Eimeria*) |
| Coccidiosis | AND (Goat OR Goats OR *Capra* OR Capras OR Caprine OR Kid))) |

**TABLE S3.** Quality parameters and scores for studies included in this study.

| Reference | No. of faecal samples tested | No. of positive samples | Proportion (%) | Random sampling or not | Detection method clearly described or not | Sampled method detailed enough or not | Sampling time/season clearly mentioned or not | Sample size calculation provided or not | Aim clearly mentioned and achieved or not | *Eimeria* spp. identified and/reported or not | Overall Score | Study quality |
| --- | --- | --- | --- | --- | --- | --- | --- | --- | --- | --- | --- | --- |
| Abdel-Salam and Mahran (2004) | 515 | 8 | 1.55 | Y | Y | Y | N | N | Y | N | 4 | Medium |
| Abdou et al. (2021) | 222 | 46 | 20.72 | Y | Y | Y | Y | Y | Y | N | 6 | High |
| Abdurrahman (2007) | 212 | 175 | 82.55 | Y | Y | Y | Y | N | Y | Y | 6 | High |
| Abebe and Esayas (2001) | 91 | 48 | 52.48 | N | Y | N | Y | N | Y | N | 3 | Medium |
| Abo-Shehada and Abo-Farieha (2003) | 200 | 108 | 54.00 | N | Y | Y | Y | N | Y | Y | 5 | High |
| Abou-El-Naga (2010) | 230 | 139 | 60.40 | Y | Y | Y | Y | N | Y | Y | 6 | High |
| Adams and McKay (1966) | 14 | 2 | 15.00 | Y | N | Y | N | N | N | N | 2 | Low |
| Adeyemi et al. (2017) | 430 | 81 | 18.80 | Y | Y | Y | N | Y | N | N | 4 | Medium |
| Adeyemi et al. (2021) | 51 | 34 | 66.70 | Y | Y | Y | Y | N | Y | Y | 6 | High |
| Agyei et al. (2004) | 20 | 20 | 100.00 | N | Y | Y | Y | N | Y | Y | 5 | High |
| Ahmed et al. (1992) | 210 | 168 | 80.00 | N | Y | N | Y | N | Y | N | 3 | Medium |
| Ahmed (2020) | 380 | 337 | 83.09 | N | Y | Y | Y | N | Y | Y | 5 | High |
| Al-Amery and Hasso (2002) | 615 | 142 | 23.08 | Y | Y | N | N | N | Y | Y | 4 | Medium |
| Al-Bayati et al. (2016) | 77 | 29 | 37.37 | N | Y | Y | N | N | Y | Y | 4 | Medium |
| Al-Habsi et al. (2017) | 500 | 452 | 90.04 | Y | Y | Y | Y | N | Y | Y | 6 | High |
| Alok et al. (2020) | 155 | 141 | 90.96 | N | Y | N | Y | N | Y | Y | 4 | Medium |
| Alyousif et al. (1992) | 228 | 206 | 90.30 | N | Y | Y | Y | N | Y | Y | 5 | High |
| Anderson and Roberson (1996) | 70 | 66 | 94.29 | N | Y | N | Y | N | Y | N | 3 | Medium |
| Anene et al. (1994) | 661 | 217 | 32.82 | Y | Y | Y | Y | N | Y | N | 5 | High |
| Apio et al. (2013) | 7 | 6 | 85.70 | N | Y | N | Y | N | Y | Y | 4 | Medium |
| Arafa (2002) | 288 | 270 | 93.75 | Y | Y | N | Y | N | Y | Y | 5 | High |
| Ashokkumar et al. (2001) | 255 | 149 | 58.43 | Y | Y | N | N | N | Y | Y | 4 | Medium |
| Ashraf and Nepote (1990) | 430 | 297 | 69.06 | Y | Y | Y | Y | N | Y | Y | 6 | High |
| Ashraf et al. (2022) | 166 | 95 | 57.22 | Y | Y | N | N | N | Y | N | 3 | Medium |
| Asif et al. (2012) | 384 | 209 | 54.42 | N | Y | N | N | N | Y | Y | 3 | Medium |
| Asif et al. (2008) | 252 | 92 | 36.50 | N | Y | N | Y | N | Y | N | 3 | Medium |
| Avais et al. (2021) | 376 | 167 | 44.41 | N | Y | N | N | N | Y | Y | 3 | Medium |
| Ayana et al. (2009) | 122 | 54 | 44.26 | Y | Y | Y | Y | Y | Y | Y | 7 | High |
| Azima et al. (2020) | 151 | 97 | 64.23 | N | Y | N | N | N | N | N | 1 | Low |
| Azrul et al. (2017a) | 197 | 90 | 55.55 | Y | Y | Y | Y | N | Y | N | 5 | High |
| Azrul et al. (2017b) | 185 | 76 | 41.08 | Y | Y | Y | Y | N | Y | N | 5 | High |
| Bakhiet (2020) | 400 | 135 | 33.75 | Y | Y | N | Y | Y | Y | N | 5 | High |
| Bakunzia et al. (2010) | 384 | 384 | 100.00 | N | Y | N | Y | N | Y | Y | 4 | Medium |
| Balasubramaniam et al. (2001) | 943 | 96 | 26.01 | N | Y | N | Y | N | Y | N | 3 | Medium |
| Balicka-Ramisz et al. (2012) | 311 | 269 | 86.49 | N | Y | N | Y | N | Y | Y | 4 | Medium |
| Balicka-Ramisz (1999) | 110 | 110 | 100.00 | N | Y | N | Y | N | Y | Y | 4 | Medium |
| Bandyopadhyay (1999) | 50 | 20 | 40.00 | N | Y | N | N | N | Y | N | 2 | Low |
| Bang et al. (1985) | 326 | 314 | 96.32 | N | Y | N | N | N | Y | N | 2 | Low |
| Barua et al. (2009) | 115 | 6 | 5.21 | N | Y | N | Y | N | Y | N | 3 | Medium |
| Bawm et al. (2020) | 280 | 168 | 60.00 | N | Y | Y | Y | N | Y | Y | 5 | High |
| Bersissa et al. (2011) | 65 | 25 | 38.46 | N | Y | N | Y | N | Y | N | 3 | Medium |
| Bhattacharya et al. (1996) | 46 | 28 | 60.86 | Y | Y | N | N | N | N | N | 2 | Low |
| Borgsteede and Dercksen (1996) | 192 | 150 | 78.12 | Y | Y | N | Y | N | Y | Y | 5 | High |
| Buragohain et al. (2013) | 414 | 110 | 26.57 | N | Y | N | Y | N | Y | Y | 4 | Medium |
| Cantu-Martinez et al. (2022) | 403 | 243 | 60.29 | Y | Y | Y | N | N | Y | Y | 5 | High |
| Cardoso et al. (2012) | 276 | 10 | 3.62 | Y | Y | N | Y | N | Y | N | 4 | Medium |
| Carrau et al. (2017) | 84 | 71 | 84.55 | N | Y | N | N | N | Y | N | 2 | Low |
| Cavalcante et al. (2012) | 215 | 196 | 91.2 | N | Y | N | N | Y | Y | Y | 4 | Medium |
| Celi et al. (2022) | 391 | 350 | 89.51 | Y | Y | Y | N | Y | Y | N | 5 | High |
| Chandrawathani et al. (2009) | 203 | 99 | 48.76 | N | Y | N | Y | Y | Y | N | 4 | Medium |
| Chhabra and Pandey (1991) | 1,243 | 1,124 | 90.43 | N | Y | Y | Y | N | Y | Y | 5 | High |
| Chhabra et al. (1983) | 450 | 358 | 79.56 | Y | Y | N | Y | N | N | Y | 4 | Medium |
| Chikweto et al. (2018) | 292 | 223 | 76.00 | Y | Y | Y | Y | N | Y | N | 5 | High |
| Chohan (2022) | 143 | 105 | 73.43 | Y | Y | N | Y | N | Y | Y | 5 | High |
| Coelho et al. (2012) | 202 | 188 | 93.06 | N | Y | N | Y | N | Y | Y | 4 | Medium |
| Corrias et al. (2012) | 320 | 95 | 29.73 | Y | Y | Y | Y | Y | Y | N | 6 | High |
| Das et al. (2006) | 1586 | 1465 | 92.37 | N | Y | Y | N | N | Y | N | 3 | Medium |
| De et al. (2021) | 1,155 | 995 | 86.14 | N | Y | N | Y | N | Y | Y | 4 | Medium |
| De la Fuente and Alunda (1992) | 702 | 702 | 100.00 | N | Y | Y | Y | Y | Y | N | 5 | High |
| Deb et al. (1982) | 500 | 219 | 43.80 | Y | N | N | N | N | Y | Y | 3 | Medium |
| Debele et al. (2013) | 197 | 169 | 8.60 | Y | Y | Y | Y | Y | Y | N | 6 | High |
| Deger et al. (2003) | 242 | 178 | 73.55 | N | Y | N | Y | N | Y | Y | 4 | Medium |
| de-Macedo et al. (2019) | 414 | 306 | 73.91 | N | Y | N | Y | Y | Y | Y | 5 | High |
| Di Cerbo et al. (2010) | 2,554 | 2,348 | 91.94 | Y | Y | Y | Y | N | Y | N | 5 | High |
| Dibyadarshini and Gupta (2018) | 1,200 | 1,066 | 88.83 | N | Y | N | N | Y | Y | N | 3 | Medium |
| Dimitrijevic et al. (2006) | 103 | 26 | 25.24 | N | N | N | N | Y | Y | N | 2 | Low |
| Dixit et al. (2017) | 2,441 | 1,896 | 77.67 | N | Y | N | Y | N | Y | N | 3 | Medium |
| Drew and Weiser (2017) | 88 | 86 | 97.72 | N | Y | N | Y | N | Y | N | 3 | Medium |
| El-Bahy et al. (2008) | 2,689 | 376 | 13.98 | Y | Y | Y | Y | N | Y | N | 5 | High |
| El-Manyawe et al. (2010) | 300 | 220 | 73.33 | N | Y | N | N | N | Y | Y | 3 | Medium |
| El-Manyawe (1999) | 660 | 604 | 91.51 | N | Y | N | Y | N | Y | Y | 4 | Medium |
| El-Shahawy (2016) | 252 | 164 | 65.07 | Y | Y | N | Y | N | Y | Y | 5 | High |
| Etsay et al. (2020) | 187 | 159 | 85.03 | Y | Y | Y | N | Y | Y | Y | 6 | High |
| Faizal and Rajapakse (2001) | 203 | 176 | 86.70 | Y | Y | N | Y | N | Y | Y | 4 | Medium |
| Gadahi et al. (2009) | 310 | 136 | 43.87 | N | Y | N | Y | N | Y | N | 3 | Medium |
| Garcia-Dios et al. (2021) | 103 | 86 | 83.49 | N | Y | N | N | Y | Y | N | 3 | Medium |
| Gebeyehu et al. (2013) | 241 | 163 | 67.63 | N | Y | N | N | Y | Y | N | 3 | Medium |
| Ghanem et al. (2009) | 141 | 93 | 65.95 | N | Y | N | N | N | Y | Y | 3 | Medium |
| Ghimire and Bhattarai (2019) | 400 | 323 | 80.75 | N | Y | N | Y | N | Y | N | 3 | Medium |
| Ghimire et al. (2021) | 991 | 916 | 92.43 | N | Y | N | N | Y | Y | Y | 4 | Medium |
| Githigia et al. (1998) | 560 | 560 | 100.00 | N | Y | Y | Y | N | Y | N | 4 | Medium |
| Githigia (1990) | 161 | 143 | 88.82 | N | Y | Y | Y | N | Y | Y | 5 | High |
| Gorski et al. (2004) | 180 | 80 | 44.44 | N | Y | N | Y | N | Y | Y | 4 | Medium |
| Goz et al. (2006) | 106 | 74 | 69.81 | N | Y | N | Y | N | Y | Y | 4 | Medium |
| Gul et al. (2008) | 223 | 135 | 60.54 | Y | Y | N | Y | N | Y | Y | 5 | High |
| Gupta et al. (2013) | 627 | 285 | 45.45 | N | N | N | Y | N | Y | N | 2 | Low |
| Gwaze et al. (2009) | 224 | 137 | 61.16 | Y | Y | Y | Y | N | Y | N | 5 | High |
| Harper and Penzhorn (1998) | 369 | 369 | 100.00 | Y | Y | Y | Y | N | Y | N | 5 | High |
| Harper and Penzhorn (1999) | 1,263 | 1,206 | 95.49 | N | Y | N | Y | N | Y | Y | 4 | Medium |
| Hashemnia et al. (2015) | 350 | 292 | 83.43 | Y | Y | N | Y | N | Y | Y | 5 | High |
| Hashima and Yusof (2016) | 287 | 256 | 89.20 | Y | Y | N | Y | N | Y | N | 4 | Medium |
| Hassan et al. (2019) | 225 | 173 | 76.89 | Y | Y | N | Y | N | Y | N | 4 | Medium |
| Hassanen et al. (2020a) | 61 | 51 | 83.61 | Y | Y | Y | Y | Y | Y | Y | 7 | High |
| Hassanen et al. (2020b) | 150 | 80 | 53.33 | N | Y | N | Y | N | Y | N | 3 | Medium |
| Hayat et al. (1986) | 260 | 73 | 28.10 | N | Y | N | Y | N | Y | Y | 4 | Medium |
| Hisamuddin et al. (2016) | 227 | 202 | 89.00 | Y | Y | N | Y | N | Y | N | 4 | Medium |
| Holm et al. (2014) | 252 | 251 | 99.60 | N | Y | Y | Y | N | Y | N | 4 | Medium |
| Iacob (2012) | 1,450 | 1,378 | 95.03 | N | Y | N | Y | N | N | N | 2 | Low |
| Ibrahim (2012) | 336 | 180 | 53.57 | Y | Y | N | Y | N | Y | Y | 5 | High |
| Ikpe et al. (2022) | 150 | 5 | 3.33 | Y | Y | N | Y | N | Y | N | 4 | Medium |
| Ikpeze et al. (2009) | 1,120 | 816 | 72.86 | N | Y | N | Y | N | Y | N | 3 | Medium |
| Islam and Taimur (2008) | 224 | 107 | 47.76 | Y | Y | N | Y | N | Y | N | 4 | Medium |
| Jacob and Pillai (1988) | 786 | 497 | 63.23 | N | Y | N | N | N | Y | Y | 3 | Medium |
| Jalila et al. (1998) | 815 | 725 | 89.00 | N | Y | Y | Y | N | Y | Y | 5 | High |
| Jaramillo et al. (2017) | 77 | 52 | 67.53 | N | Y | N | N | Y | Y | N | 3 | Medium |
| Jatau et al. (2011) | 97 | 86 | 88.66 | Y | Y | N | Y | N | Y | N | 4 | Medium |
| Jeyakumar et al. (2009) | 15 | 15 | 100.00 | N | Y | N | N | N | Y | N | 2 | Low |
| Jeyathilakan (2015) | 80 | 20 | 25.00 | N | Y | N | Y | N | Y | N | 3 | Medium |
| Jha and Subramanian (1966) | 243 | 152 | 62.55 | Y | Y | N | N | N | Y | Y | 4 | Medium |
| Jitendra et al. (2017) | 1,437 | 358 | 24.91 | N | Y | Y | Y | N | Y | N | 4 | Medium |
| Junsiri et al. (2021) | 391 | 6 | 1.75 | N | Y | N | Y | N | Y | N | 3 | Medium |
| Juszczak et al. (2019) | 15 | 12 | 80.00 | Y | Y | N | N | N | Y | N | 3 | Medium |
| Kahan and Greiner (2013) | 277 | 270 | 97.00 | N | Y | N | Y | Y | Y | Y | 5 | High |
| Kambarage et al. (1996) | 367 | 357 | 97.27 | N | Y | Y | N | N | Y | N | 3 | Medium |
| Kanyari et al. (2009) | 60 | 29 | 48.33 | Y | Y | Y | N | N | Y | N | 4 | Medium |
| Kanyari (1988) | 346 | 289 | 83.52 | N | Y | N | Y | N | N | Y | 3 | Medium |
| Karimzadeh et al. (2022) | 480 | 64 | 13.33 | N | Y | N | Y | N | Y | Y | 4 | Medium |
| Kaur et al. (2019) | 60 | 58 | 96.67 | Y | Y | N | N | N | N | Y | 3 | Medium |
| Khajuria et al. (2009) | 50 | 4 | 8.00 | Y | Y | N | N | N | N | N | 2 | Low |
| Khan et al. (2015) | 500 | 43 | 8.60 | N | Y | Y | N | N | Y | N | 3 | Medium |
| Kheirandish et al. (2014) | 208 | 187 | 89.91 | Y | Y | N | Y | N | Y | Y | 5 | High |
| Khillare and Narladkar (2014) | 1,663 | 689 | 41.43 | N | Y | N | Y | N | Y | Y | 4 | Medium |
| Kiltu et al. (2016) | 120 | 80 | 66.67 | Y | Y | N | Y | Y | Y | N | 5 | High |
| Kimbita et al. (2009) | 81 | 52 | 64.20 | N | Y | N | N | N | Y | N | 2 | Low |
| Koinari et al. (2013) | 55 | 9 | 16.40 | Y | Y | N | Y | N | Y | Y | 5 | High |
| Koko et al. (2003) | 287 | 49 | 17.07 | N | Y | N | Y | N | Y | N | 3 | Medium |
| Koudela and Bokova (1998) | 2,897 | 2,671 | 92.20 | N | Y | N | Y | Y | Y | Y | 5 | High |
| Krishnamurthy and Kshirsagar (1976) | 659 | 237 | 36.00 | N | Y | N | Y | N | Y | Y | 4 | Medium |
| Kshirsagar (1981) | 1,639 | 307 | 18.73 | N | N | N | Y | N | Y | Y | 3 | Medium |
| Kumar and Balasubramanium (2003) | 250 | 140 | 56.00 | N | Y | N | N | N | Y | N | 2 | Low |
| Kumar et al. (2005) | 435 | 222 | 51.03 | Y | Y | N | Y | N | Y | Y | 5 | High |
| Kusiluka et al. (1996) | 210 | 191 | 91.00 | N | Y | N | Y | N | Y | Y | 4 | Medium |
| Kusiluka et al. (1998) | 995 | 776 | 77.99 | Y | Y | Y | Y | N | Y | Y | 6 | High |
| Kyrianova et al. (2017) | 334 | 327 | 97.90 | N | Y | Y | Y | N | Y | Y | 5 | High |
| Kyrianova et al. (2019) | 659 | 610 | 92.56 | Y | Y | Y | Y | N | Y | Y | 6 | High |
| Laha et al. (2013) | 904 | 152 | 16.81 | N | Y | N | Y | N | Y | N | 3 | Medium |
| Liang et al. (2022) | 245 | 235 | 95.92 | N | Y | N | Y | N | Y | Y | 4 | Medium |
| Lianou et al. (2022) | 119 | 72 | 60.50 | Y | Y | Y | Y | N | Y | N | 5 | High |
| Lima (1980) | 318 | 318 | 100.00 | N | Y | Y | Y | N | Y | Y | 5 | High |
| Lloyd and Soulsby (1978) | 336 | 336 | 100.00 | Y | Y | N | Y | N | Y | Y | 5 | High |
| Lodh and Choudhury (1993) | 500 | 217 | 43.40 | N | Y | N | Y | N | Y | Y | 4 | Medium |
| Lone et al. (2011) | 114 | 60 | 52.63 | Y | Y | N | Y | N | Y | N | 4 | Medium |
| Magi et al. (1986) | 50 | 47 | 94.00 | N | Y | N | Y | N | Y | Y | 4 | Medium |
| Maheshwari et al. (2012) | 300 | 180 | 60.00 | N | Y | N | N | N | Y | N | 2 | Low |
| Mahmoud et al. (2018) | 42 | 34 | 80.95 | N | Y | N | N | N | N | N | 1 | Low |
| Maichomo et al. (2004) | 133 | 60 | 45.11 | Y | Y | N | Y | N | Y | N | 4 | Medium |
| Maingi and Gichohi (1993) | 341 | 157 | 46.04 | N | Y | N | Y | N | Y | N | 3 | Medium |
| Majaro and Dipeolu (1981) | 1,040 | 468 | 45.00 | N | Y | Y | Y | N | Y | Y | 5 | High |
| Majeed et al. (2015) | 20 | 2 | 10.00 | Y | Y | N | N | N | Y | Y | 4 | Medium |
| Mamatha and Souza (2007) | 300 | 145 | 48.33 | Y | Y | N | N | N | Y | N | 3 | Medium |
| Mamta et al. (2013) | 600 | 100 | 16.67 | N | Y | N | Y | N | Y | N | 3 | Medium |
| Maravi et al. (2022) | 1,003 | 273 | 27.22 | N | Y | N | Y | N | Y | N | 3 | Medium |
| Matsepe et al. (2021) | 765 | 197 | 25.75 | Y | Y | Y | Y | N | Y | N | 5 | High |
| Maurizio et al. (2021) | 264 | 207 | 78.41 | Y | Y | Y | Y | Y | Y | N | 6 | High |
| Mazyad and el-Nemr (2002) | 84 | 40 | 47.61 | N | Y | N | Y | N | N | N | 2 | Low |
| Meena et al. (2017) | 834 | 55 | 6.59 | N | Y | N | Y | N | Y | Y | 4 | Medium |
| Mhoma et al. (2011) | 485 | 33 | 6.80 | Y | Y | N | N | N | Y | N | 3 | Medium |
| Miambo (2016) | 60 | 25 | 41.67 | Y | Y | N | Y | Y | Y | N | 5 | High |
| Minnat et al. (2014) | 18 | 15 | 83.33 | N | N | Y | N | N | Y | Y | 3 | Medium |
| Mohamaden et al. (2018) | 135 | 81 | 60.00 | Y | Y | N | Y | Y | Y | Y | 6 | High |
| Mondal and Qadir (1978) | 64 | 27 | 42.18 | N | Y | N | Y | N | Y | Y | 4 | Medium |
| More et al. (2011) | 2,636 | 528 | 20.03 | N | Y | N | N | N | Y | Y | 3 | Medium |
| Mottelib et al. (1992) | 760 | 26 | 3.42 | N | Y | N | Y | N | Y | N | 3 | Medium |
| Mpofu et al. (2020) | 576 | 173 | 30.03 | Y | Y | N | Y | Y | Y | Y | 6 | High |
| Muraleedharan (2005) | 425 | 26 | 6.12 | Y | Y | N | N | N | Y | N | 3 | Medium |
| Nath et al. (2015) | 441 | 261 | 59.18 | N | Y | N | Y | N | Y | N | 3 | Medium |
| Nayak et al. (2021) | 500 | 13 | 2.60 | Y | N | N | Y | N | Y | N | 3 | Medium |
| Ndarathi et al. (1989) | 397 | 22 | 5.54 | N | Y | N | N | N | Y | N | 2 | Low |
| Nenadovic et al. (2021) | 86 | 82 | 95.35 | N | Y | N | Y | N | Y | N | 3 | Medium |
| Ngumbi et al. (2015) | 131 | 69 | 52.67 | Y | Y | Y | Y | Y | Y | Y | 7 | High |
| Nguyen-Ho-Bao et al. (2022) | 251 | 218 | 86.85 | Y | Y | N | N | N | Y | Y | 4 | Medium |
| Nikam (1999) | 575 | 155 | 26.96 | N | N | N | N | N | Y | Y | 2 | Low |
| Norton (1986) | 422 | 414 | 98.10 | N | Y | Y | Y | N | Y | Y | 5 | High |
| Ntonifor et al. (2013) | 94 | 45 | 47.87 | N | Y | Y | N | N | Y | N | 3 | Medium |
| Nur-Rahifah Inani and Yusof (2018) | 480 | 426 | 88.75 | Y | Y | Y | Y | N | Y | N | 5 | High |
| Nwigwe et al. (2013) | 370 | 60 | 16.22 | N | Y | N | Y | N | Y | N | 3 | Medium |
| O'Callaghan (1989) | 815 | 492 | 60.36 | N | Y | Y | Y | N | Y | Y | 5 | High |
| Ola-Davies et al. (2002) | 80 | 69 | 85.00 | N | Y | N | Y | N | Y | Y | 4 | Medium |
| Osman (2009) | 515 | 355 | 68.93 | N | Y | N | Y | N | Y | N | 3 | Medium |
| Padilla et al. (2009) | 173 | 108 | 62.43 | N | Y | Y | Y | Y | Y | Y | 6 | High |
| Padmaja et al. (2007) | 435 | 18 | 4.14 | N | N | N | Y | N | Y | N | 2 | Low |
| Pandit (2006) | 1,877 | 1,362 | 72.56 | N | Y | N | Y | N | Y | Y | 4 | Medium |
| Papri et al. (2017) | 1,921 | 1,047 | 54.51 | N | Y | N | Y | N | Y | N | 3 | Medium |
| Patel et al. (2001) | 142 | 8 | 5.63 | N | N | N | Y | N | Y | N | 2 | Low |
| Patil et al. (1990) | 301 | 106 | 35.21 | N | Y | N | N | N | Y | Y | 3 | Medium |
| Paul et al. (2020) | 257 | 11 | 4.28 | N | Y | Y | Y | Y | Y | N | 5 | High |
| Penzhorn et al. (1994) | 616 | 599 | 97.24 | N | N | N | Y | N | Y | Y | 3 | Medium |
| Pilarczyk et al. (2021) | 158 | 129 | 81.65 | N | Y | N | Y | N | Y | Y | 4 | Medium |
| Priyanka et al. (2013) | 130 | 104 | 80.00 | N | Y | N | Y | N | Y | Y | 4 | Medium |
| Radavelli et al. (2014) | 217 | 148 | 68.20 | Y | Y | N | Y | N | Y | N | 4 | Medium |
| Radfar et al. (2011) | 438 | 391 | 89.27 | Y | Y | N | N | N | Y | Y | 4 | Medium |
| Radha et al. (2004) | 50 | 11 | 22.00 | N | N | N | N | N | Y | Y | 2 | Low |
| Rahman et al. (2012) | 12,604 | 2,445 | 19.39 | N | Y | N | Y | N | Y | N | 3 | Medium |
| Raote et al. (1987) | 769 | 476 | 61.90 | N | N | N | Y | N | N | N | 1 | Low |
| Regassa et al. (2006) | 245 | 84 | 34.28 | Y | Y | Y | Y | N | Y | N | 5 | High |
| Rehman et al. (2012) | 486 | 271 | 55.76 | Y | Y | Y | N | N | Y | Y | 5 | High |
| Rozita et al. (2015) | 218 | 141 | 64.67 | N | Y | N | Y | N | Y | N | 3 | Medium |
| Ruiz et al. (2006) | 2,616 | 2,514 | 96.10 | N | Y | Y | Y | N | Y | Y | 5 | High |
| Sahin et al. (2021) | 110 | 60 | 68.18 | Y | N | N | N | N | Y | N | 2 | Low |
| Saidi et al. (2020) | 100 | 89 | 89.00 | N | Y | N | Y | N | Y | N | 3 | Medium |
| Saleem et al. (2018) | 240 | 159 | 66.25 | Y | Y | N | Y | N | Y | N | 4 | Medium |
| Sangvaranond et al. (2010) | 358 | 155 | 43.29 | N | Y | N | Y | N | Y | N | 3 | Medium |
| Sapna et al. (2010) | 234 | 45 | 19.23 | N | N | N | T | N | Y | N | 2 | Low |
| Secchioni et al. (2016) | 41 | 35 | 85.36 | N | Y | Y | Y | N | Y | Y | 5 | High |
| Seddik et al. (2022) | 100 | 63 | 63.00 | N | Y | N | Y | N | Y | N | 3 | Medium |
| Senthilvel et al. (2004) | 1,545 | 980 | 63.43 | N | Y | N | Y | N | Y | Y | 4 | Medium |
| Shah and Joshi (1963) | 300 | 114 | 38.00 | N | Y | N | N | N | Y | Y | 3 | Medium |
| Shah (1965) | 300 | 15 | 5.00 | N | N | N | Y | N | Y | Y | 3 | Medium |
| Shaheed and Al-Azizz (2020) | 18 | 7 | 38.89 | N | Y | N | Y | N | Y | Y | 4 | Medium |
| Sharma and Mandal (2013) | 1,452 | 672 | 46.28 | N | Y | N | Y | N | Y | Y | 4 | Medium |
| Sharma et al. (2009) | 273 | 117 | 42.86 | N | Y | N | Y | N | Y | Y | 4 | Medium |
| Sharma et al. (1997) | 235 | 235 | 100.00 | N | Y | N | Y | N | Y | Y | 4 | Medium |
| Sharma et al. (2017) | 1,162 | 999 | 86.71 | N | Y | N | Y | N | Y | Y | 4 | Medium |
| Shija et al. (2014) | 129 | 74 | 57.36 | N | Y | Y | Y | N | Y | N | 4 | Medium |
| Shivairo (2003) | 841 | 563 | 66.94 | N | Y | N | N | N | Y | N | 2 | Low |
| Silva et al. (2014) | 144 | 142 | 98.61 | N | Y | Y | N | N | Y | Y | 4 | Medium |
| Silva et al. (2020) | 382 | 380 | 99.00 | Y | Y | Y | N | Y | Y | Y | 6 | High |
| Singh et al. (2013) | 190 | 32 | 16.84 | N | Y | N | Y | N | Y | N | 3 | Medium |
| Singh et al. (2014) | 960 | 803 | 83.65 | N | Y | Y | Y | N | Y | N | 4 | Medium |
| Singh et al. (2017) | 212 | 28 | 16.67 | Y | Y | N | Y | N | Y | N | 4 | Medium |
| Singh et al. (2016) | 153 | 126 | 82.35 | N | Y | N | N | Y | Y | N | 3 | Medium |
| Soliman and Zalat (2003) | 164 | 77 | 46.95 | N | Y | N | Y | N | Y | N | 3 | Medium |
| Sorathiya et al. (2017) | 360 | 72 | 20.00 | Y | Y | Y | N | N | Y | N | 4 | Medium |
| Soundararajan et al. (2019) | 200 | 129 | 64.50 | Y | N | N | Y | N | Y | Y | 4 | Medium |
| Squire et al. (2019) | 285 | 223 | 78.25 | Y | Y | Y | Y | Y | Y | N | 6 | High |
| Stadaliene et al. (2014) | 1,372 | 1,344 | 97.96 | N | Y | Y | Y | N | Y | N | 4 | Medium |
| Tan et al. (2017) | 316 | 99 | 31.32 | N | Y | N | Y | N | Y | N | 3 | Medium |
| Tauseef-ur-Rehman et al. (2011) | 121 | 67 | 55.99 | N | Y | N | N | N | Y | Y | 3 | Medium |
| Tchoumboue et al. (2000) | 79 | 29 | 36.71 | N | Y | N | Y | N | N | N | 2 | Low |
| Terefe et al. (2012) | 192 | 192 | 100.00 | N | Y | Y | Y | N | Y | N | 4 | Medium |
| Thangathurai et al. (2003) | 150 | 17 | 10.67 | N | Y | N | N | N | Y | N | 2 | Low |
| Thedford et al. (1990) | 973 | 866 | 89.00 | N | Y | Y | N | Y | Y | N | 4 | Medium |
| Tiwari et al. (2003) | 1,520 | 480 | 31.57 | N | Y | N | Y | N | Y | N | 3 | Medium |
| Tongson et al. (1981) | 1,127 | 908 | 80.57 | N | Y | N | N | N | Y | Y | 3 | Medium |
| Tumusiime et al. (2022) | 149 | 124 | 83.22 | N | Y | Y | Y | Y | Y | N | 5 | High |
| Varsha et al. (2010) | 1,500 | 940 | 62.67 | Y | Y | N | Y | N | Y | N | 4 | Medium |
| Vasilkova et al. (2004) | 530 | 530 | 100.00 | N | Y | N | N | N | Y | Y | 3 | Medium |
| Vercruysse (1982) | 577 | 489 | 84.75 | N | Y | N | Y | N | Y | Y | 4 | Medium |
| Verma and Jaiswal (2017) | 311 | 32 | 10.29 | N | Y | N | Y | N | Y | N | 3 | Medium |
| Verma et al. (2017) | 1,285 | 949 | 73.85 | N | Y | N | Y | N | Y | Y | 4 | Medium |
| Verma et al. (2018a) | 115 | 114 | 99.13 | N | Y | N | Y | N | Y | Y | 4 | Medium |
| Verma et al. (2018b) | 1,419 | 1,014 | 71.45 | N | Y | N | Y | N | Y | Y | 4 | Medium |
| Vihan et al. (1988) | 205 | 205 | 100.00 | N | Y | N | Y | N | Y | Y | 4 | Medium |
| Wang et al. (2010) | 199 | 175 | 87.94 | Y | Y | Y | Y | N | Y | Y | 6 | High |
| Waruiru et al. (2005) | 315 | 100 | 31.75 | N | Y | Y | Y | N | Y | Y | 5 | High |
| Waruiru et al. (1991) | 1,910 | 1,662 | 87.01 | N | Y | N | Y | N | Y | Y | 4 | Medium |
| Waruiru et al. (1994) | 1,300 | 780 | 60.00 | Y | Y | N | Y | N | Y | Y | 5 | High |
| Win et al. (2020) | 100 | 91 | 91.00 | Y | Y | N | N | N | Y | Y | 4 | Medium |
| Windsor et al. (2018) | 434 | 314 | 72.35 | Y | Y | Y | Y | Y | Y | N | 6 | High |
| Woji et al. (1994) | 1,224 | 1,065 | 87.00 | N | Y | Y | Y | N | Y | Y | 5 | High |
| Wuthijaree et al. (2022) | 885 | 445 | 50.28 | N | Y | Y | Y | Y | N | Y | 5 | High |
| Yadav et al. (2006) | 275 | 21 | 7.63 | N | Y | N | Y | N | Y | N | 3 | Medium |
| Yusof and Isa (2016) | 287 | 256 | 89.20 | Y | Y | Y | Y | N | Y | N | 5 | High |
| Zhao et al. (2012) | 584 | 568 | 97.26 | Y | Y | N | Y | N | Y | Y | 5 | High |
| Zvinorova et al. (2016) | 580 | 249 | 42.93 | Y | Y | Y | Y | Y | N | Y | 6 | High |

**TABLE S4.** Characteristics of selected studies reporting prevalence of *Eimeria* spp. in goats worldwide.

| **Country** | **Diagnostic method(s)** | **Proportion** | **Eimeria spp. reported^*^** | **Quality^1^** | **Reference** |
| --- | --- | --- | --- | --- | --- |
| **South America** | | | | | |
| Brazil | Not provided (NP) | 10/276 | NP | Medium | Cardoso et al. (2012) |
|  | Float (sugar) | 196/215 | 1,2,3,4,5,6,7,8 | Medium | Cavalcante et al. (2012) |
|  | McMaster | 306/414 | 1,2,3,4,5,6,7,8 | High | de-Macedo et al. (2019) |
|  | Float (sugar) | 148/217 | NP | Medium | Radavelli et al. (2014) |
| Ecuador | Float (sugar) and McMaster | 350/391 | NP | High | Celi et al. (2022) |
|  | Float (sugar) and McMaster | 52/77 | NP | Medium | Jaramillo et al. (2017) |
| **North America** | | | | | |
| Grenada | Float (NaCl) and McMaster | 223/292 | NP | High | Chikweto et al. (2018) |
| Mexico | Float (ZnSO₄ and sugar) | 243/403 | 1,2,3,4,5,7,8 and 9 | High | Cantu-Martinez et al. (2022) |
|  | Float (sugar) | 188/202 | 1,2,3,4,5,6,7 and 9 | Medium | Coelho et al. (2012) |
| USA | Float (NaCl) and modified McMaster | 297/430 | 1,2,3, 5,9,10, 12,13,14 and 17 | High | Ashraf and Nepote (1990) |
|  | Float (sugar) | 86/88 | NP | Medium | Drew and Weiser (2017) |
|  | Float (sugar) and McMaster (NaNO3) | 270/277 | 1,2,3,4,5, 6,7,8 and 9 | High | Kahan and Greiner (2013) |
|  | NP | 599/616 | 1,2,3,4,5,7,8 and 9 | Medium | Penzhorn et al. (1994) |
|  | Float (NaNo3) and McMaster | 66/70 | NP | Medium | Anderson and Roberson (1996) |
|  | Float (sugar) and modified McMaster | 318/318 | 1,2,3,5,9,10,13,14 and 17 | High | Lima (1980) |
|  | Float (ZnSO₄ or NaCl) and McMaster | 336/336 | NP | High | Lloyd and Soulsby (1978) |
| **Oceania** | | | | | |
| Australia | Float (NaCl and sugar) and qPCR | 452/500 | 1, 3 and 6 | High | AlHabsi et al. (2017) |
|  | McMaster | 289/346 | NP | Medium | Kanyari (1988) |
|  | Float (MgSo4) | 492/815 | 1,2,3,5,6,7,8, 9 and 12 | High | O'Callaghan (1989) |
|  | Float (NaNo3), modified McMaster, histopathology and PCR | 105/143 | 1,2,3,4,5,6,7,8 and 9 | High | Chohan (2022) |
| PNG | Float, modified McMaster, qPCR, and nested PCR | 9/55 | 26 | High | Koinari et al. (2013) |
| **Europe** | | | | | |
| Czech Republic | Float (sugar), modified McMaster (NaCl) and postmortem | 2,671/2,897 | 1,2,3,4,5,6,7,8 and 9 | High | Koudela and Bokova (1998) |
|  | Float and McMaster | 610/659 | 1,2,3,5,6,7,8,9 and 10 | High | Kyrianova et al. (2019) |
|  | Float (NaCl and sugar) and modified McMaster | 327/334 | 1,2,3,4,5 and 8 | High | Kyrianova et al. (2017) |
| Denmark | Float (NaCl) and modified McMaster | 251/252 | NP | Medium | Holm et al. (2014) |
| England | Modified McMaster | 414/422 | 1,2,3,4,5,6,7,8 and 9 | High | Norton (1986) |
| Greece | Float (ZnSO₄) and direct smear | 72/119 | NP | High | Lianou et al. (2022) |
| Italy | Float | 2,348/2,554 | NP | High | De-Cerbo et al. (2010) |
|  | Float (NaCl) and McMaster | 95/320 | NP | High | Corrias et al. (2012) |
|  | Float and modified McMaster | 47/50 | 1,2,4,5,6,7,8 and 9 | Medium | Magi et al. (1986) |
|  | Float and modified McMaster | 207/264 | NP | High | Maurizio et al. (2021) |
|  | Float (ZnCl) and McMaster | 35/41 | 1,2,3,5 and 9 | High | Secchioni et al. (2016) |
| Lithuania | Modified McMaster | 1,344/1,372 | NP | Medium | Stadaliene et al. (2014) |
| Netherlands | Float (NaCl) and Modified McMaster | 150/192 | 1,2,3,4,5,6,7 and 8 | High | Borgsteede and Dercksen (1996) |
| Poland | Float (Willis-Schlaaf) and McMaster | 110/110 | 1,2,3,4,5,6,7,8 and 9 | Medium | Balicka-Ramisz (1999) |
|  | Float (NaCl) | 80/180 | NP | Medium | Gorski et al. (2004) |
|  | Float (Willis-Schlaaf) and McMaster | 12/15 | NP | Medium | Juszczak et al. (2019) |
|  | Float (Willis–Schlaaf) and McMaster | 129/158 | 1,2,3,4,5,6 and 7 | Medium | Pilarczyk et al. (2021) |
| Poland and Ukraine | Float (Willis-Schlaaf) and McMaster | 269/311 | 1,2,3,4,5,6,7,8 and 9 | Medium | Balicka-Ramisz et al. (2012) |
| Portugal | Modified McMaster (NaCl) | 380/382 | 1,2,3,4,5,6,7,8 and 9 | High | Silva et al. (2020) |
|  | Modified McMaster | 142/144 | 1,2,3,4,5,6,7,8 and 9 | Medium | Silva et al. (2014) |
| Romania | Qualitative and McMaster | 1,378/1,450 | NP | Low | Iacob (2012) |
| Serbia | Qualitative | 26/103 | NP | Low | Dimitrijevic et al. (2006) |
|  | Float (NaCl) and McMaster | 82/86 | NP | Medium | Nenadovic et al. (2021) |
| Slovakia | Float and McMaster | 530/530 | 1,2,4 and 6 | Medium | Vasilkova et al. (2004) |
| Spain | Float (sugar) and McMaster | 71/84 | NP | Low | Carrau et al. (2017) |
|  | Float and modified McMaster | 702/702 | NP | High | De la Fuente and Alunda (1992) |
|  | Float (NaCl) and modified McMaster | 86/103 | NP | Medium | Garcia-Dios et al. (2021) |
|  | Float and modified McMaster | 2,514/2,616 | 1,2,3,4,5,6,7 and 9 | High | Ruiz et al. (2006) |
| Turkey | Float (sugar) | 175/212 | 1,2,3,4,5,6,7,8,9 and 18 | High | Abdurrahman (2007) |
|  | Float (sugar) | 178/242 | 1,2,3,4, 6,7,8,16 and 18 | Medium | Deger et al. (2003) |
|  | Float (sugar) | 74/106 | 1,2,3,4, 6,8 and 18 | Medium | Goz et al. (2006) |
|  | Float | 60/110 | NP | Low | Sahin et al. (2021) |
|  | Float (NaCl) and modified McMaster | 1,465/1,586 | NP | Medium | Das et al. (2006) |
|  | Float (sugar) | 135/223 | 1,2,3,4, 6,7,8 and 18 | High | Gul et al. (2008) |
| **Africa** | | | | | |
| Algeria | Direct smear and float (Willis) | 89/100 | NP | Medium | Saidi et al. (2020) |
| Botswana | Float (NaCl) and McMaster | 866/973 | NP | Medium | Thedford et al. (1990) |
| Cameroon | McMaster | 45/94 | NP | Medium | Ntonifor et al. (2013) |
|  | McMaster | 29/79 | NP | Low | Tchoumboue et al. (2000) |
| Egypt | Float and modified McMaster | 139/230 | 1,2,3,4,5,6,7,8,12 and 15 | High | Abou-El-Naga (2010) |
|  | Float (NaCl) | 8/515 | NP | Medium | Abdel-Salam and Mahran (2004) |
|  | Float | 270/288 | 1,2,3,4,5,6,7 and 8 | High | Arafa (2002) |
|  | Float and McMaster | 604/660 | 1,2,3,4,5,6,7,8 and 9 | Medium | El-Manyawe (1999) |
|  | Float and McMaster | 220/300 | 1,2,3,4,5,6,7,8 and 9 | Medium | El-Manyawe et al. (2010) |
|  | Float (NaCl) and McMaster | 164/252 | 1,2,4,6,7,8 and 9 | High | El-Shahawy (2016) |
|  | Float, modified McMaster and histopathology | 93/141 | 1,2,3,4,5 and 6 | Medium | Ghanem et al. (2009) |
|  | Float (NaCl) and PCR | 51/61 | 1,2,3,4,5,6,7,8 and 9 | High | Hassanen et al. (2020a) |
|  | Float (NaCl) and direct smear | 80/150 | NP | Medium | Hassanen et al. (2020b) |
|  | Postmortem and float | 173/225 | NP | Medium | Hassan et al. (2019) |
|  | Float, histopathology and postmortem. | 34/42 | NP | Low | Mahmoud et al. (2018) |
|  | Direct smear and float | 40/84 | NP | Low | Mazyad and el-Nemr (2002) |
|  | Float (NaCl), modified McMaster and PCR | 81/135 | 1,2,3,5,6,7 and 8 | High | Mohamaden et al. (2018) |
|  | Float (NaCl) and McMaster | 355/515 | NP | Medium | Osman (2009) |
|  | Direct smear and float (sugar) | 63/100 | NP | Medium | Seddik et al. (2022) |
|  | Direct smear and float (sugar) | 77/164 | NP | Medium | Soliman and Zalat (2003) |
| Ethiopia | Float (NaCl) and McMaster | 48/91 | NP | Medium | Abebe and Esayas (2001) |
|  | Float (NaCl) | 25/65 | NP | Medium | Bersissa et al. (2011) |
|  | Float and McMaster | 192/192 | NP | Medium | Terefe et al. (2012) |
|  | Float (sugar) | 54/122 | 1,2 and 14 | High | Ayana et al. (2009) |
|  | Float (sugar) and modified McMaster | 159/187 | 1,2 and 3 | High | Etsay et al. (2020) |
|  | Direct smear and float | 84/245 | NP | High | Regassa et al. (2006) |
|  | Float and McMaster | 169/197 | NP | High | Debele et al. (2013) |
|  | Float (NaCl) | 80/120 | NP | High | Kiltu et al. (2016) |
| Ghana | Postmortem, float and modified McMaster | 20/20 | 1,2,3,4,5,6,7,8,9 and 18 | High | Agyei et al. (2004) |
|  | Float (formal ether) | 223/285 | NP | High | Squire et al. (2019) |
| Kenya | Float (MgSO4) | 145/161 | 1,2,3,4,5,6,7,8 and 9 | High | Githigia (1990) |
|  | Direct smear, float and modified McMaster | 29/60 | NP | Medium | Kanyari et al. (2009) |
|  | Modified McMaster | 60/133 | NP | Medium | Maichomo et al. (2004) |
|  | McMaster | 157/341 | NP | Medium | Maingi and Gichohi (1993) |
|  | Float (sugar) and McMaster | 22/397 | NP | Low | Ndarathi et al. (1989) |
|  | McMaster | 563/841 | NP | Low | Shivairo (2003) |
|  | Float (NaCl) and McMaster | 1,662/1,910 | 1,2,3,4,6,7 and 9 | Medium | Waruiru et al. (1991) |
|  | Float (MgSo4) and modified McMaster | 100/315 | 1,2,3,4,6,8 and 9 | High | Waruiru et al. (2005) |
|  | Modified McMaster | 780/1300 | 1,2,3,4,5,6,7,8 and 9 | High | Waruiru et al. (1994) |
|  | Float (NaCl) and modified McMaster | 560/560 | NP | Medium | Githigia et al. (1998) |
| Lesotho | Float (NaCl) and McMaster | 197/765 | NP | High | Matsepe et al. (2021) |
| Mozambique | Float (NaCl) and McMaster | 25/60 | NP | High | Miambo (2016) |
| Nigeria | Float (NaCl) | 34/51 | 1,2,3,4,5,6,8 and 9 | High | Adeyemi et al. (2021) |
|  | Float (sugar) and McMaster | 81/430 | NP | Medium | Adeyemi et al. (2017) |
|  | Float (sugar) | 2/14 | NP | Low | Adams and McKay (1966) |
|  | Float (NaCl)) and McMaster | 217/661 | NP | High | Anene et al. (1994) |
|  | Formol-ether sediment. | 5/150 | NP | Medium | Ikpe et al. (2022) |
|  | Float (sugar) | 816/1120 | NP | Medium | Ikpeze et al. (2009) |
|  | Float | 86/97 | NP | Medium | Jatau et al. (2011) |
|  | Modified McMaster | 468/1040 | 1,2,13,14,17,18 and 22 | High | Majaro and Dipeolu (1981) |
|  | Direct smear and float (NaCl) | 60/370 | NP | Medium | Nwigwe et al. (2013) |
|  | Float (sugar) and modified McMaster | 69/80 | 1,2,4 and 17 | Medium | Ola-Davies et al. (2002) |
|  | Float (NaCl) | 1,065/1,224 | 1,2,4,7,8 and 18 | High | Woji et al. (1994) |
|  | Float (NaCl) | 168/210 | NP | Medium | Ahmed et al. (1992) |
| Rwanda | Float (NaCl) and McMaster | 124/149 | NP | High | Tumusiime et al. (2022) |
| Senegal | Float and McMaster | 489/577 | 1,2,3,10,12,14,17 and 22 | Medium | Vercruysse (1982) |
| South Africa | Modified McMaster | 384/384 | 1,3,4,5,6,7 and 9 | Medium | Bakunzia et al. (2010) |
|  | Float and modified McMaster | 137/224 | NP | High | Gwaze et al. (2009) |
|  | Float and modified McMaster | 369/369 | NP | High | Harper and Penzhorn (1998) |
|  | Float (NaCl) and modified McMaster | 1,206/1,263 | 1,2,3,4,5,6,7,8,9 and 18 | Medium | Harper and Penzhorn (1999) |
|  | Float (NaCl) and modified McMaster | 173/576 | 1,3,4,5,6,7 and 9 | High | Mpofu et al. (2020) |
| Sudan | Direct smear and float (NaCl) | 135/400 | NP | High | Bakhiet (2020) |
|  | Float | 49/287 | NP | Medium | Koko et al. (2003) |
| Tanzania | Float (NaCl) and McMaster | 357/367 | NP | Medium | Kambarage et al. (1996) |
|  | Float (NaCl) and modified McMaster | 52/81 | NP | Low | Kimbita et al. (2009) |
|  | Float (NaCl) and modified McMaster | 776/995 | 1,2,3,4,6,7,8,9 and 18 | High | Kusiluka et al. (1998) |
|  | Float (NaCl) and modified McMaster | 191/210 | 1,2,3,4,5,6,7 and 9 | Medium | Kusiluka et al. (1996) |
|  | Direct smear, and float | 33/485 | NP | Medium | Mhoma et al. (2011) |
|  | McMaster (NaCl) | 74/129 | NP | Medium | Shija et al. (2014) |
|  | Float and McMaster | 69/131 | 1,3,5,13 and 17 | High | Ngumbi et al. (2015) |
| Zimbabwe | Float (NaCl) and McMaster | 1,124/1,243 | 1,2,3,4,5,6,8,12,13,15,16 and 18 | High | Chhabra and Pandey (1991) |
|  | Float and modified McMaster | 249/580 | NP | High | Zvinorova et al. (2016) |
| **Asia** | | | | | |
| Bangladesh | Float (NaCl) | 107/224 | NP | Medium | Islam and Taimur (2008) |
|  | Float (ZnSo4) | 27/64 | 2,14,17 and 22 | Medium | Mondal and Qadir (1978) |
| China | Float (NaCl, modified McMaster and PCR | 235/245 | 1,2,3,4,5,6,7,8 and 9 | Medium | Liang et al. (2022) |
|  | Float (NaCl) and modified McMaster | 175/199 | 1,3,4,5 and 8 | High | Wang et al. (2010) |
|  | Float (NaCl) and modified McMaster | 568/584 | 1,3,4,5,6 and 7 | High | Zhao et al. (2012) |
| India | Float and Stoll egg counting | 21/275 | NP | Medium | Yadav et al. (2006) |
|  | Float (NaCl) | 141/155 | NP | Medium | Alok et al. (2020) |
|  | Float (NaCl) and McMaster | 149/255 | 1,2,3,4,5, 8 and 15 | Medium | Ashokkumar et al. (2001) |
|  | Float | 95/166 | NP | Medium | Ashraf et al. (2022) |
|  | Float and Stoll egg counting | 209/384 | 1,2,3,5,14,15 and 17 | Medium | Asif et al. (2012) |
|  | Float | 96/943 | NP | Medium | Balasubramaniam et al. (2001) |
|  | Float and Stoll egg counting | 6/115 | NP | Medium | Barua et al. (2009) |
|  | McMaster | 28/46 | NP | Low | Bhattacharya et al. (1996) |
|  | Float and modified McMaster | 995/1155 | 1,14,17 and 18 | Medium | De et al. (2021) |
|  | Float (NaCl) | 1,066/1,200 | NP | Medium | Dibyadarshini and Gupta (2018) |
|  | Float (NaCl) | 1,896/2,441 | NP | Medium | Dixit et al. (2017) |
|  | Float | 497/786 | 1,2,3,5,7,8 and 15 | Medium | Jacob and Pillai (1988) |
|  | Float (NaCl) | 15/15 | NP | Low | Jeyakumar et al. (2009) |
|  | Float, Stoll egg counting and postmortem | 20/80 | NP | Medium | Jeyathilakan (2015) |
|  | Float and modified McMaster | 358/1437 | NP | Medium | Jitendra et al. (2017) |
|  | Direct smear, float and McMaster | 58/60 | 1,2,3,4 and 6 | Medium | Kaur et al. (2019) |
|  | Direct smear and float (NaCl) | 4/50 | NP | Low | Khajuria et al. (2009) |
|  | Float (NaCl) and modified McMaster | 140/250 | NP | Low | Kumar and Balasubramanium (2003) |
|  | Float and modified McMaster | 152/904 | NP | Medium | Laha et al. (2013) |
|  | Float (NaCl) | 222/435 | NP | High | Kumar et al. (2005) |
|  | Microscopic | 60/114 | NP | Medium | Lone et al. (2011) |
|  | Direct smear and float | 180/300 | NP | Low | Maheshwari et al. (2012) |
|  | Direct smear, float (NaCl) and modified McMaster | 145/300 | NP | Medium | Mamatha and Souza (2007) |
|  | Float (NaCl) | 273/1,003 | NP | Medium | Maravi et al. (2022) |
|  | Float and modified McMaster | 55/834 | 1,2,3,5,6,7 and 15 | Medium | Meena et al. (2017) |
|  | Float | 528/2,636 | 1,2,3,6,10,12,18,22 and 23 | Medium | More et al. (2011) |
|  | Microscopic | 26/425 | NP | Medium | Muraleedharan (2005) |
|  | Float and modified McMaster | 261/441 | NP | Medium | Nath et al. (2015) |
|  | NP | 13/500 | NP | Medium | Nayak et al. (2021) |
|  | NP | 18/435 | NP | Low | Padmaja et al. (2007) |
|  | Float (NaCl) | 1,362/1,877 | 1,2,3,4,6 and 17 | Medium | Pandit (2006) |
|  | Qualitative and quantitative | 1,047/1,921 | NP | Medium | Papri et al. (2017) |
|  | Qualitative | 106/301 | 1,2,12,14,17 and 22 | Medium | Patil et al. (1990) |
|  | NP | 11/50 | 1,2,3,4 and 5 | Low | Radha et al. (2004) |
|  | Float and modified McMaster | 2,445/1,2604 | NP | Medium | Rahman et al. (2012) |
|  | Float (NaCl), modified McMaster, and PCR | 949/1,285 | 1,2,3,4,5,6,7,8 and 9 | Medium | Verma et al. (2017) |
|  | Float (NaCl) and modified McMaster | 114/115 | 1,2,3,4,5,6 and 9 | Medium | Verma et al. (2018a) |
|  | Float (NaCl) and modified McMaster | 159/240 | NP | Medium | Saleem et al. (2018) |
|  | Float (sugar) and McMaster | 980/1,545 | 1,2,3,4,5,8,9 and 15 | Medium | Senthilvel et al. (2004) |
|  | Modified McMaster | 672/1,452 | 1, 2,4,5 and 17 | Medium | Sharma and Mandal (2013) |
|  | McMaster | 117/273 | 1,2,3 and 12 | Medium | Sharma et al. (2009) |
|  | McMaster | 999/1,162 | 1,2,3,4, 6 and 9 | Medium | Sharma et al. (2017) |
|  | McMaster | 235/235 | 1,2,3,10,12,17 and 24 | Medium | Sharma et al. (1997) |
|  | Float (NaCl) and modified McMaster | 126/153 | 1,2,3,5 and 6 | Medium | Singh et al. (2016) |
|  | Float and McMaster | 28/212 | NP | Medium | Singh et al. (2017) |
|  | Direct smear, float and McMaster | 32/190 | 1,2,4 and 6 | Medium | Singh et al. (2013) |
|  | Qualitative | 72/360 | NP | Medium | Sorathiya et al. (2017) |
|  | Float (NaCl and McMaster | 129/200 | 1,2,4,5,8 and 22 | Medium | Soundararajan et al. (2019) |
|  | Histopathology | 17/150 | NP | Low | Thangathurai et al. (2003) |
|  | Direct smear | 480/1,520 | NP | Medium | Tiwari et al. (2003) |
|  | Qualitative | 940/1,500 | NP | Medium | Varsha et al. (2010) |
|  | Float (NaCl) | 32/311 | NP | Medium | Verma and Jaiswal (2017) |
|  | Float and modified McMaster | 1,014/1,419 | 1,2,3,4,5,6,7,8 and 9 | Medium | Verma et al. (2018b) |
|  | NP | 285/627 | NP | Low | Gupta et al. (2013) |
|  | Direct smear and float (NaCl) | 20/50 | NP | Low | Bandyopadhyay (1999) |
|  | Float (NaCl) and Stoll egg count | 110/414 | 1 and 2 | Medium | Buragohain et al. (2013) |
|  | Float (NaCl) | 358/450 | 1,2,3,10,13,14,17,18 and 22 | Medium | Chhabra et al. (1983) |
|  | Float (sugar | 219/500 | 1,2,10,12,14,17 and 18 | Medium | Deb et al. (1982) |
|  | Float (NaCl and sugar) | 152/243 | 1,2,3,4,10,12,13,14,17,18,22 and 24 | Medium | Jha and Subramanian (1966) |
|  | Float | 689/1,663 | 1,2,3,5,10,13,14,17,18 and 22 | Medium | Khillare and Narladkar (2014) |
|  | NP | 307/1,639 | 1,2,3,10,13,14,17 and 18 | Medium | Kshirsagar (1981) |
|  | Float (sugar) | 217/500 | 1,2,10,12,14,17 and 18 | Medium | Lodh and Choudhury (1993) |
|  | Float (NaCl) | 100/600 | NP | Medium | Mamta et al. (2013) |
|  | NP | 8/142 | NP | Low | Patel et al. (2001) |
|  | NP | 476/769 | NP | Low | Raote et al. (1987) |
|  | NP | 45/234 | NP | Low | Sapna et al. (2010) |
|  | Float and modified McMaster | 803/960 | NP | Medium | Singh et al. (2014) |
|  | NP | 15/300 | 3 | Medium | Shah (1965) |
|  | Float (sugar) and modified McMaster | 205/205 | 1,2,3,10,13,14,17 and 22 | Medium | Vihan et al. (1988) |
|  | Float (MgSo4) and modified McMaster | 104/130 | NP | Medium | Priyanka et al. (2013) |
|  | Float (sugar) | 114/300 | 1,2,3,10,13,14,17 and 18 | Medium | Shah and Joshi (1963) |
|  | NP | 155/575 | 1,2,3,10,12,13,14,16,17,22 and 23 | Low | Nikam (1999) |
|  | Centrifuge /Sporulation | 237/659 | 1,2,13,14,18 and 22 | Medium | Krishnamurthy and Kshirsagar (1976) |
| Iran | Float (sugar), McMaster and histopathology | 292/350 | 1,2,3,5,6,13,14,17 and 18 | High | Hashemnia et al. (2015) |
|  | Float (NaCl) and modified McMaster and PCR | 64/480 | 1,2,3,5,6,7 and 9 | Medium | Karimzadeh et al. (2022) |
|  | Float (sugar), modified McMaster and histopathology | 187/208 | 1,2,3,5,7,8,17 and 18 | High | Kheirandish et al. (2014) |
|  | Modified McMaster | 391/438 | 1,2,3,14 and 17 | Medium | Radfar et al. (2011) |
| Iraq | Direct smear and float (NaCl) | 29/77 | 2,3,4,5 and 11 | Medium | Al-Bayati et al. (2016) |
|  | Float (sugar) | 142/615 | NP | Medium | Al-Amery and Hasso (2002) |
|  | Direct smear, float and McMaster | 15/18 | 10,12,17 and 25 | Medium | Minnat et al. (2014) |
|  | Float (sugar) | 7/18 | 1,2,3,4,6 and 8 | Medium | Shaheed and Al-Azizz (2020) |
| Jordan | Float (NaCl) and McMaster | 108/200 | 1,2,4,5,6,8,9 and 18 | High | Abo-Shehada and Abo-Farieha (2003) |
| Korea | Float (NaNO3) and modified McMaster | 163/241 | NP | Medium | Gebeyehu et al. (2013) |
|  | Modified McMaster | 314/326 | NP | Low | Bang et al. (1985) |
| Kuwait | Float | 46/222 | NP | High | Abdou et al. (2021) |
|  | Direct smear and float | 2/20 | 1,3 and 6 | Medium | Majeed et al. (2015) |
| Laos | Float and McMaster | 314/434 | NP | High | Windsor et al. (2018) |
| Malaysia | Float (NaCl) | 256/287 | NP | High | Yusof and Isa (2016) |
|  | Float (NaCl) and McMaster | 97/151 | NP | Low | Azima et al. (2020) |
|  | Float (NaCl) and Modified McMaster | 90/197 | NP | High | Azrul et al. (2017a) |
|  | Float and modified McMaster | 99/203 | NP | Medium | Chandrawathani et al. (2009) |
|  | Float (NaCl) | 256/287 | NP | Medium | Hashima and Yusof (2016) |
|  | Float (NaCl) and modified McMaster | 202/227 | NP | Medium | Hisamuddin et al. (2016) |
|  | Float (NaCl) and modified McMaster | 725/815 | 1,2,3,4,5,6,7,9 and 18 | High | Jalila et al. (1998) |
|  | Float and modified McMaster | 11/257 | NP | High | Paul et al. (2020) |
|  | Float (NaCl) and McMaster | 426/480 | NP | High | Nur-Rahifah Inani and Yusof (2018) |
|  | McMaster | 141/218 | NP | Medium | Rozita et al. (2015) |
|  | Float (formal ether) | 99/316 | NP | Medium | Tan et al. (2017) |
| Myanmar | Float (sugar), modified McMaster and nested PCR | 168/280 | 1,3 and 6 | High | Bawm et al. (2020) |
|  | Float and McMaster | 91/100 | NP | Medium | Win et al. (2020) |
| Nepal | Float (NaCl) and modified McMaster | 916/991 | 1,2,3,4,5,6,7,8,9,19,20,21 and 25 | Medium | Ghimire et al. (2021) |
|  | Float (NaCl), direct smear and modified McMaster | 323/400 | NP | Medium | Ghimire and Bhattarai (2019) |
| Pakistan | Float and direct smear | 167/376 | 1,2,4,5 and 6 | Medium | Avais et al. (2021) |
|  | Float and direct smear | 92/252 | NP | Medium | Asif et al. (2008) |
|  | Direct smear and float | 136/310 | NP | Medium | Gadahi et al. (2009) |
|  | Direct smear and float | 43/500 | NP | Medium | Khan et al. (2015) |
|  | Float (NaCl) and McMaster | 271/486 | 1,2,4,5 and 6 | High | Rehman et al. (2012) |
|  | Float | 337/380 | 1,2,3,4,5,7,8,9 and 18 | High | Ahmed (2020) |
|  | McMaster | 67/121 | 1,2,5 and 6 | Medium | Tauseef-ur-Rehman et al (2011) |
|  | Float | 73/260 | 2,13 and 17 | Medium | Hayat et al. (1986) |
| Philippines | NP | 108/173 | 1,11 and 14 | High | Padilla et al. (2009) |
|  | Direct smear | 908/1,127 | 1,11,13 and 14 | Medium | Tongson et al. (1981) |
| Saudi Arabia | Float (sugar) | 206/228 | 1,2,3,4,5,6,7,8,9 and 16 | High | Alyousif et al. (1992) |
|  | Float (NaCl) and McMaster | 6/7 | 1,2 and 5 | Medium | Apio et al. (2013) |
|  | Float (NaCl) | 376/2,689 | NP | High | El-Bahy et al. (2008) |
|  | Float (NaCl) and modified McMaster | 180/336 | 1,2,3,4,5,6,7,8 and 9 | High | Ibrahim (2012) |
|  | Qualitative and quantitative | 26/760 | NP | Medium | Mottelib et al. (1992) |
| Sri Lanka | Float (NaCl) and McMaster | 176/203 | 1,2,3,4,6,7 and 8 | Medium | Faizal and Rajapakse (2001) |
| Thailand | Float (NaCl) and modified McMaster | 76/185 | NP | High | Azrul et al. (2017b) |
|  | Float and direct smear | 6/391 | NP | Medium | Junsiri et al. (2021) |
|  | Formalin ethyl acetate | 155/358 | NP | Medium | Sangvaranond et al. (2010) |
|  | Modified McMaster | 445/885 | NP | High | Wuthijaree et al. (2022) |
| Vietnam | Float (NaCl) and PCR | 218/251 | 1,2,3 and 4 | Medium | Nguyen-Ho-Bao et al. (2022) |

^*^1; *Eimeria ninakohlyakimovae*, 2; *E. arloingi*, 3; *E. christenseni*, 4; *E. alijevi*, 5; *E. caprina*, 6; *E. hirci*,7; *E. jolchijevi*, 8; *E. caprovina*, 9; *E. aspheronica*, 10; *E. cranda1lis*, 11; *E. megaembryonica*, 12; *E. ahsata*, 13; *E. granulosa*, 14; *E. faurei*, 15; *E. kocharli*, 16; *E. punctuia*, 17; *E. parva*, 18; *E. pallida*, 19; *E. tunisiensis*, 20; *E. masseyensi*, 21; *E. capralis*, 22; *E. intricata*, 23; *E. parbhaniensis*, 24; *E.* *hawkin*, 25; *E. charlestoni*, 26; *E. zuernii*. ^1^Quality was categorised as low (1-2), medium (3-4) and high (5-7).

**TABLE S5.** Egger’s test results for publication bias of studies included in this study.

| **Intercept** |  | **Bias** | **Se. bias** | **t** | **df** | **p-value** |
| --- | --- | --- | --- | --- | --- | --- |
| 0.8101 |  | 3.8685 | 2.0610 | 1.88 | 253 | 0.0617 |

**TABLE S6.** Begg’s test results for publication bias of studies (rank correlation test of funnel plot asymmetry) included in this meta-analysis.

| **Ks** | **Se. ks** | **Z** | **p-value** |
| --- | --- | --- | --- |
| -1399.000 | 1361.2683 | -0.103 | 0.3041 |

**TABLE S7.** Egger’s test results for publication bias of studies included in this study based on the continent.

| **Continent** | **Intercept** | **Bias** | **Se. bias** | **t** | **df** | **p-value** |
| --- | --- | --- | --- | --- | --- | --- |
| Asia | 0.6062 | 7.4200 | 2.6416 | 2.81 | 126 | 0.0058 |
| Africa | 1.0817 | -4.8838 | 3.4942 | -1.40 | 70 | 0.1666 |
| Europe | 1.4027 | -5.9389 | 2.4576 | -2.42 | 33 | 0.0215 |
| North America | 1.1602 | 3.7379 | 10.7167 | 0.35 | 8 | 0.7363 |

Note: The number of studies were less than the minimum requirement to perform Egger’s test (*n* = 10) in Oceania and South America

**References**

1. Abdel-Salam, M. and Mahran, O.M., 2004. Some biochemical changes in blood serum of balady goats infested with internal and external parasites at Assiut governorate. *Assiut Veterinary Medical Journal*, *50*(102), pp.145-156.
2. Abdou, N.E., Majeed, Q.A.H., El-Azazy, O.M.E., Tahrani, L.M.A., AlAzemi, M.S. and Alajmi, A., 2021. Risk factors of diarrhea in small ruminants in Kuwait. *Iranian Journal of Veterinary Research*, *22*(2), pp.146-149.
3. Abdurrahman, G., 2007. The Prevalence of Eimeria Species in Goats in Iğdır. *Turkish Journal of Veterinary and Animal Sciences*, *31*(6), pp.411-414.
4. Abebe, W. and Esayas, G., 2001. Survey of ovine and caprine gastro-intestinal helminthosis in eastern part of Ethiopia during the dry season of the year. *Revue de Medecine Veterinaire*,152(5), pp.379-384.
5. Abo-Shehada, M.N. and Abo-Farieha, H.A., 2003. Prevalence of Eimeria species among goats in northern Jordan. *Small Ruminant Research*, *49*(2), pp.109-113.
6. Abou-El-Naga, T.R., 2010. Prevalence of some parasitic diseases causing diarrhoea in small ruminants in northwest coastal area. In *Proceedings of the 3rd animal wealth research conference in the Middle East and North Africa*, pp. 14-24.
7. Adams, J.W. and McKay, J., 1966. Gastrointestinal parasites of farm animals in Eastern Nigeria. *Nature*, *212*(5058), pp.216-217.
8. Adeyemi, M.T., Morenikeji, O.A., Emikpe, B.O. and Jarikre, T.A., 2017. Interactions between gastrointestinal parasitism and pneumonia in Nigerian goats. *Journal of Parasitic Diseases*, *41*, pp.726-733.
9. Adeyemi, O.O., Idowu, E.T., Ikenweiwe, J.C. and Otubanjo, O.A., 2021. Status of Eimeria infections of sheep and goat sold at small ruminant markets in Lagos State, Southwest, Nigeria. *Nigeria Journal of Parasitology*, *42*, pp.137-146.
10. Agyei, A.D., Odonkor, M. and Osei-Somuah, A., 2004. Concurrence of Eimeria and helminth parasitic infections in West African Dwarf kids in Ghana. *Small Ruminant Research*, *51*(1), pp.29-35.
11. Ahmed, M.I., Umoh, J.U. and Brisibe, F., 1992. Risk factors of ovine and caprine coccidiosis in an arid zone of Nigeria. *Indian Veterinary Medical Journal*, *16*, pp.274-278.
12. Ahmed, S., 2020. Parasites of markhor, urial and Chiltan wild goat in Pakistan. *Annals of Parasitology*, *66*(1), pp.3-12.
13. Al-Amery, M.A.Y. and Hasso, S.A., 2002. Laboratory diagnosis of novel species of Theileria hirci, Eimeria caprovina and Eimeria pallida in goats in Iraq. *Small Ruminant Research*, *44*(2), pp.163-166.
14. Al-Bayati, S.M., Al-Rekani, A.M. and Hamed, A.A., 2016. Diagnosis of Eimeria spp. in Capra ibex (local meriz goat). *The Iraqi Journal of Veterinary Medicine*, *40*(1), pp.47-52.
15. Al-Habsi, K., Yang, R., Ryan, U., Miller, D.W. and Jacobson, C., 2017. Morphological and molecular characterization of three Eimeria species from captured rangeland goats in Western Australia. *Veterinary Parasitology: Regional Studies and Reports*, *9*, pp.75-83.
16. Alok, K.S., Daya, S., Pramod, K.R., Ashok, K. and Pradeep, K., 2020. Studies on Eimeria species in goats of Mathura region, Uttar Pradesh, India. *Haryana Veterinarian*, *59*(1), pp.131-132.
17. Alyousif, M.S., Kasim, A.A. and Al-Shawa, Y.R., 1992. Coccidia of the domestic goat (Capra hircus) in Saudi Arabia. *International journal for parasitology*, *22*(6), pp.807-811.
18. Anderson, D.L. and Roberson, E.L., 1996. Gastrointestinal and respiratory parasitism in Georgia goats. *Agri-Practice (USA)*.
19. Anene, B.M., Onyekwodiri, E.O., Chime, A.B. and Anika, S.M., 1994. Gastrointestinal parasites in sheep and goats of southeastern Nigeria. *Small ruminant research*, *13*(2), pp.187-192.
20. Apio, A., Mohammed, O.B., Omer, S.A. and Wronski, T., 2013. Cross infection with gastro-intestinal tract parasites between domestic goat and endemic Farasan gazelle (Gazella gazella farasani) in Farasan Islands, Saudi Arabia. *Journal of King Saud University-Science*, *25*(4), pp.325-329.
21. Arafa, M.I., 2002. Prevalence studies on coccidia of goats in Assiut Governorate with notes on seasonal variation. *Assiut Veterinary Medical Journal*, *46*(92), pp.213-223.
22. Ashokkumar, M., Elayaraja, M., Anna, T. and Sundar, T., 2001. Species spectrum of Eimeria in goats of Namakkal area, Tamil Nadu. *Journal of Veterinary Parasitology*, *15*, pp.39-41.
23. Ashraf, A., Tramboo, S.R., Maqbool, I., Allaie, I.M., Bulbul, K.H., Shahardar, R.A., Wani, Z.A. and Sheikh, F.D., 2022. Occurrence of GI parasites in ruminants of Kashmir and Ladakh. *Journal of Parasitic Diseases*, *46*(1), pp.196-201.
24. Ashraf, M. and Nepote, K.H., 1990. Prevalence of gastrointestinal nematodes, coccidia and lungworms in Maryland dairy goats. *Small Ruminant Research*, *3*(3), pp.291-298.
25. Asif, I., Wazir, V.S., Singh, R., Malik, M.A., Manzoor, A. and Reyaz, C., 2012. Coccidiosis in goats of organized and unorganized farms of Jammu Region. *Indian Journal of Field Veterinarians*, *7*(4), pp.32-34.
26. Asif, M., Azeem, S., Asif, S. and Nazir, S., 2008. Prevalence of gastrointestinal parasites of sheep and goats in and around Rawalpindi and Islamabad, Pakistan. Journal of Veterinary and Animal Sciences, *1*(1), pp.14-17.
27. Avais, M., Rashid, G., Awais, M., Zaman, S.H., Khan, M.A., Ahmad, S.S. and Amjad, S., 2021. Prevalence and Associated Risk Factors of Coccidiosis in Small Ruminants in Dera Ghazi Khan, Punjab, Pakistan. *Lahore Garrison University Journal of Life Sciences*, *5*(4), pp.270-281.
28. Ayana, D., Tilahun, G. and Wossene, A., 2009. Study on Eimeria and Cryptosporidium infections in sheep and goats at Elfora export abattoir, Debre-Zeit, Ethiopia. *Turkish Journal of Veterinary and Animal Sciences*, *33*(5), pp.367-371.
29. Azima, L.H., Azizah, D., Nurulaini, R., Adnan, M., Lily Rozita, M.H., Debbra, M., Erwanas, A.I. and Chee, W.K., 2020. Occurrence of parasitic infection in small ruminants from various farms in Perak, Malaysia. *Malaysian Journal of Veterinary Research*, *11*(1), pp.18-25.
30. Azrul, L.M., Noor, C.R.M., Poungpong, K., Prasanpanich, S. and Jittapalapong, S., 2017a. Prevalence and Characterization of Gastrointestinal Parasites Infection in Goats from Small-Scale Farms in Northern Part of Terengganu State, Peninsular Malaysia. *Journal of Science and Technology in the Tropics*, 13(2), pp.54-62.
31. Azrul, L.M., Poungpong, K., Jittapalapong, S. and Prasanpanich, S., 2017b. Descriptive prevalence of gastrointestinal parasites in goats from small farms in Bangkok and vicinity and the associated risk factors. *Annual Research and Review in Biology*, 16(2), pp.1-7.
32. Bakhiet, A.E., 2020. Prevalence and Risk Factors of Caprine Coccidiosis in Atbara Locality-River Nile State. *Msc* *Thesis,* Sudan University of Science and Technology (SUST).
33. Bakunzi, F.R., Thwane, S.N., Motsei, L.E. and Dzoma, B.M., 2010. Diversity and seasonal occurrence of Eimeria species in a mixed flock of communally reared sheep and goats in Mafikeng in the Northwest Province, South Africa. *Journal of the South African veterinary association*, *81*(3), pp.148-150.
34. Balasubramaniam, G.A., Rao, G.V., Balachandran, C., George, V.T. and Airamuthu, S.V., 2001. Incidence of parasitic diseases among domestic animals in Namakkal district of Tamil Nadu. *Indian Journal of Animal Sciences,71(4),* pp.340-341
35. Balicka-Ramisz, A., Ramisz, A., Vovk, S. and Snitynskyj, V., 2012. Prevalence of coccidia infection in goats in Western Pomerania (Poland) and West Ukraine region. *Annals of Parasitology*, *58*(3), pp.167-171.
36. Balicka-Ramisz, A., 1999. Studies on coccidiosis in goats in Poland. *Veterinary Parasitology*, *81*(4), pp.347-349.
37. Bandyopadhyay, B., 1999. Gastrointestinal parasitic infections of sheep and goats at Salboni, West Bengal. *Journal of Veterinary Parasitology*, *13*(1), pp.79-80.
38. Bang, K. S., Kim, D. J., Kim, Y. K. and Choi, K.M., 1985. Significance of coccidial infection in Korean native goats. Efficient animal production for Asian welfare. Proceedings of the 3rd AAAP Animal Science Congress, Seoul, Korea, 1, pp.570-572.
39. Barua, C.C., Chamuah, J.K., Barua, A.G., Saleq, A., Hazarika, A. and Biswas, R., 2009. Prevalence of gastrointestinal parasites in ruminants. *Indian Veterinary Journal*, *86*(7), pp.747-748.
40. Bawm, S., Win, T.Z.B., Win, S.Y., Htun, L.L., Nakao, R. and Katakura, K., 2020. First detection of Eimeria species in Myanmar domestic goats with both microscopic and molecular methods. *Parasite*, *27*(38).
41. Bersissa, K., Tigist, T., Teshale, S., Reta, D. and Bedru, H., 2011. Helminths of sheep and goats in central Oromia (Ethiopia) during the dry season. *Journal of Animal and Veterinary advances*, *10*(14), pp.1845-1849.
42. Bhattacharya, D., Mahesh, C. and Laha, R., 1996. Parasites of livestock in central Himalayan region. *Indian Journal of Veterinary Research*,*5*, pp.51-53.
43. Borgsteede, F.H.M. and Dercksen, D.P., 1996. Coccidial and helminth infections in goats kept indoors in the Netherlands. *Veterinary Parasitology*, *61*(3-4), pp.321-326.
44. Buragohain, B., Phukan, A., Dutta, T.C., Barua, C.C. and Phukan, S.C., 2013. Prevalence of coccidiosis in kids in and around Guwahati. *Journal of Veterinary Parasitology*, *27*(1), pp.25-28.
45. Cantú-Martínez, M. A., González-Sáenz, I. S., Pereira-Berto, B., Zamora-Ávila, D. E., Ávalos-Ramírez, R., Vázquez-Cisneros, K. W., Mar-Aguilar, F. and Zarate-Ramos, J. J., 2022. Identification of Eimeria species present in goats (Capra aegagrus hircus) in Nuevo León, Mexico, *Journal MVZ Cordoba*, 27, pp. e2560.
46. Cardoso, C.P., Cardozo, L.L., Silva, B.F.D. and Amarante, A.F.T.D., 2012. Gastrointestinal parasites in goats from Monte Castelo, Santa Catarina, Brazil. *Revista Brasileira de Parasitologia Veterinária*, *21(2)*, pp.148-150.
47. Carrau, T., Garijo, M.M., Martínez-Carrasco, C., Pérez, D., Silva, L.M.R., Taubert, A., Hermocilla, C. and De Ybáñez, R.R., 2017. Parasite prevalence in Mycobacterium spp. infected dairy goats in the region of Murcia (south-east Spain). *Revue scientifique et technique*, *36*(3), pp. 905-916.
48. Cavalcante, A.C.R., Teixeira, M., Monteiro, J.P. and Lopes, C.W.G., 2012. Eimeria species in dairy goats in Brazil. *Veterinary parasitology*, *183*(3/4), pp.356-358.
49. Celi, K., Guzmán, L. and Rey-Valeirón, C., 2022. Apicomplexans in Goat: Prevalence of Neospora caninum, Toxoplasma gondii, Cryptosporidium spp., Eimeria spp. and Risk Factors in Farms from Ecuador. *Animals*, *12*(17), pp.2224.
50. Chandrawathani, P., Nurulaini, R., Adnan, M., Premalaatha, B., Khadijah, S., Jamnah, O., Zaini, C.M., Khor, S.K. and Zawida, Z., 2009. A survey of parasitic infection on small ruminant farms in Kinta and Hilir Perak districts, Perak, Malaysia. *Tropical Biomedicine*, *26*(1), pp.11-15.
51. Chhabra, R.C. and Pandey, V.S., 1991. Coccidia of goats in Zimbabwe. *Veterinary Parasitology*, *39*(3/4), pp.199-205.
52. Chhabra, M.B., Bhatnagar, P.K. and Singh, R.P., 1983. Prevalence of coccidia of goats in Haryana. *Indian Veterinary Medical Journal*, 7(1), pp.13-16.
53. Chikweto, A., Tiwari, K., Bhaiyat, M.I., Carloni, J., Pashaian, K., Pashaian, A., De Allie, C. and Sharma, R.N., 2018. Gastrointestinal parasites in small ruminants from Grenada, West Indies: A coprological survey and a review of necropsy cases. *Veterinary Parasitology: Regional Studies and Reports*, *13*, pp.130-134.
54. Chohan, H. 2022. Investigating the infectious causes of a digestive syndrome in dairy goats. *Honors Thesis. The* University of Melbourne*, Australia.*
55. Coelho, W.M.D., Amarante, A.F.T.D. and Bresciani, K.D.S., 2012. Occurrence of gastrointestinal parasites in goat kids. *Revista Brasileira de Parasitologia Veterinária*, *21(1)*, pp.65-67.
56. Corrias, F., Brajon, G., Salari, F.E.D.E.R.I.C.A., Dal Prà, A., Ragona, G., Lombardo, A., Mari, M., Altomonte, I., Colombani, G., Pedri, P. and Scotti, B., 2012. Health evaluation in the native Garfagnina goat. *Small Ruminant Research*, *104*(1/3), pp.191-194.
57. Das, G., Tölü, C., Konyalı, A., Brka, M. and Savaș, T., 2006. A study of the course of Eimeria spp. oocyst output of naturally infected kids. Radovi Poljoprivrednog Fakulteta Univerziteta u Sarajevu (Works of the Faculty of Agriculture University of Sarajevo), 51(57(3)), pp. 65-71.
58. De, A.K., Perumal, P., Muniswamy, K., Ahmed, S.Z., Kundu, A., Sunder, J., Alyethoda, R., Ravi, S.K. and Bhattacharya, D., 2021. Prevalence of coccidiosis in Andaman local goat and its metaphylaxis in tropical island ecosystem. *Indian Journal of Animal Science,*91(6), pp. 438-442.
59. De la Fuente, C. and Alunda, J.M., 1992. A quantitative study of Eimeria infections of goats from central Spain. *Veterinary Parasitology*, *41*(1-2), pp.7-15.
60. Deb, A.R., Ansari, M.Z., Sinha, B.N. and Sahai, B.N., 1982. Incidence and biology of Eimerian species in goats at Ranchi, Chotanagpur, India. *Indian Journal of Animal Health*, 21(1), pp.33-38.
61. Debele, G., Duguma, M., Hundessa, F., Messele, F., Kebede, T. and Negash, M., 2013. Study on major causes of kid mortality in Adami Tulu Jido Kombolcha District of Oromia, Ethiopia. *Agriculture and Biology Journal of North America*, *4*(2), pp.110-115.
62. Deger, S., Gul, A., Ayaz, E. and Bicek, K., 2003. The prevalence of Eimeria species in goats in Van. *Turkish Journal of Veterinary and Animal Sciences*, *27*(2), pp.439-442.
63. de Macedo, L.O., Santos, M.A.B., da Silva, N.M.M., do Rêgo Barros, G.M.M., Alves, L.C., Giannelli, A., Ramos, R.A.N. and de Carvalho, G.A., 2019. Morphological and epidemiological data on Eimeria species infecting small ruminants in Brazil. *Small Ruminant Research*, *171*, pp.37-41.
64. Di Cerbo, A.R., Manfredi, M.T., Zanzani, S. and Stradiotto, K., 2010. Gastrointestinal infection in goat farms in Lombardy (Northern Italy): Analysis on community and spatial distribution of parasites. *Small Ruminant Research*, *88* (2/3), pp.102-112.
65. Dibyadarshini, M.R. and Gupta, N., 2018. Study on Coccidiosis in Goats in Durg District. *Indian Veterinary Journal*, *95*(5), pp.16-17.
66. Dimitrijević, S., Ilić, T., Ðurić, B. and Bojkovski, J., 2006. Parasitic infections in ruminants in some epizootiological areas in the Republic of Serbia. *Slovenian Veterinary Research*, *43*, pp.285-288.
67. Dixit, A.K., Das, G. and Baghel, R.P.S., 2017. Epidemiology of coccidial infections in goats in and around Jabalpur, India. *Environment and Ecology*, *35*(1B), pp.431-433.
68. Drew, M.L. and Weiser, G.C., 2017. Potential disease agents in domestic goats and relevance to bighorn sheep (Ovis canadensis) management. *PLOS ONE*, *12*(3).
69. El-Bahy, M.M., Omer, O.H. and Al-Sadrani, A.A., 2008. Temperature difference and parasite infection at Qassim region, Saudi Arabia. *Research Journal of Parasitology*, *3*(4), pp.114-1221.
70. El-Manyawe, S.M., Abdel Rahman, M.A.M., Abd El Aal, A.M.I., Kamal, A.M. and Snousi, S.A., 2010. Prevalence of some protozoa and its effects on biochemical changes in goats in Cairo, Marsa Matrouh, and El-Wadi El-Gadid provinces. *Egyptain Journal of Comparative Pathology and Clinical Pathology*, *23*, pp.102-115.
71. El-Manyawe, S.M.,1999. Intestinal protozoal infection among goats in Egypt. *Veterinary Medical Journal Giza*, *47*(*3*)*,* pp.379-388.
72. El-Shahawy, I.S., 2016. Coproscopic study on enteric protozoan parasites of goats (Capra hircus L., 1758) in Upper Egypt. *Pakistan Journal of Zoology*, *48*(5), pp.1477-1483.
73. Etsay, K., Megbey, S. and Yohannes, H., 2020. Prevalence of sheep and goat coccidiosis in different districts of Tigray region, Ethiopia. *Nigerian Journal of Animal Science*, *22*(3), pp.61-69.
74. Faizal, A.C.M. and Rajapakse, R.P.V.J., 2001. Prevalence of coccidia and gastrointestinal nematode infections in cross bred goats in the dry areas of Sri Lanka. *Small Ruminant Research*, *40*(3), pp.233-238.
75. Gadahi, J.A., Arshed, M.J., Ali, Q., Javaid, S.B. and Shah, S.I., 2009. Prevalence of gastrointestinal parasites of sheep and goat in and around Rawalpindi and Islamabad, Pakistan. *Veterinary World*, *2*(2), pp.51-53.
76. García-Dios, D., Panadero, R., Díaz, P., Viña, M., Remesar, S., Prieto, A., López-Lorenzo, G., Martínez-Calabuig, N., Díez-Baños, P., Morrondo, P. and López, C.M., 2021. The goat as a risk factor for parasitic infections in ovine flocks. *Animals*, *11*(7), pp.2077.
77. Gebeyehu, E.B., Seo, M.G., Jung, B.Y., Byun, J.W., Oem, J.G., Kim, H.Y. and Kwak, D., 2013. Prevalence of gastrointestinal parasites in Korean native goats (Capra hircus aegagrus). *The Journal of Animal and Plant Science*, *23*(4), pp.986-989.
78. Ghanem, M.M., Mohamed, A.D. and Ramadan, M.Y., 2009. Clinical, biochemical, and histopathological study on parasitic gastroenteritis associated with caprine coccidiosis: comparative effect of Toltrazuril and Propolis. *Lucrări Științifice-Medicină Veterinară, Universitatea de Științe Agricole și Medicină Veterinară" Ion Ionescu de la Brad" Iași*, *52*(11 (1)), pp.565-580.
79. Ghimire, T.R. and Bhattarai, N., 2019. A survey of gastrointestinal parasites of goats in a goat market in Kathmandu, Nepal. *Journal of Parasitic Diseases*, *43*(4), pp.686-695.
80. Ghimire, T.R., Adhikari, R.B. and Bhattarai, N., 2021. Diversity and prevalence of Eimeria species in goats of Nepal. *Journal of the Hellenic Veterinary Medical Society*, *72*(4), pp.3299-3306.
81. Githigia, S.M., Munyua, W.K., Kyvsgaard, N.C. and Thamsborg, S.M., 1998. Helminth infection levels in goats in a semi-arid area of Kenya. *Bulletin of animal health and production in Africa*, *46(3)*, pp.209-210.
82. Githigia, S., 1990. Caprine coccidiosis: epidemiological studies in selected areas of Kenya and aspects of pathology. *PhD dissertation*, University of Nairobi, Kenya.
83. Gorski, P., Niznikowski, R., Strzelec, E., Popielarczyk, D., Gajewska, A. and Wedrychowicz, H., 2004. Prevalence of protozoan and helminth internal parasite infections in goat and sheep flocks in Poland. *Archiv Fur Tierzucht*, *47*(6), pp.43-49.
84. Göz, Y., Aydın, A., Yüksek, L. and Değer, S., 2006. Frequency of coccidia species in goats in Van province of Turkey. *Journal of Kafkas University Faculty of Veterinary Medicine*, *12*(*2*), pp.163-165.
85. Gul, A., Aydİn, A. and Goz, Y., 2008. Prevalence of Eimeria species in goats*. Indian Veterinary Journal*, 85(5), pp.564-565.
86. Gupta, A., Dixit, A.K., Dixit, P. and Mahajan, C., 2013. Prevalence of gastrointestinal parasites in small ruminants in and around Jabalpur, India. *Journal of Veterinary Parasitology*, *27*(1), pp.59-63.
87. Gwaze, F.R., Chimonyo, M. and Dzama, K., 2009. Prevalence and loads of gastrointestinal parasites of goats in the communal areas of the Eastern Cape Province of South Africa. *Small Ruminant Research*, *84*(1/3), pp.132-134.
88. Harper, C.K. and Penzhorn, B.L., 1998. Seasonal occurrence of coccidia in a mixed herd of sheep and goats at Nebo, Northern Province, South Africa: research communication. *Journal of the South African Veterinary Association*, *69*(3), pp.93-94.
89. Harper, C.K. and Penzhorn, B.L., 1999. Occurrence and diversity of coccidia in indigenous, Saanen and crossbred goats in South Africa. *Veterinary Parasitology*, *82*(1), pp.1-9.
90. Hashemnia, M., Rezaei, F. and Chalechale, A., 2015. Prevalence, intensity, and pathological lesions of Eimeria infection in goats in western Iran. *Comparative Clinical Pathology*, *24(4)*, pp.805-810.
91. Hashim, N. and Yusof, A.M., 2016. Rearing systems related to gastrointestinal parasites in goats from selected area in Terengganu. *Technology Journal (Sciences and Engineering)*, 78(*10)*, pp.133-138.
92. Hassan, N.M., Farag, T.K., Abu El Ezz, N.M. and Abou-Zeina, H.A., 2019. Prevalence assessment of gastrointestinal parasitic infections among goats in Giza Governorate, Egypt. *Bulletin of the National Research Centre*, *43*(1), pp.1-7.
93. Hassanen, E.A., Anter, R.G., El-Neshwy, W.M. and Elsohaby, I., 2020. Prevalence and Phylogenetic Analysis of Eimeria Species in Sheep and Goats in Sharkia Governorate, Egypt. *Pakistan Veterinary Journal*, *40*(4), pp.437-442.
94. Hassanen, E., Gouda, A. and Anter, R., 2020. Prevalence and associated risk factors of gastrointestinal parasitic infection in goats in sharkia governorate, Egypt. *Journal of Experimental Biology and Agricultural Sciences*, *8(4)*, pp.524-530.
95. Hayat, C.S., Iqbal, Z. and Malik, A.A., 1986. Prevalence of coccidiosis in sheep and goats at Faisalabad (Pakistan). *Pakistan Veterinary Journal (Pakistan)*, *6*(*4*), pp.198-199.
96. Hisamuddin, N.H., Hashim, N., Soffian, S.N., Rahman, R.N.R.I.R., Mohammad, M., Isa, M.L.M. and Yusof, A.M., 2016. The identification of helminths and coccidia infection from goats in two farms in Kuala Terengganu, Malaysia. *Technology Journal (Sciences and Engineering)*, *78*(9), pp.9-12.
97. Holm, S.A., Sörensen, C.R., Thamsborg, S.M. and Enemark, H.L., 2014. Gastrointestinal nematodes and anthelmintic resistance in Danish goat herds. *Parasite*, *21*, pp 37.
98. Iacob, O.C., 2012. Dynamics of digestive and pulmonary parasitic elements in Carpathian goats, at the end of stabulation. *Lucrări Științifice-Medicină Veterinară, Universitatea de Științe Agricole și Medicină Veterinară" Ion Ionescu de la Brad" Iași*, *55*(3/4), pp.712-719.
99. Ibrahim, M.M., 2012. Prevalence of Eimeria species of the domestic goats Capra hircus Linnaeus, 1758 in Al-Baha area, Saudi Arabia. *Egyptian Academic Journal of Biological Sciences, B. Zoology*, *4*(1), pp.165-172.
100. Ikpe, R.T., Agbendeh, N.L. and Akinsuyi, O.S., 2022. Prevalence study of zoonotic gastrointestinal parasitic infections in goats in Makurdi Metropolis, Nigeria. *Journal of Zoonotic Diseases*, *6*(1), pp.11-16.
101. Ikpeze, O.O., Eneanya, C.I. and Ikerionwu, P.N., 2009. Prevalence Of Coccidiosis in West African Dwarf (Wad) Goats at Mgbakwu, Anambra State, South-Eastern Nigeria. *Zoologist*, *7*, pp.162-167.
102. Islam, K.B.M.S. and Taimur, M.J.F.A., 2008. Helminthic and protozoan internal parasitic infections in free ranging small ruminants of Bangladesh. *Slovenian Veterinary Research*, *45*(2), pp.67-72.
103. Jacob, L. and Pillai, K.M., 1988. Prevalence of Eimeria species in domestic goats in Kerala. *Kerala Journal of Veterinary Science*, *19*(2), pp.45-48.
104. Jalila, A., Dorny, P., Sani, R., Salim, N.B. and Vercruysse, J., 1998. Coccidial infections of goats in Selangor, peninsular Malaysia. *Veterinary Parasitology*, *74*(2-4), pp.165-172.
105. Jaramillo, A., Guzmán, L., Castillo, L., Saa, L.R. and Rey-Valeirón, C., 2017. Gastrointestinal parasitism and usefulness of FAMACHA© in goats from Loja Province, Southern Ecuador. *Revista Científica*, *27*(3), pp.180-186.
106. Jatau, I.D., Abdulganiyu, A., Lawal, A.I., Okubanjo, O.O. and Yusuf, K., 2011. Gastrointestinal and haemo parasitism of sheep and goats at slaughter in Kano, northern-Nigeria. *Sokoto Journal of Veterinary Sciences*, *9*(1), pp.7-11.
107. Jeyakumar, S., Kumar, B.G., Kuntola, R., Jai, S. and Kundu, A., 2009. Incidence of parasitic infection in livestock and poultry in Andaman. *Indian Veterinary Journal*, *86*(11), pp.1178-1179.
108. Jeyathilakan, N., 2015. Status of parasitic infections in small ruminants at an organized farm in semiarid region of Tamil Nadu. *Journal of Veterinary Parasitology*, *29*(1), pp.27-29.
109. Jha, D. and Subramanian, G., 1966. Incidence of Eimeria species in goats of Uttar Pradesh. *The Indian Veterinary Journal*, *43*(7), pp.588-591.
110. Jitendra, T., Daya, S., Sharma, D.K. and Souvik, P., 2017. Epidemiological studies for gastrointestinal parasitic diseases in a pure breed Jamunapari herd. *Environment and Ecology*, *35*(3B), pp.2091-2094.
111. Junsiri, W., Tapo, P., Chawengkirttikul, R., Watthanadirek, A., Poolsawat, N., Minsakorn, S. and Anuracpreeda, P., 2021. The occurrence of gastrointestinal parasitic infections of goats in Ratchaburi, Thailand. *The Thai Journal of Veterinary Medicine*, *51*(1), pp.151-160.
112. Juszczak, M., Sadowska, N. and Udala, J., 2019. Parasites of the digestive tract of sheep and goats from organic farms in Western Pomerania, Poland. *Annals of Parasitology*, *65*(3), pp.245-250.
113. Kahan, T.B. and Greiner, E.C., 2013. Coccidiosis of goats in Florida, USA. *Open Journal of Veterinary Medicine*, *3*(*3*), pp.209-212*.*
114. Kambarage, D.M., Kimera, S.I., Kusiluka, L.J.M. and Mtambo, M.M.A., 1996. Prevalence of Eimeria and Cryptosporidium oocysts in cattle, sheep, and goats in Morogoro region, Tanzania. *Journal of Applied Animal Research*, *9*(1), pp.73-78.
115. Kanyari, P.W.N., Kagira, J.M. and Mhoma, R.J., 2009. Prevalence and intensity of endoparasites in small ruminants kept by farmers in Kisumu Municipality, Kenya. Livestock Research for Rural Development,21(11), pp, 202.
116. Kanyari, P.W., 1988. Coccidiosis in goats and aspects of epidemiology. *Australian veterinary journal*, *65*(8), pp.257-258.
117. Karimzadeh, M., Kojouri, G., Azizi, H., Pirali, Y. and Shiran, B., 2022. Small Ruminants Coccidiosis in High Altitude Region of Iran. *Asian Research Journal of Agriculture*, *15*(4), pp.116-123.
118. Kaur, S., Singla, L.D., Sandhu, B.S., Bal, M.S. and Kaur, P., 2019. Coccidiosis in goats: pathological observations on intestinal developmental stages and anticoccidial efficacy of amprolim. *Indian Journal of Animal Research*, *53*(2), pp.245-249.
119. Khajuria, J.K., Yadav, A., Vohra, S., Katoch, R., Sood, S., Singh, A. and Agrwal, R., 2009. An outbreak of parasitic gastroenteritis in sheep and goats in RS Pura, Jammu. *Veterinary Practioner*, *10*, pp.71-72.
120. Khan, N.U., Shafee, M., Khan, J.A., Ashraf, K., Khan, R., Khan, A. and Khan, M.A., 2015. Sero-epidemiological study of ecto-and endo-parasites and their hematological effects in small ruminants reared under pastoral system in district Bannu, Pakistan. *Pure and Applied Biology (PAB)*, *4*(*1*), pp.50-56.
121. Kheirandish, R., Nourollahi-Fard, S.R. and Yadegari, Z., 2014. Prevalence and pathology of coccidiosis in goats in southeastern Iran. *Journal of parasitic diseases*, *38(1)*, pp.27-31.
122. Khillare, B.S. and Narladkar, B.W., 2014. Epidemiology of coccidiosis in caprines of Marathwada region of Maharashtra a) Age, sex, breed and season wise prevalence. *Journal of Veterinary Parasitology*, *28*(1), pp.7-13.
123. Kiltu, G., Keffale, M. and Muktar, Y., 2016. Study on Prevalence of Small Ruminant Coccidiosis in and Around Harmaya, Eastern Haraghe Ethiopia. *Acta Parasitologica Globalis*, *7*(1), pp.7-11.
124. Kimbita, E.N., Silayo, R.S., Mwega, E.D., Mtau, A.T. and Mroso, J.B., 2009. Studies on the Eimeria of goats at Magadu dairy farm SUA, Morogoro, Tanzania. *Tropical animal health and production*, *41*, pp.1263-1265.
125. Koinari, M., Karl, S., Ryan, U. and Lymbery, A.J., 2013. Infection levels of gastrointestinal parasites in sheep and goats in Papua New Guinea. *Journal of helminthology*, *87*(4), pp.409-415
126. Koko, W., Gala, M. and Abdalla, H., 2003. Gastrointestinal parasites of the Gezira goats: Central Sudan. *Journal of Animal and Veterinary Advances*, *2(3)*, pp.392-395.
127. Koudela, B. and Boková, A., 1998. Coccidiosis in goats in the Czech Republic. *Veterinary Parasitology*, *76*(4), pp.261-267.
128. Krishnamurthy, R. and Kshirsagar, H.S., 1976. Incidence of coccidia in goats of Marathwada region (Maharashtra). *Maralhwada University Journal of Science (National Sciences)*, *15(8)*, pp.153-156.
129. Kshirsagar, H.S., 1981. Note on Prevalence, seasonal variation, and species composition of Eimeria occurring in goats in Marathwada region. *Indian Journal of Animal Sciences*, *51*(*5*), pp.572-575.
130. Kumar, M.J.M. and Balasubramanium, G.A., 2003. Incidence of coccidiosis in goats in Namakkal. *Indian Veteinary Journal*, 80 (8), pp. 817-818.
131. Kumar, L., Sinha, S.R.P., Sinha, S., Sharma, S.K., Mandal, K.G. and Verma, S.B., 2005. Studies on Eimeria Sp. in goats in and around Patna. *Journal of Veterinary Parasitology*, *19* (2), pp.139-141.
132. Kusiluka, L.J.M., Kambarage, D.M., Matthewman, R.W., Harrison, L.J.S. and Daborn, C.J., 1996. Coccidiosis of small ruminants in Tanzania. *Small Ruminant Research*, *21*(2), pp.127-131.
133. Kusiluka, L.J.M., Kambarage, D.M., Harrison, L.J.S., Daborn, C.J. and Matthewman, R.W., 1998. Prevalence and seasonal patterns of coccidial infections in goats in two ecoclimatic areas in Morogoro, Tanzania. *Small Ruminant Research*, *30*(2), pp.85-91.
134. Kyriánová, I.A., Vadlejch, J., Kopecký, O. and Langrová, I., 2017. Seasonal dynamics of endoparasitic infections at an organic goat farm and the impact of detected infections on milk production. *Parasitology Research*, *116(11)*, pp.3211-3219.
135. Kyriánová, I.A., Kopecký, O., Šlosárková, S. and Vadlejch, J., 2019. Comparison of internal parasitic fauna in dairy goats at conventional and organic farms in the Czech Republic. *Small Ruminant Research*, *175*, pp.126-132.
136. Laha, R., Das, M. and Goswami, A., 2013. Seasonal dynamics of gastrointestinal parasitic infections and prediction of Haemonchus contortus infections of goats in a subtropical hilly region of north-eastern India. *Indian Journal of Animal Research*, *47*(4), pp.309-314.
137. Liang, G., Yang, X., Liu, D., Li, Y., Wang, J., Chen, X., Zhao, G. and Song, J., 2022. Molecular Characterization of 18S rDNA, ITS-1, ITS-2, and COI from Eimeria christenseni and E. arloingi in Goats from Shaanxi Province, Northwestern China. *Animals*, *12*(11), pp.1340.
138. Lianou, D.T., Arsenopoulos, K.V., Michael, C.K., Papadopoulos, E. and Fthenakis, G.C., 2022. Protozoan Parasites in Adult Dairy Small Ruminants and Potential Predictors for Their Presence in Faecal Samples. *Microorganisms*, *10*(*10*), pp.1931.
139. Lima, J.D., 1980. Prevalence of coccidia in domestic goats from Illinois, Indiana, Missouri, and Wisconsin. *International Goat and Sheep Research*, *1*(*3*), pp.234-241.
140. Lloyd, S. and Soulsby, E.J., 1978. Survey of parasites in dairy goats. *American Journal of veterinary research*, *39*(6), pp.1057-1059.
141. Lodh, C. and Choudhury, M.N., 1993. Incidence and intensity of goat coccidiosis in west Bengal. *Environment and Ecology*, *11*, pp.115-115.
142. Lone, B.A., Chishti, M.Z. and Fayaz, A., 2011. Prevalence of coccidia and gastrointestinal nematode infections in goats of Barramulla District of Kashmir Valley. *Global Veterinaria*, *7*(1), pp.27-30.
143. Magi, M., Campo, M., Malloggi, M., Sbrana, L. and Casarosa, L.,1986. Eimeria species of goats in Italy*. Annals of the Faculty of Veterinary Medicine of Pisa, 3*9, pp.185-188.
144. Maheshwari, A., Tanwar, R.K., Chahar, A. and Singh, A.P., 2012. Prevalence of brucellosis and parasitic infestation in goats in Bikaner. *Veterinary Practitioner*, *13*(2), pp.333-334.
145. Mahmoud, H., Ali, A. and Hamada Ali, R.A.N.I.A., 2018. Evaluation of clinical status and treatment trials in sheep and goats infested with Eimeria species. *Assiut veterinary medical journal*, *64*(156), pp.123-128.
146. Maichomo, M.W., Kagira, J.M. and Walker, T., 2004. The point prevalence of gastro-intestinal parasites in calves, sheep, and goats in Magadi division, south-western Kenya. *Onderstepoort Journal of Veterinary Research*, *71*(4), pp.257-261.
147. Maingi, N. and Gichohi, V.M., 1993. Prevalence of gastrointestinal helminths and coccidial parasites and frequency distribution of some nematode genera of goats on some farms in four districts of Kenya. *Bulletin of Animal Health and Production in Africa*
148. Majaro, O.M. and Dipeolu, O.O., 1981. The seasonal incidence of coccidia infections in trade cattle, sheep, and goats in Nigeria. *Veterinary Quarterly*, *3*(2), pp.85-90.
149. Majeed, Q.A., Alazemi, M.S., Henedi, A.A. and Tehrani, L., 2015. Study on parasites from farm animals in Kuwait. *Journal of the Egyptian Society of Parasitology*, *45*(1), pp.71-74.
150. Mamatha, G.S. and D’Souza, P.E., 2007. Gastrointestinal parasitism in sheep and goats from different districts of Karnataka. *Intas Polivet*, *8*(1), pp.112-114.
151. Mamta, A.N., Rajora, V.S., Singh, V.S., and Mrigesh, M., 2013.Prevalence of gastrointestinal parasites with special reference to bursate worm infestation in goats in and around Pantnagar. *Veterinary Practitioner*, *14*, 451-452.
152. Maravi, D.S., Dixit, P. and Dixit, A.K., 2022. Prevalence and Risk Factors of Caprine Gastrointestinal Parasites in and around Rewa, Madhya Pradesh, India. *Indian Journal of Veterinary Sciences and Biotechnology*, *18*(3), pp.125-127.
153. Matsepe, L.G., Molapo, S., Phalatsi, M. and Phororo, M., 2021. Prevalence and fecal egg load of gastrointestinal parasites of Angora goats in four agro-ecological zones in Lesotho. *Veterinary World*, *14*(2), p.339.
154. Maurizio, A., Stancampiano, L., Tessarin, C., Pertile, A., Pedrini, G., Asti, C., Terfa, W., Frangipane di Regalbono, A. and Cassini, R., 2021. Survey on endoparasites of dairy goats in North-Eastern Italy using a farm-tailored monitoring approach. *Veterinary Sciences*, *8*(5), pp.69.
155. Mazyad, S.A. and el-Nemr, H.I., 2002. The endoparasites of sheep and goats, and shepherd in North Sinai Governorate, Egypt. *Journal of the Egyptian society of Parasitology*, *32*(1), pp.119-126.
156. Meena, D., Laha, R. and Goswami, A., 2017. Gastrointestinal parasitism of goats in hilly region of Meghalaya, India. *Veterinary World*, *10*(1), pp.81-85.
157. Mhoma, J.R.L., Kanyari, P.W.N. and Kagira, J.M., 2011. The prevalence of gastrointestinal parasites in goats in urban and peri-urban areas of Mwanza City, Tanzania. *Parasitological Science*, *12*(4), pp.191-196.
158. Miambo, R.D., 2016. Prevalence of gastro-intestinal parasites of livestock and dogs and risk factors for transmission with emphasis on giardia and cryptosporidium in Magude district, Maputo province, Mozambique (Doctoral dissertation), University of KwaZulu-Natal.
159. Minnat, T.R., Alzubaidei, H.H.H. and Al-Ezzy, A.I.A., 2014. Heamatological Changes Associated with Gastrointestinal Parasites Infection in Domestic Animals attended to Outpatient Clinic of Faculty of Veterinary Medicine of Diyala University, Iraq. *International journal of innovation and applied studies*, *9*(3), pp.1266-1274.
160. Mohamaden, W.I., Sallam, N.H. and Abouelhassan, E.M., 2018. Prevalence of Eimeria species among sheep and goats in Suez Governorate, Egypt. *International journal of veterinary science and medicine*, *6*(1), pp.65-72.
161. Mondal, M. M. H.; Qadir, A. N. M. A., 1978. A preliminary investigation on the incidence of coccidial infection in fowls, sheep, goats, and cattle. *Bangladesh Veterinary Journal,* 12(1/4), pp.7-11.
162. More, B.V., Nikam, S.V., Bhamare, N.D.S. and Jaid, E.L., 2011. Percentage prevalence of Eimerian species composition of sheep and goats from beed district, Maharashtra. *Recent Research in Science and Technology*, *3*(8), pp.24-26.
163. Mottelib, A.A., Haroun, E.M., Magzoub, M. and El-Basheer, E., 1992. The effect of gastro-intestinal parasites on blood picture in sheep and goats at Al-Gassim. *Assiut Veterinary Medical Journal*, *28*(55), pp.215-223.
164. Mpofu, T.J., Nephawe, K.A. and Mtileni, B., 2020. Prevalence of gastrointestinal parasites in communal goats from different agro-ecological zones of South Africa. *Veterinary world*, *13*(1), pp.26-32.
165. Muraleedharan, K., 2005. Prevalence of gastrointestinal parasites of livestock in a central dry zone of Karnataka. *Journal of Veterinary Parasitology*, *19*(1), pp.31-33.
166. Nath, S., Dixit, A.K., Jain, R., Agrawal, V., Dongre, S. and Das, G., 2015. Epizootiological studies on gastrointestinal parasites of domestic ruminants in Khandwa district of Madhya Pradesh, India. *Journal of Veterinary Parasitology*, *29*(1), pp.41-46.
167. Nayak, S., Rath, P.K., Panda, S.K., Mishra, B.P., Mishra, R. and Biswal, S.S., 2021. Etiopathological and hematobiochemical profiles in goats with gastrointestinal disorders. *Veterinary World*, *14*(7), pp.1760-1966.
168. Ndarathi, C.M., Waghela, S. and Semenye, P.P., 1989. Helminthiasis in Maasai ranches in Kenya. *Bulletin of animal health and production in Africa,* 7(3), pp.205-208.
169. Nenadović, K., Ilić, T., Jovanović, N., Bugarski, D. and Vučinić, M., 2021. Welfare of Native Goat Breeds in Serbia: Emphasis on Parasitological Infections. *Frontiers in Veterinary Science*, *8*, pp.678880.
170. Ngumbi, N.H., Kassuku, A.A., Karimuribo, E.D., Fitzpatrick, J., Smith, D., Matiko, M.K. and Kambarage, D.M., 2015. Status of helminthosis and coccidiosis in goats and sheep in traditional flocks in Melela Division, Morogoro region, Tanzania. *Tanzania Veterinary Journal*, *30*(2), pp.19-27.
171. Nguyen-Ho-Bao, T., Lu, T.A. and Nguyen, H.H., 2022. Studies on Molecular Characteristics of Eimeria arloingi in Goats (Capra hircus) in Vietnam. *Journal of Science, Engineering and Technology (JSET)*, *10*, pp.19-28.
172. Nikam, S.R., 1999. Species Composition and Relative Prevalence of Eimeria in Sheep and Goats from Marathwada Region Maharashtra. *Ecology, Environment and Conservation*, *5*, pp.211-213.
173. Norton, C.C., 1986. Coccidia of the domestic goat Capra hircus, with notes on Eimeria ovinoidalis and E. bakuensis (syn. E. ovina) from the sheep Ovis aries. *Parasitology*, *92*(2), pp.279-289.
174. Ntonifor, H., Shei, S., Ndaleh, N. and Mbunkur, G., 2013. Epidemiological studies of gastrointestinal parasitic infections in ruminants in Jakiri, Bui Division, Northwest Region of Cameroon. *Journal of Veterinary Medicine and Animal Health*, *5*(12), pp.344-352.
175. Nur Rahifah Inani, R. and Yusof, M., 2018. Seasonal prevalence of gastrointestinal parasitic infections in goats in a commercial farm Kuantan, Malaysia. *Asian Journal of Agriculture and Biology*, *5*(4), pp.455-460.
176. Nwigwe, J.O., Njoku, O.O., Odikamnoro, O.O. and Cosmas, U.A., 2013. Comparative study of intestinal helminths and protozoa of cattle and goats in Abakaliki metropolis of Ebonyi State, Nigeria. *Advances in Applied Science Research*, *4*(2), pp.223-227.
177. O'callaghan, M.G., 1989. Coccidia of domestic and feral goats in South Australia. *Veterinary Parasitology*, *30*(4), pp.267-272.
178. Ola-Davies, O.E., Oyeyemi, M.O., Saba, A.B. and Ajala, O.O., 2002. Prevalence of Eimeria oocysts in West African Dwarf goats at the University of Ibadan farm. *Nigerian Journal of Animal Production*, *29*(2), pp.259-263.
179. Osman, F.A., 2009. Incidence of internal parasites of desert goats in New Valley, Egypt. *Assiut Veterinary Medical Journal*, *55*(*120*), pp.1-10.
180. Padilla, M.A., Baticados, W.N., Desamero, J.M. and Lucas, S.F., 2009. Prevalence and factors associated with Eimeria infection in goats in Laguna, Philippines. *Philippine Journal of Veterinary and Animal Sciences*, *35*(2), pp.108-118.
181. Padmaja, K., Haritha, C. and Anitha, K., 2007. Prevalence of gastrointestinal parasitism in goats. *Intas Polivet*, *8*(1), pp.32-34.
182. Pandit, B.A., 2006. Prevalence of caprine coccidiosis in Kashmir valley. *The Indian Journal of Small Ruminants*, *12*(2), pp.223-226.
183. Papri, P., Chatlod, L.R. and Avasthe, R.K., 2017. Seasonal prevalence of gastrointestinal parasites of goats in North-East Himalayan region of Sikkim, India. *Indian Journal of Animal Sciences*, *87*(5), pp.558-561.
184. Patel, M.D., Nauriyal, D.S., Hasnani, J.J. and Gupta, R.S., 2001. Prevalence of gastrointestinal parasitism in goats maintained under semi-intensive and field management systems. *Indian Journal of Veterinary Medicine*, *21*(2), pp.99-101.
185. Patil, A.S., Gatne, M.L. and Narsapur, V.S., 1990. Comparative observations on coccidia of sheep and goats. *Journal of Bombay Veterinary College*, *2*(2), pp.125-126.
186. Paul, B.T., Jesse, F.F.A., Chung, E.L.T., Che’Amat, A. and Mohd Lila, M.A., 2020. Risk factors and severity of gastrointestinal parasites in selected small ruminants from Malaysia. *Veterinary sciences*, *7*(4), pp.208.
187. Penzhorn, B.L., Rognlie, M.C., Hall, L.L. and Knapp, S.E., 1994. Enteric coccidia of Cashmere goats in southwestern Montana, USA. *Veterinary Parasitology*, *55*(1/2), pp.137-142.
188. Pilarczyk, B., Tomza-Marciniak, A., Pilarczyk, R., Bombik, E., Seremak, B., Udała, J. and Sadowska, N., 2021. A Comparison of the Prevalence of the Parasites of the Digestive Tract in Goats from Organic and Conventional Farms. *Animals*, *11*(9), pp.2581.
189. Priyanka, S., Satyavir, S., Sukhdeep, V. and Sangwan, A.K., 2013. Prevalence of various species of gastro-intestinal helminths and Eimeria in an organized goat farm. *Veterinary Practitioner*, *14*(*2*), pp.302-303.
190. Radavelli, W.M., Pazinato, R., Klauck, V., Volpato, A., Balzan, A., Rossett, J., Cazarotto, C.J., Lopes, L.S., Kessler, J.D., Cucco, D.C. and Tonin, A.A., 2014. Occurrence of gastrointestinal parasites in goats from the Western Santa Catarina, Brazil. *Revista Brasileira de Parasitologia Veterinária*, *23*, pp.101-104.
191. Radfar, M.H., Sakhaee, E., Shamsaddini Bafti, M. and Haj Mohammadi, H., 2011. Study on gastrointestinal parasitic infections of Raeini goats. *Iranian Journal of Veterinary Research*, *12*(1), pp.76-80.
192. Radha, G., Jeyathilakan, N., Gomathinayagam, L., John, M. and Karunanidhi, K., 2004. Sub-clinical Eimeria sp. infection of sheep and goats. *Journal of Veterinary Parasitology*, *18*, pp.59-61.
193. Rahman, H., Papri, P., Bandyopadhyay, S. and Chatlod, L.R., 2012. Epidemiology of gastrointestinal parasitism in goats in Sikkim. *Indian Journal of Animal Sciences*, *82*(4), pp.355-358.
194. Raote, Y. V., Bhandarkar, A.G., Joshi, M. V., Bhagwat, S. S.,1987. Incidence of internal parasites in goats. Livestock Adviser, 12(7), pp.47-48.
195. Regassa, F., Sori, T., Dhuguma, R. and Kiros, Y., 2006. Epidemiology of gastrointestinal parasites of ruminants in Western Oromia, Ethiopia. *International journal of applied Research in Veterinary Medicine*, *4*(1), pp.51-57.
196. Rehman, T., Khan, M.N., Sajid, M.S., Iqbal, Z., Javid, M.T., Riaz, M. and Ahmad, M., 2012. Epidemiology of Eimeria and associated risk factors in goats of district Toba Tek Singh, Pakistan. *Indian Journal of Animal Sciences*, *82*(3), pp.282-285.
197. Rozita, M. H. L.; Chandrawathani, P.; Premaalatha, B.; Erwanas, A. I.; Zaini, C. M.; Jamnah, O.; Nurulaini, R.; Norazura, A. H.; Bohari, J.; Ramlan, M., *2015.* The VRI small ruminant field programme: assessment of parasitic infections in local smallholder farms from 2012-2013. *Malaysian Journal of Veterinary Research, 6(2*), pp.65-72.
198. Ruiz, A., González, J.F., Rodríguez, E., Martín, S., Hernández, Y.I., Almeida, R. and Molina, J.M., 2006. Influence of climatic and management factors on Eimeria infections in goats from semi‐arid zones. *Journal of Veterinary Medicine, Series B*, *53*(8), pp.399-402.
199. Sahin, Ö., Aytekin, İ., Boztepe, S., Keskin, İ., Karabacak, A., Altay, Y. and Bayraktar, M., 2021. Relationships between FAMACHA© scores and parasite incidence in sheep and goats. *Tropical animal health and production*, *53*(*2*), pp.331.
200. Saidi, R., Mimoune, N., Baazizi, R., Khelef, D., Azzouz, M.Y. and Kaidi, R., 2020. Contribution to studying ecto and mesoparasites in goats in Southern Algeria. *Veterinaria*, *69*(*1*), pp.23-29.
201. Saleem, T., Katoch, R., Godara, R., Yadav, A. and Khursheed, A., 2018. Epidemiology of Gastro-Intestinal parasites in small ruminants of Jammu Province. *Indian Journal of Small Ruminants*, *24*(2), pp.357-359.
202. Sangvaranond, A., Lampa, N., Wongdachkajorn, D. and Sritong, D., 2010. Prevalence of helminth parasites and intestinal parasitic protozoa among meat goats raised in private farms in Saraburi Province Thailand. *Kasetsart Veterinarians*, *20*(2), pp.85-95.
203. Sapna, M., Niddhi, A., Munish, B. and Agrawal, D.K., 2010. Prevalence of gastrointestinal parasites in goats. *Indian Veterinary Journal*, *87*(*10*), pp.1033-1034.
204. Secchioni, E., Sgorbini, M. and Perrucci, S., 2016. Gastrointestinal parasites, liver flukes and lungworms in domestic ruminants from central Italy. *Large animals review*, *22*(*6*), pp.195-201.
205. Seddik, S. and Ahmed Mohamed, A.E., 2022. Clinical and Biochemical Assessment of Eimeria Infection in Goats at Sohag Governorate. *Journal of Applied Veterinary Sciences*, *7*(*2*), pp.7-12.
206. Senthilvel, K., Basith, S.A. and Rajavelu, G., 2004. Caprine coccidiosis in Chennai and Kancheepuram districts of Tamil Nadu. *Journal of Veterinary Parasitology*, *18*(2), pp.159-161.
207. Shah, H.L. and Joshi, S.C., 1963. Coccidia (Protozoa: Eimcriidae) of goats in Madhya Pradesh, with descriptions of the sporulated oocysts of eight species. *Journal of Veterinary Research*, 7, pp. 9-20.
208. Shah, H.L., 1965. The occurrence of the coccidium Eimeria christenseni in goats in India. *Current Science*, *34*(19), pp.564-565.
209. Shaheed, H.A. and Al-Azizz, S., 2020. Epidemiological charectrization on Eimeriosis in small ruminants in Bashar city of southern Iraq. *Plant Archives, 20*(*2*), pp. 6010-6014.
210. Sharma, D.K. and Mandal, A., 2013. Factors affecting gastrointestinal parasite infections in goats in semi-arid rural ecosystems in India. *Veterinary Science Development*, *3*(*1*), pp. e5.
211. Sharma, D.K., Agrawal, N., Mandal, A., Nigam, P. and Bhushan, S., 2009. Coccidia and gastrointestinal nematode infections in semi-intensively managed Jakhrana goats of semi-arid region of India. *Tropical and Subtropical Agroecosystems*, *11*(*1*), pp.135-139.
212. Sharma, R.L., Bhattacharya, D., Laha, R., Biswas, J.C. and Rangarao, G.S.C., 1997. Preliminary observations on intestinal coccidiosis in Pashmina (Cashmere) goats in India. *Journal of Applied Animal Research*, *12*(1), pp.107-112.
213. Sharma, D.K., Paul, S., Rout, P.K., Mandal, A., Bhusan, S., Sharma, N. and Kushwah, Y.K., 2017. Caprine coccidiosis in semi-arid India: Dynamics and factors affecting fecal oocysts count. *Journal of Advanced Veterinary and Animal Research*, *4*(1), pp.52-57.
214. Shija, D.S.N., Kusiluka, L.J.M., Chenyambuga, S.W., Shayo, D. and Lekule, F.P., 2014. Animal health constraints in dairy goats kept under smallholder farming systems in Kongwa and Mvomero Districts, Tanzania. *Journal of Veterinary Medicine and Animal Health*, *6*(*11*), pp.268-279.
215. Shivairo, R.S., 2003. The emerging significance of coccidiosis in goats in arid and semi-arid lands (ASAL) of Kenya. *Bulletin of Animal Health and Production in Africa*, 51(1), pp.62-63.
216. Silva, L.M.R.D., Vila-Viçosa, M.J.M., Nunes, T., Taubert, A., Hermosilla, C. and Cortes, H.C.E., 2014. Eimeria infections in goats in Southern Portugal. *Brazilian Journal of Veterinary Parasitology*, *23*, pp.280-286.
217. Silva, L.M.R., Carrau, T., Vila-Viçosa, M.J.M., Musella, V., Rinaldi, L., Failing, K., Cortes, H.C.E., Taubert, A. and Hermosilla, C., 2020. Analysis of potential risk factors of caprine coccidiosis. *Veterinary Parasitology: Regional Studies and Reports*, *22*, pp.100458.
218. Singh, V., Varshney, P., Dash, S.K. and Lal, H.P., 2013. Prevalence of gastrointestinal parasites in sheep and goats in and around Mathura, India. *Veterinary World*, *6*(5), pp.260-262.
219. Singh, A.K., Das, G., Roy, B., Nath, S., Ram, N. and Sahil, K., 2014. Occurence of Coccidial Infections in Goat of Madhya Pradesh. *Environment and Ecology*, *32*(*4*), pp.1313-1315.
220. Singh, E., Kaur, P., Singla, L.D. and Bal, M.S., 2017. Prevalence of gastrointestinal parasitism in small ruminants in western zone of Punjab, India. *Veterinary world*, *10*(1), pp.61-66.
221. Singh, A.K., Shanker, D., Rout, P.K., Kumar, A., Sharma, N. and Kumar, P., 2016. Incidence and haemato-biochemical studies on goats naturally infected with coccidiosis in semi-arid region, India. *Ruminant Science*, *5*(2), pp.257-260.
222. Soliman, M.F. and Zalat, S.M., 2003. Prevalence and intensity of Nematodirus sp. and Eimeria sp. infections in the domestic goats of St. Katherine's Protectorate (Sinai, Egypt): relations with some ecological and biological factors. *Egyptian Journal of Biology*, *5*, pp.78-85.
223. Sorathiya, L.M., Fulsoundar, A.B., Rao, T.K.S. and Kumar, N., 2017. Prevalence and risk factors for gastrointestinal parasitism in traditionally maintained goat flocks of South Gujarat. *Journal of parasitic diseases*, *41*, pp.137-141.
224. Soundararajan, C., Nagarajan, K., Satish, A.C. and Prakash, M.A., 2019. Use of tamarind seed coat powder for controlling coccidiosis in goats. *Indian Journal of Small Ruminants*, *25*(2), pp.247-250.
225. Squire, S.A., Robertson, I.D., Yang, R., Ayi, I. and Ryan, U., 2019. Prevalence and risk factors associated with gastrointestinal parasites in ruminant livestock in the Coastal Savannah zone of Ghana. *Acta Tropica*, *199*, pp.105126.
226. Stadalienė, I., Petkevičius, S. and Šarkūnas, M., 2014. The impact of grazing management on seasonal activity of gastrointestinal parasites in goats. *Helminthologia*, *51*, pp.103-111.
227. Tan, T.K., Chandrawathani, P., Low, V.L., Premaalatha, B., Lee, S.C., Chua, K.H., Sharma, R.S.K., Romano, N., Tay, S.T., Quaza, N.H.N. and Lim, Y.A.L., 2017. Occurrence of gastro-intestinal parasites among small ruminants in Malaysia: highlighting Dicrocoelium infection in goats. *Tropical Biomedicine*, *34*, pp.963-969.
228. Tauseef-ur-Rehman, M.N.K., Khan, I.A. and Ahmad, M., 2011. Epidemiology and economic benefits of treating goat coccidiosis. *Pakistan Veterinary Journal*, *31*(3), pp.227-230.
229. Tchoumboue, J., Awah-Ndukum, J.J. and Tong, J.C., 2000. A survey of gastrointestinal parasites in sheep and goats of Western Highlands of Cameroon. *Bulletin of Animal Health and Production in Africa*, *48*(4), pp.250-253.
230. Terefe, D., Demissie, D., Beyene, D. and Haile, S., 2012. A prevalence study of internal parasites infecting Boer goats at Adami Tulu agricultural research center, Ethiopia. *Journal of Veterinary Medicine and Animal Health*, *4*(2), pp.12-16.
231. Thangathurai, R., Rao, D.G.K. and Reddy, P.T., 2003. Prevalence of enteric parasitism in sheep and goats in and around Bidar. *Indian Veterinary Journal,* 80(1), pp.72-73*.*
232. Thedford, T.R., Kelemogile, K.M., Worman, F.D. and Baathodi, J.A., 1990. A study of internal parasite egg counts in goats from three villages in the Tutume agricultural district, Botswana, 1988-90. *ATIP Working Paper, Department of Agricultural Research, Ministry of Agriculture,*31, pp. 6.
233. Tiwari, A., Roy, S., Galdhar, C.N. and Upadhyay, S.R., 2003. Prevalence of coccidiosis in goats of Durg district of Chattisgarh. *Indian veterinary journal*, *80*(6), pp.579-580.
234. Tongson, M.S., Manuel, M.F. and Eduardo, S.L., 1981. Parasitic fauna of goats in the Philippines. *Philippine Journal of Veterinary Medicine*, *20*(1), pp.1-37.
235. Tumusiime, M., Ndayisenga, F. and Ntampaka, P., 2022. Prevalence of gastrointestinal nematodes, cestodes, and protozoans of goats in Nyagatare District, Rwanda. *Veterinary Medicine: Research and Reports*, *13*, pp.339-349.
236. Varsha, B., Pratibha, J., Shraddha, B., Kavita, S. and Maske, D.K., 2010. Prevalence of coccidiosis in goats in Nagpur region of Maharashtra. *Indian Journal of Field Veterinarians*, *5*(*3*), pp.39-40.
237. Vasilková, Z., Krupicer, I., Legáth, J., Kovalkovicova, N. and Pet’ko, B., 2004. Coccidiosis of small ruminants in various regions of Slovakia. *Acta Parasitologica*, *49*(*4*), pp.272-275.
238. Vercruysse, J., 1982. The coccidia of sheep and goats in Senegal. *Veterinary Parasitology*, *10*(*4*), pp.297-306.
239. Verma, R.K. and Jaiswal, S., 2017. Prevalence of gastrointestinal parasites of ruminants in and around Faizabad (Uttar Pradesh), India. *Journal of Experimental Zoology India*, *20*(*2*), pp.683-688.
240. Verma, R., Sharma, D.K., Gururaj, K., Paul, S., Banerjee, P.S. and Tiwari, J., 2017. Molecular epidemiology and point mutations in ITS1 and 18S rDNA genes of Eimeria ninakohlyakimovae and E. christenseni isolated from Indian goats. *Veterinary Parasitology: Regional Studies and Reports*, *9*, pp.51-62.
241. Verma, R., Sharma, D.K., Paul, S., Gururaj, K. and Sarthi, P., 2018a. Prevalence of gastrointestinal parasitic infections in Barbari goat under semi-arid region. *Environment and Ecology*, *36*(*1*), pp.43-45.
242. Verma, R., Sharma, D.K., Paul, S., Gururaj, K., Dige, M., Saxena, V.K., Rout, P.K., Bhusan, S. and Banerjee, P.S., 2018b. Epidemiology of common gastrointestinal parasitic infections in goats reared in semi-arid region of India. *Journal of Animal Research*, *8*(1), pp.39-45.
243. Vihan, V.S., Singh, N. and Singh, S.V., 1988. Prevalence of clinical coccidiosis in kids under semi-arid conditions. *Indian Journal of Animal Sciences*, *58*(10), pp.1178-1180.
244. Wang, C.R., Xiao, J.Y., Chen, A.H., Chen, J., Wang, Y., Gao, J.F. and Zhu, X.Q., 2010. Prevalence of coccidial infection in sheep and goats in northeastern China. *Veterinary Parasitology*, *174*(3/4), pp.213-217.
245. Waruru, R.M., Mutune, M.N. and Otieno, R.O., 2005. Gastrointestinal parasite infections of sheep and goats in a semi-arid area of Machakos District, Kenya. *Bulletin of animal health and production in Africa*, *53*(*1*), pp.25-34.
246. Waruiru, R.M., Githigia, A.M. and Nginyi, J.M., 1991. The prevalence of Coccidia of goats in Ol’Magogo farm in Kenya. *Bulletin of Animal Health and Production in Africa*, *39*(*2*), pp.247-249.
247. Waruiru, R.M., Gichanga, E.J., Kimoro, C.O. and Karanu, F.N., 1994. Prevalence of gastrointestinal nematodes, coccidia and lungworms in Ol'Magogo dairy goats. *Bulletin of animal health and production in Africa*, *42* (*4*), pp.291-295.
248. Win, S.Y., Win, M., Thwin, E.P., Htun, L.L., Hmoon, M.M., Chel, H.M., Thaw, Y.N., Soe, N.C., Phyo, T.T., Thein, S.S. and Khaing, Y., 2020. Occurrence of gastrointestinal parasites in small ruminants in the central part of Myanmar. *Journal of Parasitology Research*.
249. Windsor, P.A., Nampanya, S., Putthana, V., Keonam, K., Johnson, K., Bush, R.D. and Khounsy, S., 2018. The endoparasitism challenge in developing countries as goat raising develops from smallholder to commercial production systems: A study from Laos. *Veterinary parasitology*, *251*, pp.95-100.
250. Woji, A.Y., Little, D.A. and Ikwuegbu, O.A., 1994. Prevalence of coccidial infections in the West African Dwarf goat in the subhumid zone of Nigeria. *Tropical Animal Health and Production*, *26*(*1*), pp.1-6.
251. Wuthijaree, K., Tatsapong, P. and Lambertz, C., 2022. The prevalence of intestinal parasite infections in goats from smallholder farms in Northern Thailand. *Helminthologia*, *59*(*1*), pp.64-73.
252. Yadav, A., Khajuria, J.K. and Raina, A.K., 2006. Seasonal prevalence of gastrointestinal parasites in sheep and goats of Jammu. *Journal of Veterinary Parasitology*, *20*(*1*), pp.65-68.
253. Yusof, A.M. and Isa, M.L.M., 2016. Prevalence of gastrointestinal nematodiasis and coccidiosis in goats from three selected farms in Terengganu, Malaysia. *Asian Pacific Journal of Tropical Biomedicine*, *6*(*9*), pp.735-739.
254. Zhao, G.H., Lei, L.H., Shang, C.C., Gao, M., Zhao, Y.Q., Chen, C.X. and Chen, D.K., 2012. High prevalence of Eimeria infection in dairy goats in Shaanxi province, northwestern China. *Tropical animal health and production*, *44(5)*, pp.943-946.
255. Zvinorova, P.I., Halimani, T.E., Muchadeyi, F.C., Matika, O., Riggio, V. and Dzama, K., 2016. Prevalence and risk factors of gastrointestinal parasitic infections in goats in low-input low-output farming systems in Zimbabwe. *Small Ruminant Research*, *143*, pp.75-83.
