# Supplementary material for: Global prevalence of Eimeria species in goats: a systematic review and meta-analysis
Source: Front Vet Sci. 2025 Jan 23;11:1537171. doi: 10.3389/fvets.2024.1537171 (PMC11800301; doi:10.3389/fvets.2024.1537171)
Supplement: Supplementary file 2 [file Table_2.DOCX]

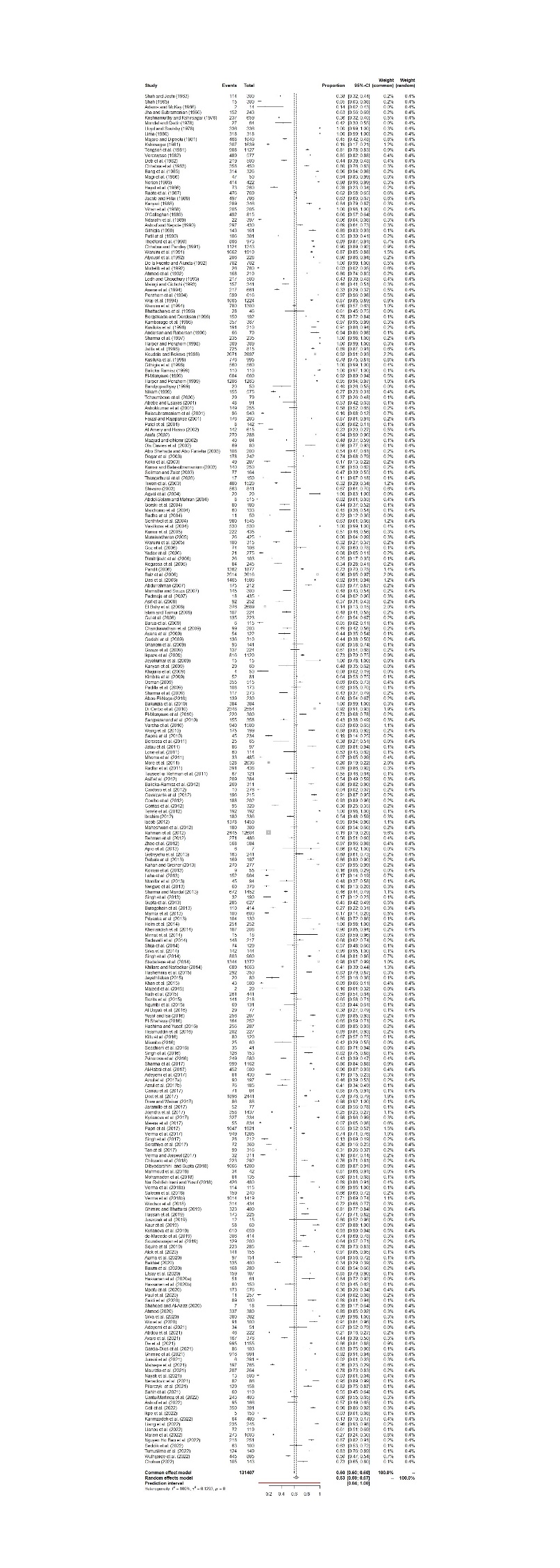


FIGURE S1.

Forest plot showing the prevalence estimates of *Eimeria* spp. in goats for each selected study. Weightage given to each study by random effects and fixed (common) effect models has been shown for comparison. “Total” refers to the number of goats in each study, while “Events” refers to the number of goats tested positive for *Eimeria* spp. “Proportion” reports the prevalence of *Eimeria* spp. for each study. The length of the horizontal line represents the 95% CI. The estimated global prevalence is shown as a diamond at the bottom of the plot. The length of the red line is the 95% prediction interval.

FIGURE S2.

Geographical distribution of the global prevalence of *Eimeria* spp. in goats


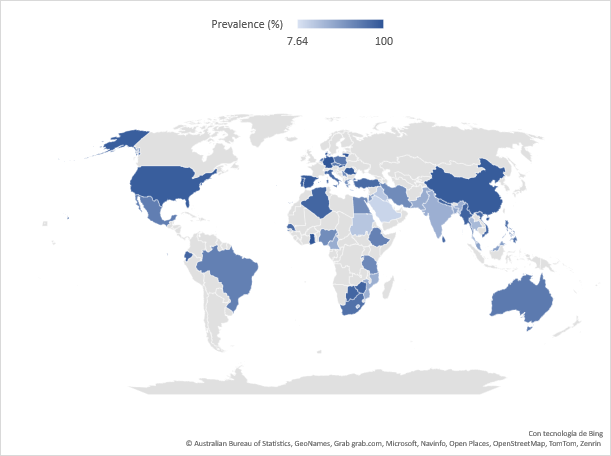


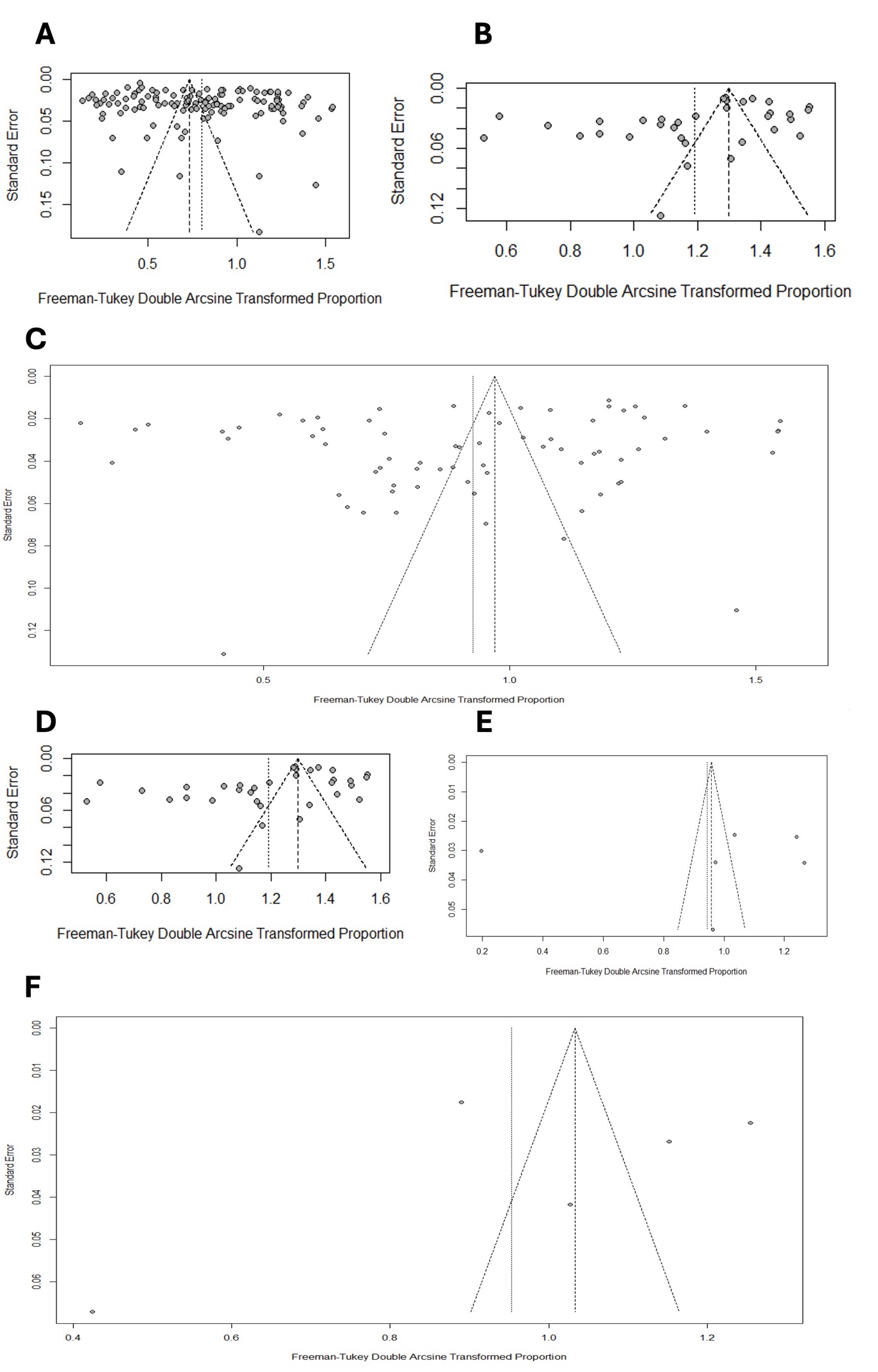


FIGURE S3.

Funnel plots showing publication biases in Asia (A), Europe (B), Africa (C), North America (D), South America (E) and Oceania (F).


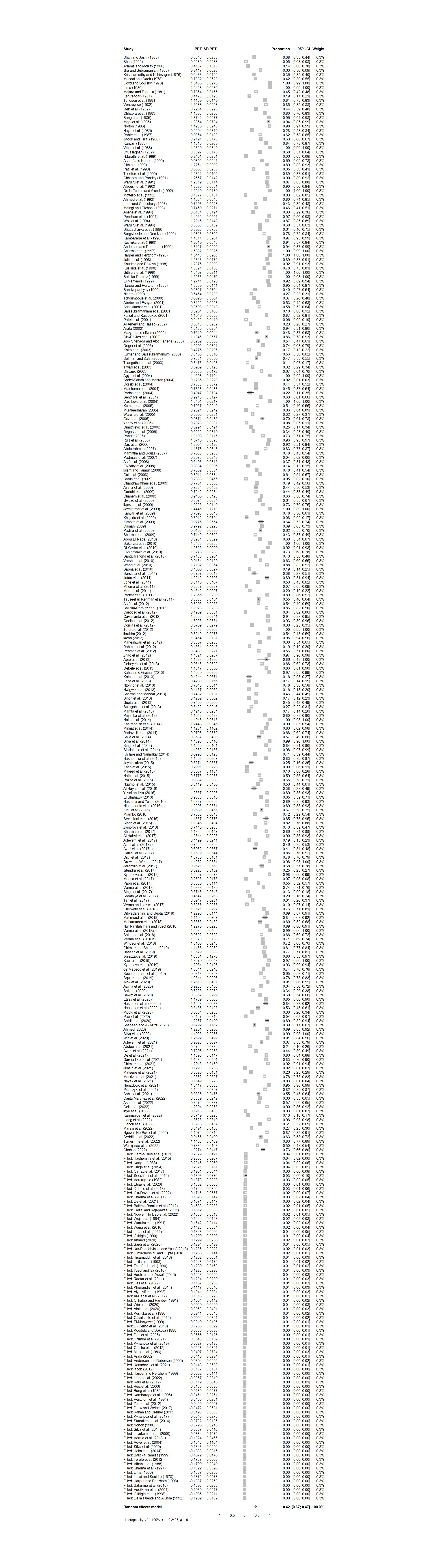
 FIGURE S4**.**

Forest plot showing the trim and fill test.


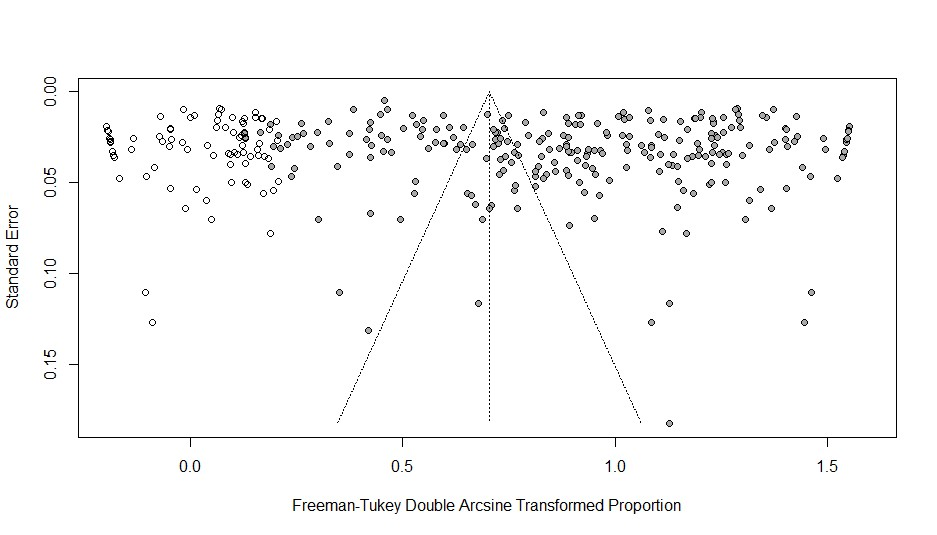
FIGURE S5.

Trim and fill funnel plot showing the number of studies missing from the meta-analysis. White circles are augmented data. Grey dots are eligible studies included in the analysis.


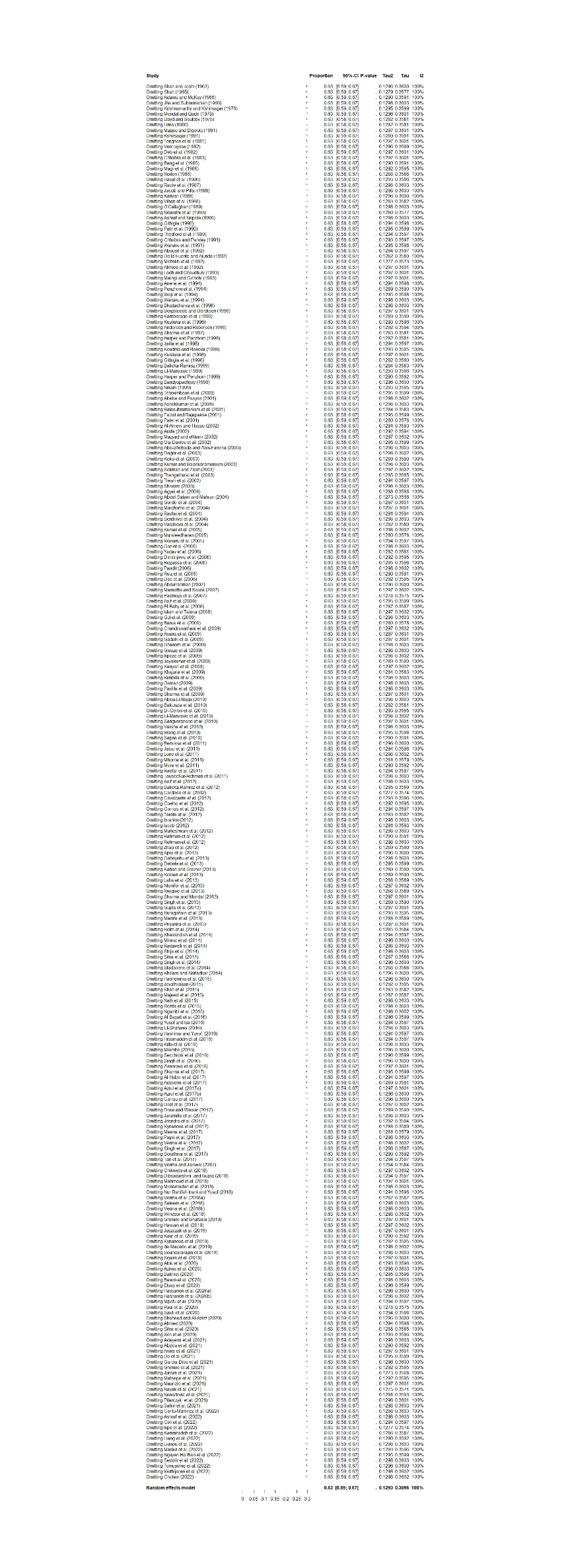
FIGURE S6.

Sensitivity analysis. After removing one study at a time, the remaining studies were re-combined using a random effects model to verify the impact of a single study on the overall results. Tau^2^: estimated amount of total heterogeneity, *Tau* is an estimate of the standard deviation of the distribution of true effect sizes, I^2^: total heterogeneity/ total variability.
